# Stability and Mitochondrial Localization of a Highly Cytotoxic Organogold(III) Complex with Diphosphine Ancillary Ligand in Lung Cancer Cells

**DOI:** 10.1002/anie.202422763

**Published:** 2025-03-27

**Authors:** Hester Blommaert, Clément Soep, Edwyn Remadna, Héloïse Dossmann, Murielle Salomé, Olivier Proux, Isabelle Kieffer, Jean‐Louis Hazemann, Sylvain Bohic, Michèle Salmain, Benoît Bertrand

**Affiliations:** ^1^ Institut Néel CNRS Université Grenoble Alpes 25 Avenue des Martyrs Grenoble 38042 France; ^2^ BM16/FAME beamline The European Synchrotron Radiation Facility 71 avenue des Martyrs Grenoble F‐38000 France; ^3^ Institut Parisien de Chimie Moléculaire, IPCM Sorbonne Université, CNRS 4 Place Jussieu Paris 75005 France; ^4^ ID16A Beamline The European Synchrotron Radiation Facility 71 avenue des Martyrs Grenoble Cedex 9 F‐38043 France; ^5^ Observatoire des Sciences de l'Univers de Grenoble CNRS IRD INRAE Météo France, Université Grenoble Alpes 25 rue des Martyrs Grenoble Cedex 9 38042 France; ^6^ UA7 STROBE INSERM Synchrotron Radiation for Biomedicine Université Grenoble Alpes 2280 rue de la piscine Saint Martin d'Hères 38400 France

**Keywords:** Antitumor agent, Bioinorganic chemistry, Gold, Localization, Speciation

## Abstract

We present a comprehensive study on the chemical reactivity in the gas phase, with amino acids and peptides, and in the cell, the anticancer activity and localization of a series of seven cationic biphenyl gold(III) complexes with aryl, alkyl, and chiral diphosphine ancillary ligands. Despite some structural differences, all the complexes similarly featured high stability toward reduction or ligand exchange in cell‐free conditions. The biphenyl Au(III) complex including the 1,2‐diphenylphosphinoethane (dppe) ligand manifested the same high stability in a cellular setting, as attested by a combination of cryo‐Synchrotron Radiation‐X‐Ray Fluorescence (cryo‐SR‐XRF) nano‐imaging and cryo‐Synchrotron Radiation‐X‐ray Absorption Spectroscopy (cryo‐SR‐XAS) measurements. Tandem cryo‐SR‐XRF elemental mapping and confocal fluorescence microscopy demonstrated the selective accumulation of the dppe complex in mitochondria. This represents the first study of the speciation and distribution of an organogold(III) complex in cancer cells.

## Graphical abstract

The combination of cryo‐synchrotron radiation‐X‐ray fluorescence (cryo‐SR‐XRF), cryo‐synchrotron radiation‐X‐ray absorption (cryo‐SR‐XAS) and high‐resolution mass spectrometry demonstrates the high intracellular stability of a biphenyl gold(III) diphosphine complex indicating the native organogold cation as the active species. A combination of in‐cell labeling and cellular gold mapping by cryo‐SR‐XRF indicates the mitochondrial accumulation of the stable cationic gold complex.

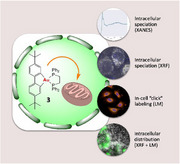

Organogold(III) complexes have recently attracted growing interest for their anticancer activity, both in cell cultures and in vivo.^[^ ^1^ ^−^ ^3^ ^]^ This progress is largely due to advances in designing effective molecules by carefully selecting ligands that stabilize the inherently unstable +3 oxidation state of gold.^[^ ^4^, ^5^ ^]^ Furthermore, advanced proteomics techniques have also been employed to profile the protein targets of several gold compounds and assess the associated cellular responses.^[^ ^6^ ^−^ ^8^ ^]^ Unfortunately, information on the transformations of gold(III) complexes occurring in biological environments, such as ligand exchange and/or reduction to Au(I)/Au(0) in cells, is still scarce, preventing the molecular mechanisms of action of these complexes to be fully established. Synchrotron radiation‐based techniques, i.e. X‐ray fluorescence (SR‐XRF) microscopy and X‐ray absorption spectroscopy (SR‐XAS) have brought essential clues regarding the localization and the speciation of a variety of metal‐based drugs.^[^ ^9^ ^−^ ^12^ ^]^ Analysis of cryo‐fixed cells by a cryo‐SR‐XRF nanoprobe allows multi‐elemental mapping in a frozen‐hydrated state close to the cell native state at nanometer‐scale resolution and in a reasonable time.^[^ ^13^ ^]^ The major advantages of SR‐XRF are i) “label‐free” imaging of the metal that bypasses the incorporation of a fluorescent dye that generally introduces a strong bias in the localization of the metal complex^[^ ^14^ ^]^ and ii) high sensitivity compatible with biologically relevant concentrations. In addition, the ease of sample preparation compared to other imaging techniques such as nanoSIMS makes cryo‐SR‐XRF the method of choice for cell structural analysis.^[^ ^15^ ^]^ For example, selective accumulation of a cationic Ir(III) complex inside mitochondria was visualized by SR‐XRF, while a broader distribution (mitochondria, actin bundles, and nucleus) was observed for a related neutral Ir(III) complex.^[^ ^16^, ^17^ ^]^ On the other hand, the X‐ray absorption near edge structure (XANES) region of XAS gives invaluable information on the valence, geometry, and oxidation state of atoms.^[^ ^18^ ^]^ For instance, XANES allowed us to pinpoint the oxidation state of platinum in bulk cell samples exposed to various Pt(IV) complexes.^[^ ^19^ ^−^ ^24^ ^]^ In the context of metallodrug development, the combination of XANES with XRF (nanoprobe‐XAS) revealed the local speciation of a half‐sandwich osmium(II) complex in cryofixed and dehydrated A2780 cells.^[^ ^25^ ^]^

To the best of our knowledge, none of these techniques have been used so far to probe the localization and speciation of gold complexes in a cellular context, even though the interaction of gold compounds with proteins has been investigated by XANES or EXAFS,^[^ ^26^ ^−^ ^28^ ^]^ and showed that reduction and ligand exchanges are key points of the activity and deactivation of gold complexes.^[^ ^29^, ^30^ ^]^ Herein, we investigate the chemical reactivity, the anticancer activity, and the in‐cell speciation of an array of biphenyl‐based gold(III) diphosphine complexes **1–7** (Figure 1) by cryo‐SR‐XRF, confocal fluorescence microscopy, cryo‐SR‐XANES and high‐resolution mass spectrometry (HRMS).

**Figure 1:**
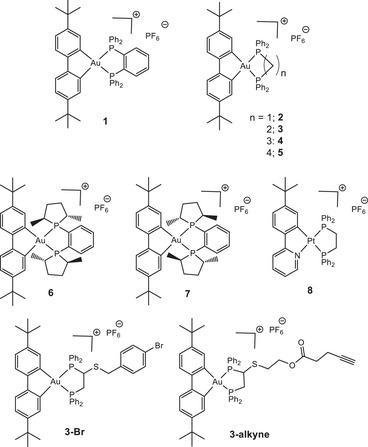
Structure of Au(III) and Pt(II) complexes **1**–**8**, **3‐Br** and **3‐alkyne**.

The combination of all these techniques strongly suggests that the high antiproliferative activity of these complexes mainly stems from the native cationic species.

The [(C^C)Au(P^P)]PF_6_ complexes **1–7** as well as the isoelectronic and structurally similar phenylpyridine Pt(II) (dppe) analog **8** (Figure 1) were synthesized following a previously reported procedure using aryl‐, alkyl‐ and chiral diphosphine ligands.^[^ ^31^ ^]^ Initial screening of complexes **1–8** at 1 and 10 µm determined a marked dependence of the diphosphine ligand structure on human lung adenocarcinoma (A549) cell growth inhibition (Figure S1). Indeed, the Au(III) and Pt(II) complexes with 1,2‐diphenylphosphinobenzene (**1**), dppe (**3** and **8**) or 1,3‐diphenylphosphinopropane (**4**) ligands appeared to be highly active, in contrast to the complexes bearing diphosphine ligands with shorter (**2**) or longer (**5**) alkyl bridges which led to a reduced and dose‐independent antiproliferative activity. Enantiomeric complexes **6** and **7** showed similar activities, suggesting a poor impact of the stereochemistry on the anticancer properties. Moreover, Au complexes **1**, **3**, **4** and platinum complex **8** further demonstrated a highly potent antiproliferative activity on both A549 cells and “triple negative” breast cancer cells MDA‐MB‐231 as well as on non‐cancerous epithelial breast cells MCF‐10A with EC_50_ in the nanomolar range with up to 7‐fold increase of activity compared to the free diphosphines as measured by a classical MTT assay (Table S1 and Figures S2–S5) after 72 h. Complex **3** was the most active gold complex in the series with EC_50_ = 0.05 ± 0.01, 0.08 ± 0.01, and 0.18 ± 0.01 µm toward A549, MDA‐MB‐231 and MCF‐10A cells respectively, i.e. very similar to EC_50_ reported for related biphenyl Au(III) complexes^[^ ^32^, ^33^ ^]^ Interestingly, while complex **3** showed high cytotoxicity on A549 cell already after 4 h of incubation (EC_50_ = 1.2 ± 0.1 µm), the corresponding dppe ligand did not show any toxicity up to 50 µm at this short incubation time (Table S1 and Figure S6) suggesting that the activity of **3** does not stem from the decoordination of the dppe ligand.

Mass spectrometry (MS) and ^31^P{^1^H} NMR spectroscopy experiments were subsequently conducted to examine the intrinsic stability of these complexes. For this purpose, the ability of complexes **1**–**8** to react with selected amino acids or with up to 50 equivalents of GSH or N‐acetyl cysteine (NAC) was initially tested after 24 h at 37 °C. Despite marked differences in terms of cycle constraint in the solid state structures among the Au‐diphosphine metallacycles of complexes **1** to **5** (P‐Au‐P angle from 69.8° to 95.1°, for **2** and **5** respectively),^[^ ^31^ ^]^ no traces of reaction products could be detected upon reaction with 1 eq. of amino acids by MS or ^31^P{^1^H} NMR (Figures S7–S15). These results are in line with literature data on structurally related complexes^[^ ^32^ ^−^ ^34^ ^]^ but contrast with biphenyl Au(III) diimine complexes that undergo facile N^N ligand exchange in the presence of the same amino acids.^[^ ^35^ ^]^ Moreover, no new species were detected by MS upon reaction of complexes **1**–**8** with up to 50 eq. of GSH. In addition, ^31^P{^1^H} NMR analysis of mixtures of **3** and up to 50 eq. of NAC indicated that at least 80 % of the complex remained unchanged (Figures S16–S24). These first observations were completed by the assessment of the complexes' stability in the gas phase toward ligand dissociation.

Following a MS‐based procedure described elsewhere,^[^ ^36^ ^]^ mass‐selected complexes were subjected to collisional activation with N_2_ using Higher Collision Dissociation (HCD), available on orbitrap instruments. The resulting MS/MS spectra were recorded over a large range of HCD collision energies presented in Figure 2 for complex **3** and in Figures S25–S31, for the others. Interestingly, complexes **1**–**7** displayed the same behavior with all undergoing conversion of the [(C^C)Au(P^P)]^+^ cations to the corresponding [Au(P^P)]^+^ fragments through reductive elimination of the biphenyl ligand (Figure 2; Figures S25–S30 and Table S2). On the other hand, the Pt(II) complex **8** was found to undergo more conventional fragmentation through (C^N) ligand decoordination without any reduction (Figure S31). The survival yield (SY) curves, representing the normalized intensities of the precursor ion vs. the HCD collision energy were then plotted. Simulations of these curves can be done using an appropriate kinetic model based on Rice–Ramsperger–Kassel–Marcus (RRKM) theory (see Supporting Information for details),^[^ ^36^ ^]^ and finally lead to the evaluation of the critical energies E_0_ for dissociation, i.e. the minimum energy requested for the dissociation to take place. For the reductive elimination of the biphenyl ligand, these energies are quite large from 1.83 eV (**5**) eV up to 2.75 eV (**1**) (Table S2). This highlights the remarkable stability of the [(C^C)Au(P^P)]⁺ scaffold, suggesting potential intracellular stability for these complexes.

**Figure 2:**
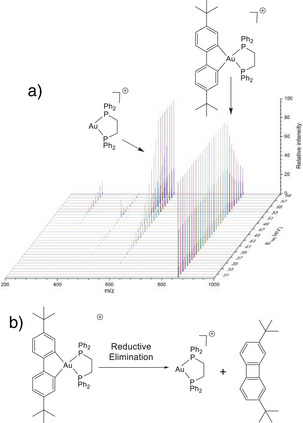
a) MS/MS HCD spectra of the [**3**‐PF_6_]^+^ (m/z 859.3) cation for an HCD activation energy E_lab_ comprised between 0 and 35 eV; b) Reductive elimination reaction of the (C^C) ligand from the [**3**‐PF_6_]^+^ cation.

Based on these data, we investigated the intracellular speciation and localization of the most active complex **3** in A549 cells. Inspired by previous works on bromine‐labeled Pt(II)^[^ ^37^ ^]^ and Os(II)^[^ ^38^, ^39^ ^]^ metallodrugs or iodine‐labeled Re(I) complex,^[^ ^40^ ^]^ we prepared a bromine‐labeled analog of **3** (**3‐Br**, Figure 1) using a hydrothiolation reaction on [(C^C)Au(dppv)]^+^ (dppv = *cis‐*1,2‐diphenylphosphinoethylene) recently developed in our group.^[^ ^41^ ^]^ The labeled compound **3‐Br** showed an antiproliferative activity against A549 cells in a similar range to that of **3** (EC_50_ = 0.11 ± 0.02 µm). We performed SR‐XRF nanoimaging of frozen‐hydrated A549 cells previously incubated with 1 µm of **3‐Br** for 4 h at beamline ID16A of the European Synchrotron Radiation Facility (ESRF).^[^ ^42^ ^]^ The elemental maps for gold and bromine as well as selected endogenous elements were established at 50‐nm pixel size resolution (Figure 3a; Figure S32).^[^ ^43^ ^]^

**Figure 3:**
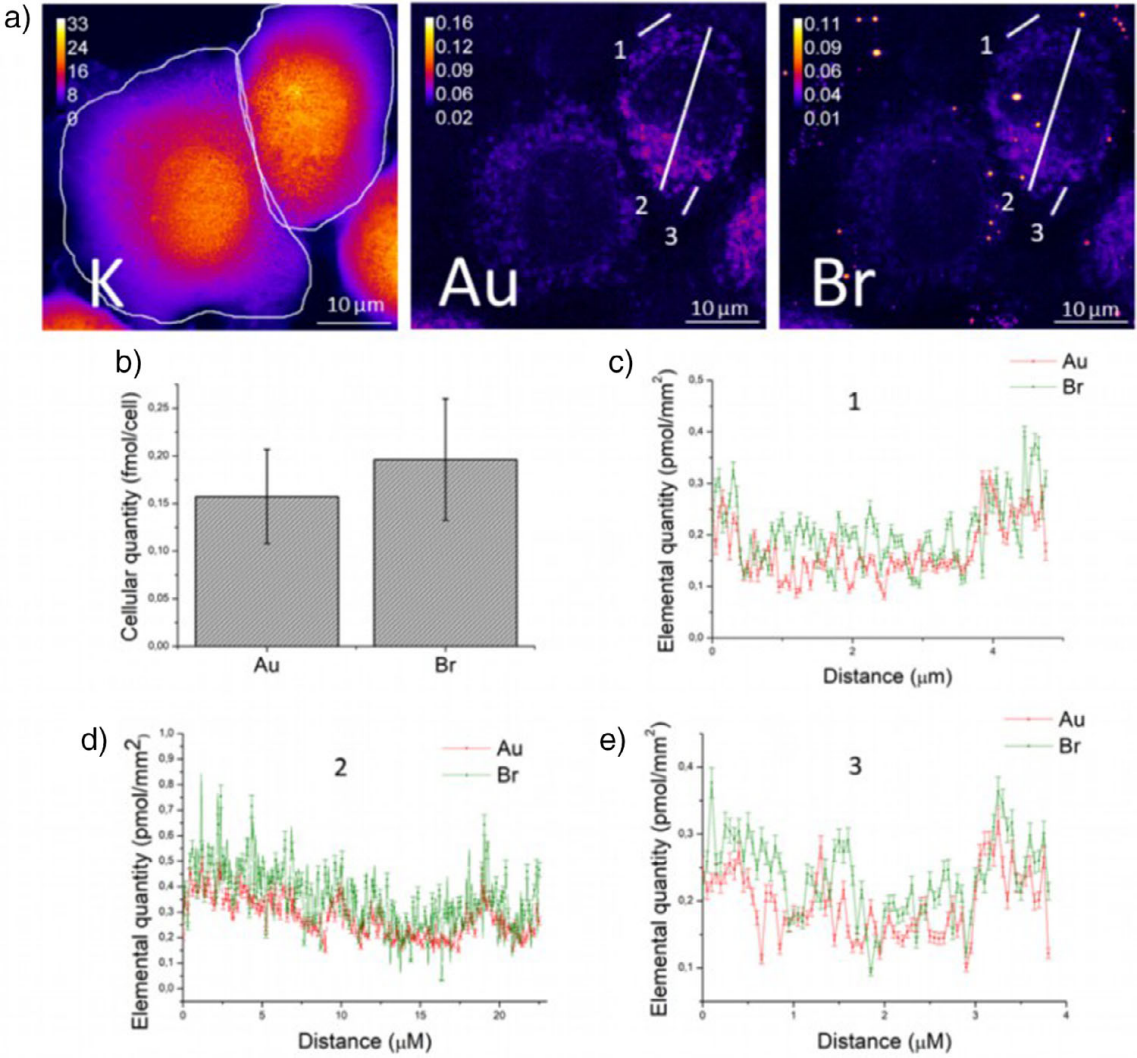
a) Cryo‐SR‐XRF elemental maps of K, Au, and Br in A549 cells treated with 1 µM of **3‐Br** for 4 h recorded at a resolution of 50 nm pixel^−1^ [100 ms]. Elemental contents are given in ng/mm^2^ and are encoded according to the color bar with an instrumental standard error of 6 % on each pixel; b) Absolute quantities + standard error of Au and Br in three recorded cells in ROIs defined according to K‐rich areas; c–e) Spatial evolution of the absolute quantities + standard errors of Au and Br along three arbitrary lines (labeled 1–3) on cryo‐SR‐XRF elemental maps of Au and Br for a **3‐Br**‐treated cell. [ImageJ software].

Absolute quantification of gold and bromine in three recorded cells was performed in regions of interest (ROIs) defined on the corresponding potassium maps since potassium is homogeneously distributed in cells (Figure 3a).^[^ ^13^ ^]^ Quantities of Au and Br were respectively equal to 0.16 ± 0.05 and 0.20 ± 0.06 fmol/cell, giving an Au/Br ratio close to the actual 1:1 ratio in complex **3‐Br** (Figure 3b). The quantitative distribution of Au and Br along three lines (1, 2, and 3 defined on the images in Figure 3a) arbitrarily drawn on the Au and Br elemental maps of the cell with maximum signal intensity showed a 1:1 Au/Br atomic ratio in most pixels, highlighting that both elements colocalize at the subcellular scale (Figure 3c–e), although minor dissociation of the diphosphine ligand could not be ruled out. This finding implies that coordination of the Au ion by the diphosphine ligand is maintained inside the cells.

The speciation of complex **3** was further probed using High Energy Resolution Fluorescence Detected (HERFD)‐XANES spectroscopy, a technique that has already proven effective in providing speciation information for mercury and selenium species at biologically relevant concentrations in cells and tissues.^[^ ^44^ ^−^ ^46^ ^]^ Prior to measurement, we incubated A549 cells with or without 1 µM of **3** for 4 h. We recorded XANES spectra at the Au L_III_‐edge (11.919 keV) of the frozen A549 cells at beamline BM16 (ESRF).^[^ ^47^ ^]^ Untreated control cells lead to a very weak signal in agreement with the very low natural gold content (Figure S33). We also recorded spectra of **3** dissolved in culture medium (DMEM). Additionally, we recorded reference spectra (Figure 4b) of complex **3**, and complexes **Au1‐4** (Figure 4a), which mimic potential metabolites of **3** in the cell. The structure of these complexes was inspired by the HCD results shown above (Figure 2). Their formation results either from the reductive elimination of the biphenyl ligand (**Au1‐3**) or from the substitution of the diphosphine ligand by sulfur‐containing ligands (**Au4**). Moreover, the established cytotoxicity of compounds **Au1**–**4** makes their selection highly relevant.^[^ ^48^ ^−^ ^51^ ^]^ We simulated a spectrum with the FDMNES‐code, based on the XRD structure of **3**. The simulated spectrum corresponds to the reference spectrum of **3**, confirming that the experimental spectrum of **3** represents well its structure (Figure 4b, see experimental section for more details).

**Figure 4:**
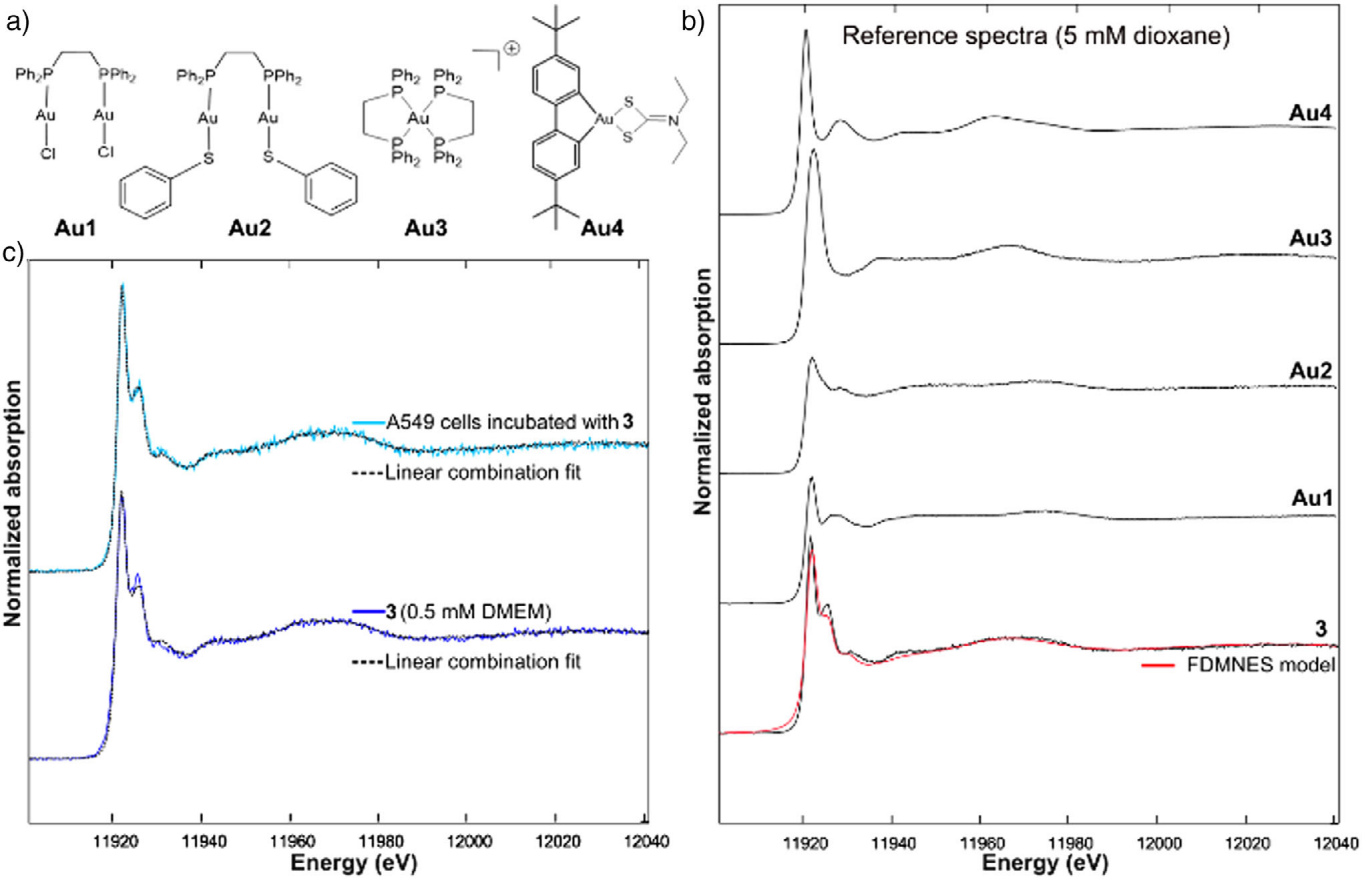
a) Structure of complexes **Au1**‐**4** mimicking potential metabolites of **3**; b) Normalized reference XANES spectra of **3** and **Au1**‐**4** dissolved in dioxane (5 mM), and spectrum of **3** modeled with FDMNES code (based on the XRD structure of **3**); c) Normalized XANES spectrum of frozen‐hydrated A549 cells treated with 1 µm of **3** for 4 h and cell culture medium (DMEM) containing 0.5 mm of **3** and their respective best linear combination fit. All spectra were recorded at 10 K in fluorescence mode at the Au L_III_‐edge.

The XANES spectra of the references **Au1**‐**4** and complex **3** exhibit large differences, either in the bands position relative to the main white‐line band or in their intensities (Figure 4b). The spectra obtained for complex **3** in dioxane, in DMEM, or in treated cells present the same features (Figure 4b,c). This close similarity is corroborated by a linear combination fit with the reference spectra that indicated that 80 to 84 % of Au was complexed as **3** in the A549 cells (Figure S34, Table S3). In DMEM, 91 to 100 % of Au was complexed as **3**. Hence, the majority of complex **3** has remained structurally intact in the cells and in the culture medium. These findings were supported by an HRMS analysis of the cytoplasmic fraction of A549 cancer cells incubated with 1 µm of complex **3** for 4 h. An intense ion signal at m/z 859.28876 was indeed attributed to the [**3**‐PF_6_]^+^ cation, confirming the presence of intact complex **3** in the cell (Figure S35). Taken together, although limited decoordination of the diphosphine ligand cannot be ruled out, these results strongly suggest the intracellular stability of the [(C^C)Au(P^P)]^+^ cationic architecture within the first 4 h of incubation. Thus, in contrast to what was observed for (C^N^C) cyclometalated Au(III) dimers with monodentate bridging diphosphines,^[^ ^52^ ^]^ the antiproliferative activity rather originates from the native [(C^C)Au(P^P)]^+^ cations than from the delivery of toxic diphosphines. Besides, such stability is in good agreement with data gathered for Au(I) complexes with chelating diphosphine ligands^[^ ^49^ ^−^ ^51^, ^53^ ^]^

Delocalized lipophilic cations (DLCs) are reported to accumulate preferentially in mitochondria due to the negative potential of the mitochondrial membrane.^[^ ^54^, ^55^ ^]^ The heterogenous distribution of Au atoms illustrated by the cryo‐SR‐XRF Au map in Figure 3a suggests a tropism of complex **3** for specific cellular compartments. Comparison between the Au and the Zn maps (Figure S32) with the Zn‐rich region representative of the cell nucleus^[^ ^13^ ^]^ indicates that the intracellular distribution of gold is consistent with a perinuclear localization of complex **3**. To identify the preferential zones of accumulation of complex **3‐Br**, correlative cryo‐fluorescence/SR‐XRF microscopy was used, being a well‐established method to study the intracellular distribution of metal complexes.^[^ ^56^ ^]^ Four ROIs were selected within a single treated cell and imaged at a higher spatial resolution of 30 nm (Figure 5a, regions 1–4). Comparison between the Au cryo‐SR‐XRF images and the cryo‐optical fluorescence microscopy images of the same cell stained with the fluorescent dye MitoTracker Green® revealed colocalization of the Au‐rich sites and the mitochondria (Figure 5a; Figure S36)

**Figure 5:**
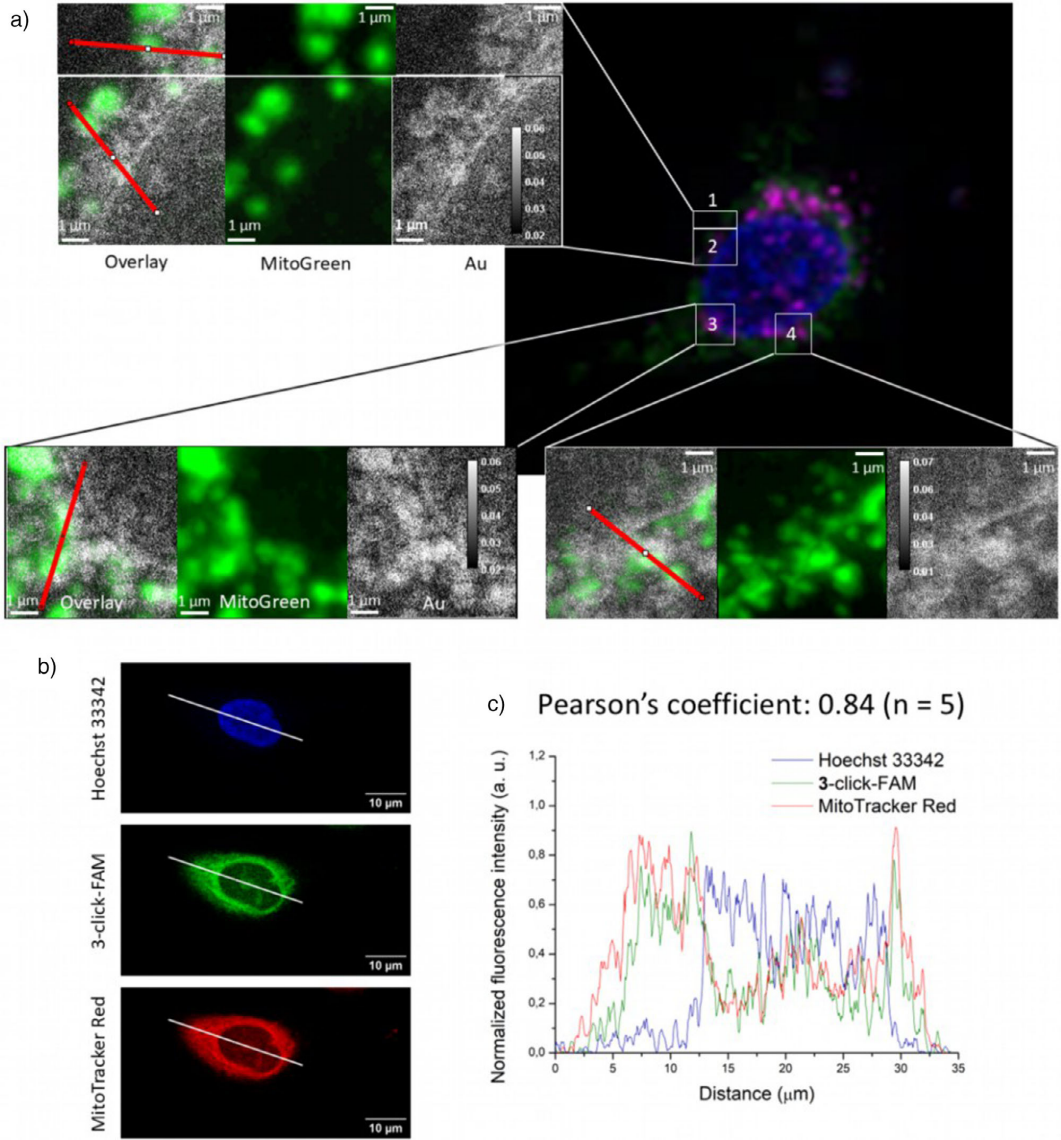
a) Correlative analysis of four regions of a frozen‐hydrated A549 cell treated with 1 µM of **3**‐**Br** for 4 h and imaged at 88 K. Light cryo‐microscopy (LM) of mitochondria (Mitotracker Green, MTR) and cryo‐synchrotron radiation X‐ray fluorescence imaging (cryo‐SR‐XRF) of elemental Au expressed in ng/mm^2^ as color‐ and gray‐scales, respectively. b) Confocal fluorescence microscopy images of A549 cells exposed to 1 µM of **3**‐**alkyne** for 4 h, fixed and clicked *in cellulo* with FAM‐N_3_ (green). Staining of DNA (Hoechst 33342, blue) and mitochondria (MitoTracker^TM^ Red, red). c) Intensity profiles of blue, green and red fluorescence along the line drawn on A549 cells treated with **3**‐**alkyne**.

To definitely ascertain the selective accumulation of complex **3** in the mitochondria and overcome bias on subcellular localization systematically observed when a fluorophore is pre‐attached to the metal complex under study,^[^ ^14^ ^]^ we set up a minimally invasive, in‐cell fluorescence tracking procedure based on a bioorthogonal reaction with a “clickable” derivative of **3**.^[^ ^17^, ^57^ ^−^ ^59^ ^]^ The derivative **3**‐**alkyne** (Figure 1) carrying an ethynyl handle was synthesized in 2 steps via the [(C^C)Au(dppv)]^+^ hydrothiolation pathway.^[^ ^41^ ^]^ The activity of complex **3**‐**alkyne** measured against A549 cells (EC_50_ = 0.15 ± 0.02 µM) appeared similar to those of **3** and **3**‐**Br**. Following previous experiments, A549 cells were incubated for 4 h with 1 µm of complex **3**‐**alkyne** and stained with Hoechst 33342 (nuclear marker) and MitoTracker™ Red (mitochondrial marker). In‐cell “click reaction” of fluorescein‐azide (FAM‐N_3_) was performed under optimized conditions followed by cellular imaging with a confocal fluorescence microscope. In these conditions, intense fluorescence was observed on the images taken in the green channel, while images of **3**‐**alkyne**‐, FAM‐N_3_‐ or ascorbate‐free controls showed very low green fluorescence signals (Figure S37), indicating selective labeling of complex **3**‐**alkyne** by FAM‐N_3_. Colocalization of the FAM probe and the mitochondria dye was visually apparent on the merged images taken in the green and red channels (Figure 5b; Figure S37). Quantification of the correlation between the green fluorescence signal of the FAM probe and with red fluorescence signal of MitoTracker™ Red using the JACoP plugin of Fiji software.^[^ ^60^ ^]^ led to a Pearson's coefficient of 0.84 (Figure 5b,c). Combined with cryo‐SR‐XRF data obtained with complex **3**‐**Br** (Figure 5a), this body of results strongly suggests a specific accumulation of complex **3** within the mitochondria.

In summary, despite differences in their structure and anticancer activity, the [(C^C)Au(P^P)]^+^ complexes **1–7** all demonstrated high stability in cell‐free conditions. By combining cryo‐SR‐XRF elemental nano‐imaging, cryo‐SR‐XAS, and HRMS, we ascertained the intracellular stability of the most active complex **3** inside lung cancer cells. Considering that the structurally related Pt(II) analog **8** displayed a similar chemical behavior and the same cytotoxic activity, this piece of work strongly suggests that the mode of action for this class of complexes involves the native [(C^C)Au(P^P)]^+^ cations that may disrupt specific biological targets via supramolecular interactions in contrast to [(C^C)Au(NHC)Cl] complexes which are suggested to trigger cell death by direct coordination of the gold center.^[^ ^61^ ^]^ The preferential accumulation of **3** in mitochondria, which we demonstrated by two independent imaging techniques, is fully consistent with its delocalized lipophilic cation nature and provides the missing piece of information to explain the antimitochondrial effects previously reported for structurally similar complexes.^[^ ^32^ ^]^ These results thus establish a clear relationship between the chemical structure and reactivity of a gold‐based compound, its speciation in a cellular setting, and its cytotoxicity.

## Supporting Information

The authors have cited additional references within the Supporting Information.

## Conflict of Interests

The authors declare no conflict of interest.

## Supporting information



Supporting Information

## Data Availability

The data that support the findings of this study are available in the supplementary material of this article.
