# Supplementary material for: Stability and Mitochondrial Localization of a Highly Cytotoxic Organogold(III) Complex with Diphosphine Ancillary Ligand in Lung Cancer Cells
Source: Angew Chem Int Ed Engl. 2025 Mar 27;64(22):e202422763. doi: 10.1002/anie.202422763 (PMC12105710; doi:10.1002/anie.202422763)
Supplement: Supplementary file 1 — Supporting Information [file ANIE-64-e202422763-s001.docx]

Supporting Information

for

Stability and Mitochondrial Localization of a Highly Cytotoxic Organogold(III) Complex with Diphosphine Ancillary Ligand in Lung Cancer Cells

Hester Blommaert,^1^ Clément Soep,^2^ Edwyn Remadna,^2^ Héloise Dossmann,^2^ Murielle Salomé,^3^ Olivier Proux,^4,5^ Isabelle Kieffer,^4,5^ Jean-Louis Hazemann,^4^* Sylvain Bohic,^6^* Michèle Salmain^2^* and Benoit Bertrand^2^*

^1^ Institut Néel, CNRS, Université Grenoble Alpes, 25 Avenue des Martyrs, 38042, Grenoble, France

^2^ Sorbonne Université, CNRS, Institut Parisien de Chimie Moléculaire, IPCM, F-75005 Paris, France.

Email: [benoit.bertrand@sorbonne-universite.fr](mailto:benoit.bertrand@sorbonne-universite.fr); michele.salmain@sorbonne-universite.fr

^3^ ESRF, The European Synchrotron Research Facility, F-38043 Grenoble cedex 9, France

^4^ BM16/FAME beamline, European Synchrotron Radiation Facility (ESRF), F-38000 Grenoble, France

Email: jean-louis.hazemann@neel.cnrs.fr

^5^ OSUG, UAR 832 Univ. Grenoble Alpes, CNRS, IRD, INRAE, Météo France, OSUG, 38000 Grenoble, France

^6^ Université Grenoble Alpes, INSERM, UA7 STROBE, Synchrotron Radiation for Biomedicine, F-38400 Saint Martin d’Hères, France

Email: sylvain.bohic@inserm.fr

| **Contents** | **Page** |
| --- | --- |
| **Experimental Section** | **S3-10** |
| **Figure S1**: Inhibition of A549 cell growth by complexes **1**–**8** | **S11** |
| **Table S1**: Effect of complexes **1**, **3**, **4** and **8** and the corresponding diphosphine ligands on lung cancer cells (A549), “triple negative” breast cancer cells (MDA-MB-231) and non-cancerous breast cells (MCF-10A) after 72 h incubation at 37 °C | **S11** |
| **Figure S2-6**: Representative curves of cell viability inhibition by complex **1**, **3**, **4** and **8** on A549, MDA-MB-231 and MCF10A cells. | **S12-13** |
| **Figure S7-14:** ESI-MS in positive mode of complex **1-8** alone or in solution with 1 eq. amino acids and/or guanine recorded after 24 h of incubation at 37 °C in DMSO. | **S14-21** |
| **Figure S15:** ^31^P{^1^H} NMR spectra (400MHz, 300 K, DMSO-d6) of complex **3** alone or in mixture with 1 eq. of amino acid recorded after 24 h of incubation at 37 °C in DMSO-d6. | **S22** |
| **Figure S16-23**: Reactivity of the cation of **1-8** with GSH (1, 2, 5, 10 and 50 equiv.) for 24 h at 37 °C. Samples were analyzed by ESI-MS. | **S23-30** |
| **Figure S24:** ^31^P{^1^H} NMR spectra (400MHz, 300 K, DMSO-d6) of complex **3** alone or in mixture with 1; 2; 5; 10 and 50 equivalents of NAC recorded after 24 h of incubation at 37 °C in DMSO-d6. | **S31** |
| **Figure S25-31**: Fragmentation by HCD of the [**1**-**2** and **4**-8-PF_6_]^+^ cations. | **S32-35** |
| **Table S2**: Biphenyl reductive elimination energies (E_0_) for Au(III) complexes **1**-**7** ± standard error and formula of the reductive elimination product characterized by in the HCD MS/MS spectra with less than 4 ppm. | **S36** |
| **Figure S32:** Cryo-SR-XRF elemental mapping of K, Zn, Au and Br in A549 cells treated with 1 µM of **3-Br** for 4 h and untreated controls. | **S36** |
| **Figure S33:** XANES spectrum of A549 cells treated with 1 µM of **3** for 4 h compared to spectrum of untreated A549 cells at the L_III_ edge of Au. Both spectra were recorded at 4 K in fluorescence mode. | **S37** |
| **Figure S34:** Estimation of the standard deviation of Linear Combination Fitting (LCF) results. | **S38** |
| **Table S3:** Linear combination fitting (LCF) results of **3** in A549 cells and **3** in DMEM culture medium. | **S38** |
| **Figure S35**: *Top:* HR-ESI-MS spectrum of cytosolic fraction of A549 cells lysate treated with 1 µM of complex **3** for 4 h. *Bottom*: Comparison between experimental isotopic pattern of signal at m/z = 859.28876 and calculated isotopic pattern of cation [**3**-PF_6_]^+^. | **S39** |
| **Figure S36**: Comparison of the normalized intensity of Au XRF and MitoTracker^TM^ Green signals along lines across images of regions 1-4 defined in figure 5A. | **S39** |
| **Figure S37:** Confocal fluorescence microscopy images of A549 cells exposed to 1 µM **3**-**alkyne** for 4 h, fixed and "clicked“ *in cellulo* with FAM-N_3_ (green) and control experiments without **3**-**alkyne**, FAM-N_3_ or ascorbate. Staining of DNA (Hoechst 33342, blue) and mitochondria (MitoTracker^TM^ Red, red). | **S40** |
| **Figure S38-45**: NMR spectra and HPLC chromatograms of complexes **7** and **8** | **S41-44** |
| **Optimized geometries for complexes 1-7 and their fragment** | **S45-55** |
| **References** | **S55-56** |

**Experimental Section**

**General remarks**

Anhydrous solvents were obtained by standard procedures. Chemicals were purchased from various manufacturers and used as received. ^1^H, ^13^C{^1^H} and ^31^P{^1^H} NMR spectra were acquired with Bruker 300 or 400 MHz spectrometers. Chemical shifts (δ) are expressed as ppm referenced to the solvent residual signal. Splitting patterns are expressed as follows: s, singlet; d, doublet; t, triplet; m, multiplet. HRMS analysis were carried out at the Mass Spectrometry Sciences Sorbonne University (MS^3^U) platform of Sorbonne Université (Paris). Elemental analyses were performed at the Service Chromato-Masse Microanalyse of the Université Paris-Saclay (Chatenay-Malabry, France). Complexes **1**-**5**, **8**, **3-Br** and **3-alkyne** and the reference complexes **Au1**-**4** were synthesized according to reported procedures.^[1–4]^

**Synthesis of complexes 6 and 7**

In a Schlenk tube under N_2_ atmosphere, the **gold dimer** (40 mg, 0.04 mmol) is suspended in degassed dichloromethane (4 mL). **The diphosphine** (0.08 mmol) was added and the reaction kept under N_2_ atmosphere at room temperature for 2 h. **Potassium hexafluorophosphate** (37 mg, 0.20 mmol) was dissolved in degassed acetonitrile (1 mL) and the reaction kept under N_2_ atmosphere at room temperature for 2 h. Dichloromethane (15 mL) was added into the mixture before filtration. The reaction mixture was filtered through a pad of Celite and the obtained solution was concentrated under reduced pressure. Upon addition of a large amount of Et_2_O, a pale yellow precipitate was formed which was recovered and gave after drying the pure product.

Complex: **(S-S)-Me-DUPHOS** (25 mg). The pure product **6** is obtained as a pale yellow powder (45 mg, 0.05 mmol, 61 % yield). ^1^H NMR (CDCl_3_, 300 MHz, 300 K): δ 8.09 (m, 2 H, H^8^), 7.97 (m, 2 H, H^9^), 7.72 (d, ^4^*J*_P-H_ = 10.3 Hz, 2 H, H^2^), 7.49 (dd, ^3^*J*_H-H_ = 7.6 Hz, ^5^*J*_P-H_ = 4.1 Hz, 2 H, H^5^), 7.35 (d, ^3^*J*_H-H_ = 7.8 Hz, 2 H, H^4^), 3.46 (m, 4 H, H^10^ + H^13^), 2.95-3.06 (dm, ^2^*J*_P-H_ = 29.4 Hz, 2 H, H^11/12^), 2.66-2.77 (dm, ^2^*J*_P-H_ = 27.4 Hz, 2 H, H^11/12^), 2.33 (m, 4 H, H^11/12^), 1.46 (d, ^3^*J*_H-H_ = 7.30 Hz, 3 H, H^14/15^), 1.40 (d, ^3^*J*_H-H_ = 7.30 Hz, 3 H, H^14/15^), 1.38 (s, 9 H, ^t^Bu), 0.95 (d, ^3^*J*_H-H_ = 7.30 Hz, 3 H, H^14/15^), 0.91 (d, ^3^*J*_H-H_ = 7.30 Hz, 3 H, H^14/15^). ^13^C{^1^H} Jmod NMR (CDCl_3_, 75.5 MHz, 300 K): δ 164.5 (dd, ^3^*J*_P-C_ = 114.9 Hz, ^3^*J*_P-C_ = 7.4 Hz, *C*^6^), 152.5 (d, ^2^*J*_P-C_ = 4.2 Hz, *C*^1^), 150.7 (dd, ^4^*J*_P-C_ = 9.3 Hz, ^4^*J*_P-C_ = 1.6 Hz, *C*^3^), 137.5 (dd, ^1^*J*_P-C_ = 45.2 Hz, ^2^*J*_P-C_ = 25.8 Hz, *C*^7^), 134.8 (m, *C*^8^ + *C*^9^), 134.2 (t, ^3^*J*_P-C_ = 6.3 Hz, *C*^2^), 125.8 (s, *C*^4^), 122.5 (d, ^4^*J*_P-C_ = 6.1 Hz, *C*^5^), 41.8 (d, ^1^*J*_P-C_ = 25.4 Hz, *C*^10/13^), 37.7 (d, ^1^*J*_P-C_ = 25.7 Hz, *C*^10/13^), 36.9 (d, ^2^*J*_P-C_ = 3.4 Hz, *C*^11/12^), 36.5 (s, *C*^11/12^), 35.7 (s, *C*_quat.tBu_), 31.6 (s, *C*H_3.tBu_), 17.3 (s, *C*^14/15^), 17.2 (s, *C*^14/15^), 14.4 (s, *C*^14/15^). ^31^P{^1^H} NMR (CDCl_3_, 121.5 MHz): δ 79.4 (s, 2 P, P-Au), -144.3 (h, ^1^*J*_P-F_ = 713 Hz, 1 P, PF_6_). ESI-MS (MeCN) *positive mode exact mass for* [C_38_H_52_P_2_Au]^+^ (767.3204): measured *m/z* 767.3206 [M-PF_6_]^+^. Calcd. for C_38_H_52_P_2_AuPF_6_ (912.713): C, 50.01; H, 5.74. Found: C, 49.85; H 5.95. Purity was determined to be > 97% by RP-HPLC: Rf = 4.65 min using the following method: flow rate: 1mL/min; λ = 254 nm; eluent A = MilliQ water with 0.1 % trifluoroacetic acid; eluent B = acetonitrile with 0.08 % trifluoroacetic acid; ratio A/B = 20/80; stationary phase: Nucleodur C18 HTec, 5 um, 4.6x150 mm (Macherey-Nagel).

Complex: **(R-R)-Me-DUPHOS** (25 mg). The pure product **7** is obtained as a pale yellow powder (48 mg, 0.053 mmol, 65 % yield). ^1^H NMR (CDCl_3_, 400 MHz, 300 K): δ 8.10 (m, 2 H, H^8^), 7.97 (m, 2 H, H^9^), 7.72 (d, ^4^*J*_P-H_ = 10.7 Hz, 2 H, H^2^), 7.49 (dd, ^3^*J*_H-H_ = 8.1 Hz, ^5^*J*_P-H_ = 4.1 Hz, 2 H, H^5^), 7.35 (d, ^3^*J*_H-H_ = 8.1 Hz, 2 H, H^4^), 3.46 (m, 4 H, H^10^ + H^13^), 2.94-3.07 (dm, ^3^*J*_P-H_ = 28.9 Hz, H^11/12^), 2.66-2.78 (dm, ^3^*J*_P-H_ = 28.9 Hz, H^11/12^), 2.34 (m, 4 H, H^11/12^), 1.45 (d, ^3^*J*_H-H_ = 7.2 Hz, H^14/15^), 1.40 (d, ^3^*J*_H-H_ = 7.2 Hz, H^14/15^), 1.38 (s, 18 H, ^t^Bu), 0.95 (d, ^3^*J*_H-H_ = 7.2 Hz, H^14/15^), 0.90 (d, ^3^*J*_H-H_ = 7.2 Hz, H^14/15^). ^13^C{^1^H} Jmod NMR (CDCl_3_, 75.5 MHz, 300 K): δ 164.5 (dd, ^3^*J*_P-C_ = 114.7 Hz, ^3^*J*_P-C_ = 6.9 Hz, *C*^6^), 152.6 (d, ^2^*J*_P-C_ = 3.5 Hz, *C*^1^), 150.8 (d, ^4^*J*_P-C_ = 9.6 Hz, *C*^1^), 137.5 (dd, ^1^*J*_P-C_ = 45.7 Hz, ^2^*J*_P-C_ = 25.8 Hz, *C*^7^), 134.9 (m, *C*^8^ + *C*^9^), 134.3 (t, ^3^*J*_P-C_ = 6.0 Hz, *C*^2^), 125.9 (s, *C*^4^), 125.5 (d, ^4^*J*_P-C_ = 6.5 Hz, *C*^5^), 41.8 (d, ^1^*J*_P-C_ = 26.1 Hz, *C*^10/13^), 37.8 (d, ^1^*J*_P-C_ = 26.0 Hz, *C*^10/13^), 37.0 (s, *C*^11/12^), 36.9 (s, *C*^11/12^), 36.6 (s, *C*^11/12^), 35.8 (s, *C*_quat.tBu_), 31.7 (s, *C*H_3.tBu_), 17.4 (s, *C*^14/15^), 17.3 (s, *C*^14/15^), 14.4 (s, *C*^14/15^). ^31^P{^1^H} NMR (CDCl_3_, 161.9 MHz): δ 79.5 (s, 2 P, P-Au), -144.3 (h, ^1^*J*_P-F_ = 711 Hz, 1 P, PF_6_). Calcd. for C_38_H_52_P_2_AuPF_6_ (912.713): C, 50.01; H, 5.74. Found: C, 49.69; H 6.07. Purity was determined to be > 97% by RP-HPLC: t_R_ = 4.60 min using the following method: flow rate: 1 mL/min; λ = 254 nm; eluent A = MilliQ water with 0.1 % trifluoroacetic acid; eluent B = acetonitrile with 0.08 % trifluoroacetic acid; ratio A/B = 20/80, stationary phase: Nucleodur C18 HTec, 5 um, 4.6x150 mm (Macherey-Nagel).

**Cell culture and cell growth inhibition**

Human lung adenocarcinoma cell line A549 (ATCC, CCL185) and human breast cancer cell line MDA-MB-231 (ATCC, HTB26) were cultivated in DMEM (Dulbecco’s Modified Eagle Medium) containing GlutaMax supplemented with 10% FBS (Fetal Bovine Serum) and at 37 °C in a humidified atmosphere and 5% CO_2_. Non-cancerous cell line MCF-10A (ATCC, HTB22) was maintained in DMEM:F12 (1:1) cell culture media, 5 % heat inactivated horse serum, supplemented with HEPES (20 mM), L-glutamine (2 mM), epidermal growth factor (20 ng/mL), hydrocortisone (500 ng/mL), cholera toxin (100 ng/mL), and insulin (10 μg/mL). Cell viability was evaluated by using a colorimetric method based on the tetrazolium salt [3-(4,5-dimethylthiazol-2-yl)-2,5-diphenyltetrazolium bromide] (MTT), which is reduced by viable cells to yield purple formazan crystals. Cells were seeded in 96-well plates at a density of 40000 cells/mL (100 μL per well). After overnight attachment, a dilution series of the compounds were added in the medium, and cells were incubated for a further 72 h. Stock solutions of the complexes were prepared in DMSO. The percentage of DMSO in the culture medium did not exceed 1%. After 72 h, the medium was removed, and the cells were incubated with MTT solution in PBS (10 μL of a 5 mg/mL solution) for 3 h. The formed purple formazan crystals were dissolved in 100 μL DMSO by thorough shaking, and the absorbance was measured at 585 nm using a microplate reader (FLUOstar OPTIMA, BMG Labtech). Each test was performed with at least 3 replicates and repeated at least 3 times. The IC_50_ value is determined using GraphPad Prism 8.0 software.

**Reactivity with biomolecules probed by mass spectrometry**

**Reaction with amino acids and guanine**: stock solutions of compounds **1**-**8** (20 mM in DMSO) and each amino acid (100 mM in DMSO) were prepared separately. Mixtures of the solution of compounds **1**-**8** with each amino acid or guanine were made: 250 μL of solution of **1**-**8**, 50 μL of solution of amino acid or guanine and 200 μL of DMSO. This led to solutions of 10 mM of **1**-**8** with one equivalent of amino acid and guanine. Solutions were incubated at 37 °C for 24 h and were diluted to 100 μM with MeOH.

**Reaction with glutathione**: two stock solutions of glutathione (GSH) were prepared: S_0_ at 200 mM in PBS (30.7 mg GSH dissolved in 500 μL PBS) and S_1_ at 20 mM (50 μL S_0_ + 450 μL PBS). The same solution of complexes **1**-**8** at 20 mM in DMSO were used. Following this, six solutions, with respectively 0, 1, 2, 5, 10 and 50 equivalents of glutathione and a constant concentration of **1**-**8** (1 mM in 9/1 PBS/DMSO mixture) in a total volume of solution of 100 μL were prepared. After a 24-h incubation at 37 °C, the solutions were diluted 100 times in methanol for analysis by mass spectrometry on a modified Quattro II mass spectrometer (Micromass, Manchester, U.K.) equipped with an electrospray (ESI) source. The sample solutions were infused into the ESI source at a flow rate of 400 μL·h^-1^. The following source parameters were applied: positive ion mode, ESI capillary voltage 3.50 kV, cone voltage 30 V. Nitrogen was used as the desolvatation and nebulizing gas. The source and desolvatation temperatures were kept at 100 °C. The data were acquired and analyzed using Masslynx software (version 4.2).

**Reactivity with biomolecules probed by ^31^P{^1^H} NMR spectroscopy**

**Reaction with amino acids**: stock solutions of compound **3** (20 mM in DMSO-d_6_) and each amino acid (20 mM in DMSO-d_6_) were prepared separately. Mixtures of compound **3** and each amino acid or guanine were made: 200 μL of solution of **3** + 200 μL of solution of amino acid or 200 µL of DMSO-d_6_ for control. This led to 10 mM solutions of **3** with one equivalent of amino acid. Solutions were incubated at 37 °C for 24 h and the ^31^P{^1^H} NMR were recorded on Bruker 400 MHz spectrometer and the spectra were analyzed with TopSpin 4.1.3 software.

**Reaction with N-acetyl cysteine**: two stock solutions of N-acetyl cysteine (NAC) were prepared: S_0_ at 200 mM in DMSO-d_6_ and S_1_ at 20 mM (50 μL S_0_ + 450 μL DMSO-d_6_). The same solution of complex **3** at 20 mM in DMSO-d_6_ was used. Following this, six solutions, with respectively 0, 1, 2, 5, 10 and 50 equivalents of NAC and a constant concentration of **3** (10 mM in DMSO-d_6_) in a total volume of solution of 400 μL were prepared. Solutions were incubated at 37 °C for 24 h and the ^31^P{^1^H} NMR were recorded on Bruker 400 MHz spectrometer and the spectra were analyzed with TopSpin 4.1.3 software.

**Stability of the complexes towards dissociation probed by mass spectrometry**

- **General information.** Mass spectrometry experiments were performed on a hybrid LTQ Orbitrap XL spectrometer (Thermo Fisher, San Jose, CA, USA). The complexes were introduced into an electrospray (ESI) source operated in positive mode, the electrospray voltage was set to 3.6 kV, the capillary voltage, to 10 V and the tube lens offset, to 40 V. The temperature of the drying gas was set at 275 °C and the flow rates of sheath, auxiliary and sweep gases (N_2_) were set to 8, 0 and 0, respectively (arbitrary units). Solutions were injected at a concentration of 1 mg/L into the ion source using a syringe pump at a flow rate of 5 μl/min. Activation and dissociation of the organometallic Au(III) complexes were performed by Higher-energy Collision Dissociation (HCD), the specific excitation mode of Orbitrap analyzers.^[5–7]^ In these experiments, precursor ions are isolated in the linear ion trap (LTQ) with an isolation window width of 5 u. Then, they are transmitted to the HCD cell (octopole) where they undergo a non-resonant activation by collisions on nitrogen for an activation time of 30 ms. Laboratory frame-of-reference energy (E_lab_) was then varied from 0 to 80 eV. The average number of microscans was set to 3, the automated gain control (AGC) target to 2x10^5^ charges and the maximum injection time to 500 ms. The fragment ions were then detected via the Orbitrap analyzer using a resolving power of 3 x 10^4^ at m/z 400. The survival yield (SY) curves as well as the ERMS (Energy-Resolved Mass Spectrometry) curves were plotted as a function of E_lab_.
- **Kinetic modeling.** To evaluate critical energies, a kinetic modeling was performed to simulate the experimental SY curves. This modeling was carried out with the *MassKinetics* ^[8]^ software (version 2.1.2.696) based on the RRKM (Rice-Ramsperger-Kassel-Marcus) theory ^[9–12]^ in order to access the E_0_ critical energies which correspond to the minimum energy required for dissociation at 0 K. To describe the internal energy deposited on an ion during HCD process, a model developed by our group and described elsewhere was used in this work.^[7]^ In this approach, the whole kinetic energy of the precursor ion is assumed to be converted into internal energy during the multiple collisions processes occurring in the HCD cell.

The following parameters were used for the modeling : vibrational frequencies of the precursor ion obtained at the M06/Def2-SVP level of theory on optimized geometries of the complexes using the Gaussian 16 software^[13]^ ; pre-exponential Arrhenius factor fixed at 10^15.5^ s^-1^, which is a fairly common value in the case of single bond breaking.^[14]^ Note that it was necessary to fix a reasonable value for the pre-exponential factor because this data cannot be experimentally accessed by HCD;^[5]^ decomposition time of 5 ms^[5,6]^

The uncertainties on the determined critical energies were estimated by varying different parameters used in the kinetic modelling. Initial internal energies <E_int_>_300K_ were varied from 280 to 320 K, the pre-exponential factor, from 10^14.2^ to 10^17.2^ s^-1^, decay time, from 2.5 to 10 ms, frequencies of the fundamental and transition states, by ± 10 %.

Collisional cross sections of the ions are requested in the modeling and were measured by ion mobility using a TimsTOF mass spectrometer (Bruker Daltonics, Bremen, Germany). In this case, solutions were injected at a flow rate of 5 μL/min into a positive-mode electrospray ionization source. The capillary voltage, end-plate offset and nebulizing gas pressure were set at 3.6 kV, 500 V and 8 psi, respectively. The drying gas temperature was set at 200 °C at a flow rate of 4 L/min. N_2_ was used as buffer gas for ion mobility measurements. Peak separations were performed in a range of inverse reduced mobility (1/K_0_) from 0.77 to 1.77 V.s/cm². Results are presented in Table S0.

**Table S0.** N_2_‐based collision cross sections (CCS) obtained for all complexes from ion mobility-MS measurements and internal energy calibration <E_int_> = slope x E_lab_ + <E_int_>_300K_ used for each studied complex.

| **Complex** | **CCS (Å²)** | **slope** | **<E_int_>_300K_ (eV)** |
| --- | --- | --- | --- |
| 1 | 277.3 | 0.1794 | 1.23 |
| 2 | 264.8 | 0.1801 | 1.20 |
| 3 | 266.0 | 0.1803 | 1.23 |
| 4 | 267.5 | 0.1802 | 1.25 |
| 5 | 269.0 | 0.1740 | 1.29 |
| 6 | 243.8 | 0.1807 | 1.13 |
| 7 | 243.8 | 0.1807 | 1.13 |

**Sample preparation for correlative light-XRF analysis**.

Graphene-coated silicon nitride membranes (Silson Ltd.) consisting of square silicon frames of 5 × 5 mm^2^ and 200 μm thickness with a central Si_3_N_4_ membrane of 1.5 × 1.5 mm^2^ and 500 nm thickness were used for cell culture and allow contaminant-free XRF low background signal. During manufacturing, a second, smaller (0.1 × 0.1mm^2^) membrane is added in one of the corners of the silicon frame to serve as the orientation object. Membranes were first exposed to UV irradiation for 30 min, thoroughly washed with PBS, and seeded with 4000−8000 A549 cells in a 10 μL drop of complete medium. The cells were incubated for 18 h at 37 °C and 5 % CO_2_ to adhere and spread on the substrates. They were incubated in a medium containing 5 % FBS + 1 μM **3-Br** for 4 h, then rinsed with PBS. They were further co-stained for 30 min in culture media with 100 nM Mitotracker Green FM and 5 μg/mL Hoechst 33342. Membranes were then washed with PBS and further samples were quickly rinsed with 150 mM ammonium acetate solution followed by manual blotting and plunge-freezing into liquid ethane chilled with liquid nitrogen.^[15]^

**Cryo-Fluorescence Light Microscopy (cryo-FLM).**

Plunge frozen cells were imaged using cryo-CLEM Thunder microscope system (Leica) equipped with a ceramic-tipped, 0.9 NA, 50× lens. The brightfield and bandpass filter cubes of GFP, DAPI, and Y5 were used. A complete mosaic of the1.5 × 1.5mm^2^ Si_3_N_4_ active area containing a vitrified cellular region of interest was registered with the collection for each field of view of a Z-stack projection over ∼20 μm. Images were acquired at 88 K and cryo-FLM image stacks processed using Leica's computational clearing and deconvolution package to remove out-of-focus blur, enhance contrast, and sharpen details. Further, a maximum intensity projection image is obtained and use to navigate on the SR-XRF end-station towards chosen regions of interest.

**SR-XRF Microscopy**.

The ID16A end-station is under high vacuum and is equipped with a cryostage to allow measurements of frozen-hydrated samples kept at 120 K and the cryotransfer is allowed through the combined usage of LEICA EM-VCM loading station, and EM-VCT vacuum cryo-shuttle systems. Nanopositioning is performed by a piezo-driven short-range hexapod stage regulated with the metrology of 12 capacitive sensors. All scanning uses “on-the-fly” acquisition with the sample translated at constant speed in the horizontal direction.^[16]^ The beam was focused to 26 × 42 nm^2^ (vertical × horizontal) using a pair of Kirkpatrick−Baez mirrors.^[17]^ The fluorescence signal emitted from each sample pixel was recorded by two custom multielement Silicon Drift Detectors (SDD) placed on both sides of the sample and facing each other at 90° from the incident X-ray beam. A multielement SDD (Hitachi Ltd.) and an ARDESIA-16 spectrometer based on monolithic SDD array^[18]^ were used. The resulting XRF spectra were fitted pixel by pixel using a dedicated Python script to correct for detector deadtime and perform normalization for variation of the incident X-ray beam. The elemental areal mass concentration was calculated using the Fundamental Parameters (FP) approach implemented in PyMca software package and a reference standard material containing elements of certified concentration (RF7-200-S2371 from AXO, Dresden, Germany) with uniform mass depositions in the range of ng/mm^2^ (1−3 atomic layers). The resulting elemental areal mass density maps were visualized with ImageJ software.

**Sample preparation for cryo-SR-XAS measurements**.

Solution samples. For the reference spectra, complexes **3** and **Au1-4** were dissolved at a concentration of 5 mM in dioxane. Complex **3** in dioxane was also added to a DMEM + 10% FBS medium to determine if complex **3** reacts with the cell culture medium. The final concentration of **3** was 0.5 mM in a 9(medium):1(dioxane)-solution. All solutions were transferred into the sample holder, frozen in liquid nitrogen and kept in liquid nitrogen until cryo-SR-XAS measurements.

Cell samples. Cells were grown in four T75 culture flasks in medium containing 5 % FBS until reaching around 80 % confluence. Cells were incubated with 1 µM of complex **3** for 4 h (DMSO content never exceeded 1 %). At the end of the incubation period, the complex containing medium was removed and the cells were rinsed twice with PBS and detached using 0.05 % trypsin solution (ThermoFisher Scientific). The detached cell suspension in the trypsin solution was diluted with 5 volumes of medium containing 5 % FBS and combined together. The cell suspension was centrifuged at 5000 rpm, 4 °C for 10 min and the supernatant was discarded. The cells were resuspended in 1 mL of PBS and centrifuged at 5000 rpm, 4 °C for 10 min and the supernatant was discarded. The obtained cell pellets were resuspended in 40 µL of cryopreservation medium (Gibco, ThermoFisher Scientific) and transferred into the sample holder before freezing with liquid nitrogen and kept in liquid nitrogen until cryo-SR-XAS measurements.

**Cryo-SR-XAS measurements.**

Au L_III_-edge HERFD-XAS measurements were conducted at the BM16 beamline of the ESRF synchrotron (Grenoble, France).^[19]^ The frozen-hydrated samples (references, cells and DMEM medium) were transferred to a cryostat at 10 K to minimize X-ray radiation damage and photo-reduction and to ensure a measurement in the native state of the cells. The measurements were carried out using a Si220 double crystal monochromator. An Au foil was simultaneously recorded in transmission mode for energy calibration of the XANES spectra. The energy was calibrated by setting the first derivative of the metallic foil reference spectrum to 11919.05 eV. Fluorescence signals were detected using a crystal analyser spectrometer (CAS). We used 11 Si(110) crystals and optimized them to select the Au-L_α1_ emission line. The energy bandwidth was estimated to be 0.9 eV by measuring the elastic peak at 9.7118 keV. During measurement, we collected 10 to 60 spectra per sample, depending on the Au concentration in the sample. Finally, the raw XANES spectra were averaged, energy-calibrated, and normalized for data interpretation.

Linear combination fitting (LCF)^[20]^ was performed with the Athena-Demeter program 0.9.26_._^[21]^ The normalized XANES spectra were fitted in the regions between -20 and 120 eV by linear combinations with the references recorded (**3** and **Au1-4**) (Fig. 4B). The R-factor (=∑[µ_exp_-µ_fit_]^2^/∑[µ_exp_]^2^) was used to assess the goodness of the fit. If the R-factor increased <5% compared to the best fit, the fits were considered equivalent. To estimate the uncertainty of the linear combination fitting with the level of noise of the experimental spectrum, we did an error analysis as done in the SI of ^[22]^. We created artificial multicomponent spectra from the set of references (**3** and **Au1-4**) (Fig. S34). The ratio of each species was determined randomly, but we constrained that the proportion of complex **3** was between 0.5 and 1 (because the results of the linear combination fits of **3** in A549 cells was in this range). To all multicomponent spectra, we added a noise comparable to the experimental spectrum of A549 cells incubated with **3**. Then we performed linear combination fitting on these artificial multicomponent spectra with the exact same method as for the experimental spectrum of complex **3** in A549 cells. The initial proportion of complex **3** was then compared to the LCF-calculated proportion of complex **3**) by linear regression. The average value of the residual (distance of datapoint from the linear that goes through the points (0,0 and (1, 1)) was then considered to be the standard deviation of the linear combination fitting procedure and was 2 %.

To further corroborate the experimental spectra of complex **3**, we performed XANES calculations with the FDMNES code.^[22]^ This code allows to model XANES spectra based on the structure of the molecule. From diffraction studies, structural input parameters were obtained of complex **3**. Different calculation cluster sizes around the central atom were used (3, 4, 5, and 6 Å) for the calculations. The best fit with the experimental spectra of complex **3** was with the calculation of 5 Å (Figure 4C). The spectra were convoluted considering an apparent core hole lifetime broadening of 2.03 eV.^[19]^

**HRMS study of A549 cytoplasmic fraction.**

A549 cells were seeded at a density of 5 × 10^5^ cells/well in a 6-well clear-bottom plate with a final volume of 2 mL of complete culture medium and allowed to adhere overnight. The culture medium was removed. Cells were incubated for 24 h in DMEM (Dulbecco’s Modified Eagle Medium) containing GlutaMax I supplemented with 10 % FBS with 1 μM of complex **3**. After 4 h incubation, the medium was removed. Cells were washed with PBS (5 mL), followed by mechanical detachment (scraper) in 1 mL cold PBS. Cells were centrifuged at 1200 rpm for 4 min to collect the pellet. After mechanical and chemical fractionation, the pellet was resuspended in a detergent-free lysis buffer composed of 1.938 mL of 25 mM Tris pH 7.5, 2 µL of 0.2 mM Na_3_VO_4_ solution in MilliQ water, 10 µL of 0.5 M solution of NaF in MilliQ water, 20 µL of 100 mM PMSF solution in EtOH, 20 µL of Phosphatase inhibitor cocktail Sigma P5276 and 10 µL of Proteinase inhibitor cocktail Sigma P2714 (total volume of 2 mL) for 40 min. The whole batch is gently crushed using a plunger, then centrifuged at 3000 rpm for 5 min. The cytoplasmic fraction was recovered and analyzed by high-resolution mass spectrometry. HR-MS spectra of cytoplasmic fraction were recorded on a Solarix XR FT-ICR mass spectrometer (Bruker Daltonics, Bremen, Germany) equipped with a 7T superconducting magnet and a dynamically harmonized ICR cell. The solutions were infused into the electrospray ionization (ESI) source with a 140 μL.h^-1^ flow rate. The following parameters were used for the ionization in the positive mode: capillary voltage: 4.5 kV; drying gas flow rate: 3 L.min^-1^; drying gas temperature: 250 °C; nebulizer gas pressure: 0.4 bar. All analyses were recorded in 40 scans with a quadrupole accumulation time of 0.5 s and a transient length of 0.8 s. Identification of the adducts was achieved by comparison with theoretical masses of the adducts using the Bruker Compass Data Analysis software (5.0 SR1).

**Confocal fluorescence imaging**.

60,000 A549 cells were seeded in an 8-well Ibidi µ-Slide® in 500 µL DMEM (Dulbecco’s Modified Eagle Medium) containing GlutaMax I supplemented with 10 % FBS for 24 h. The next day, the cells were treated for 4 h with **3**-**alkyne** at 1 μM in complete medium. Cells were stained with 500 ng.mL^-1^ Hoechst 33342 and 50 nM MitoTracker^TM^ Red in PBS for 30 min. Cells were fixed with 4 % PFA (Para-FormAldehyde) in PBS (500 µL/well) for 30 min at r. t. and washed three times with PBS (Phosphate Buffer Saline) containing 3% BSA (Bovine Serum Albumin). Cells were permeabilized using 0.1 % Triton X-100 in PBS for 30 min at r. t. Coverslips were washed with PBS containing 3% BSA. Click reaction was performed by incubation at r. t. of the cells in 500 µL PBS containing the homemade click cocktail (CuSO_4_.5H_2_O 5 mM + THPTA 25 mM), FAM-N_3_ (20 µM) and ascorbate (100 mM) during 2 h. Finally, the cells were washed three times with PBS containing 3% BSA. Coverslips were washed with PBS and preserved in Tris solution containing 1 % DABCO at pH 8.5 and kept at 4 °C in the dark until confocal microscopy imaging. Fluorescence images were acquired on the inverted LSM 710 Zeiss confocal equiped with a 63x oil immersion objective, NA 1,4. We used a 405 nm, 488 nm and 561 nm laser lines excitation for Hoechst33342 (blue), FAM (green), and MitoTracker^TM^ Red (red) staining respectively.


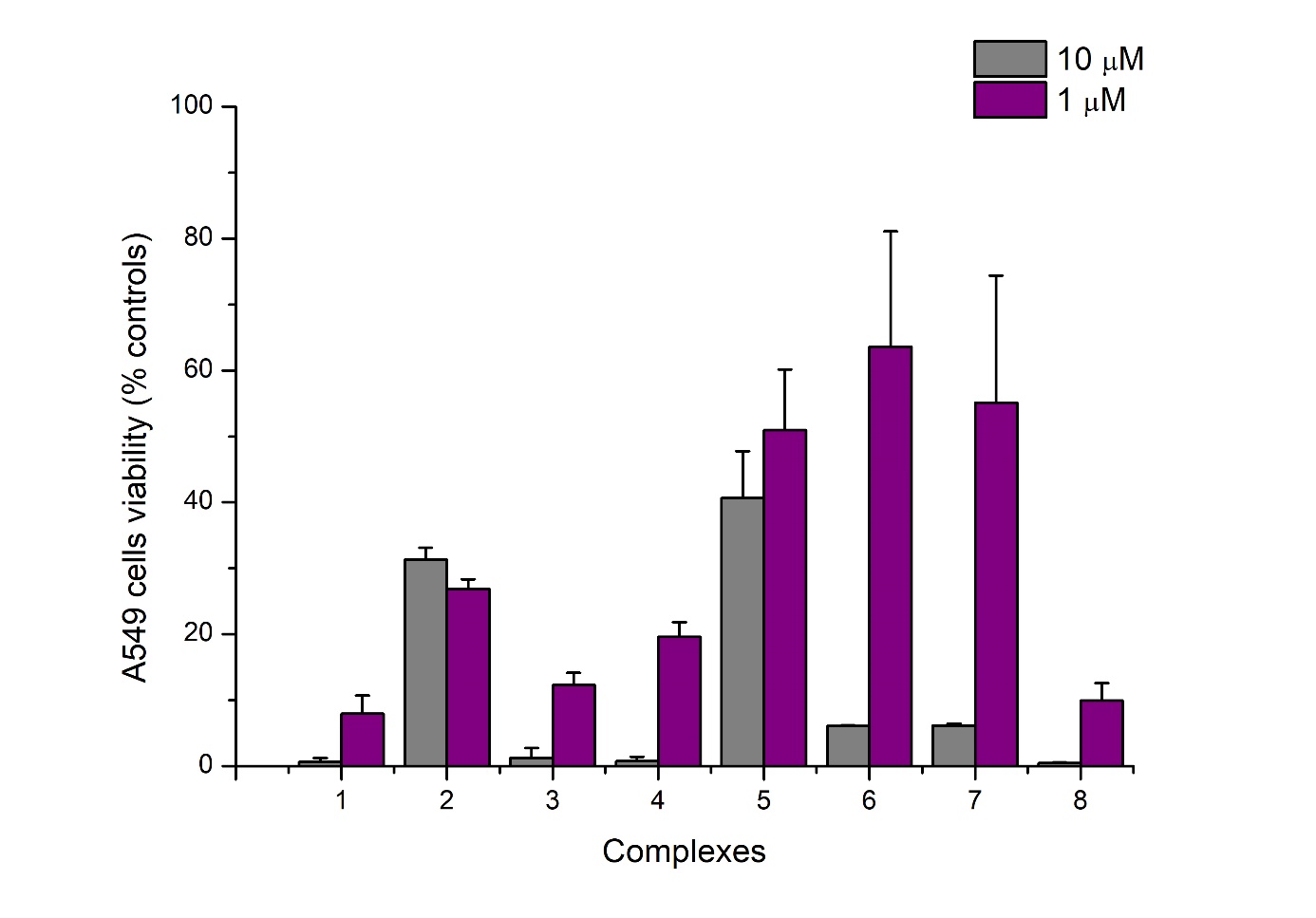


**Figure S1:** Inhibition of A549 cell growth by complexes **1**–**8**; each bar represents the average of three experiments ± standard error.

**Table S1**: Half-maximal effective concentration (EC_50_) of complexes **1**, **3**, **4**, **8**, cisplatin and corresponding diphosphine ligands on lung cancer cells (A549), “triple negative” breast cancer cells (MDA-MB-231) and non-cancerous breast epithelial cells (MCF-10A) after 72 h incubation at 37 °C and half-maximal effective concentration (EC_50_) of complex **3** and 1,2-diphenylphosphinoethane on lung cancer cells (A549) after 72 h incubation at 37 °C; each data represents the average of three experiments ± standard error.

| Complex | EC_50_ 72 h (µM) | | | EC_50_ 4 h (µM) |
| --- | --- | --- | --- | --- |
|  | A549 | MDA-MB-231 | MCF-10A | A549 |
| **1** | 0.07 ± 0.02 | 0.07 ± 0.01 | 0.11 ± 0.01 | - |
| **3** | 0.05 ± 0.01 | 0.08 ± 0.01 | 0.18 ± 0.01 | 1.2 ± 0.1 |
| **4** | 0.07 ± 0.02 | 0.12 ± 0.01 | 1.00 ± 0.01 | - |
| **8** | 0.04 ± 0.01 | 0.09 ± 0.02 | 0.09 ± 0.01 | - |
| **Cisplatin^a^** | 1.7 ± 0.5 | 20.4 ± 3.4 | 3.2 ± 0.8 | - |
| **1,2-diphenylphosphinobenzene** | 0.46 ± 0.05 | - | - | - |
| **1,2-diphenylphosphinoethane** | 0.35 ± 0.08 | - | - | > 50 |
| **1,3-diphenylphosphinopropane** | 0.32 ± 0.02 | - | - | - |

^a^Ref. [23,24]


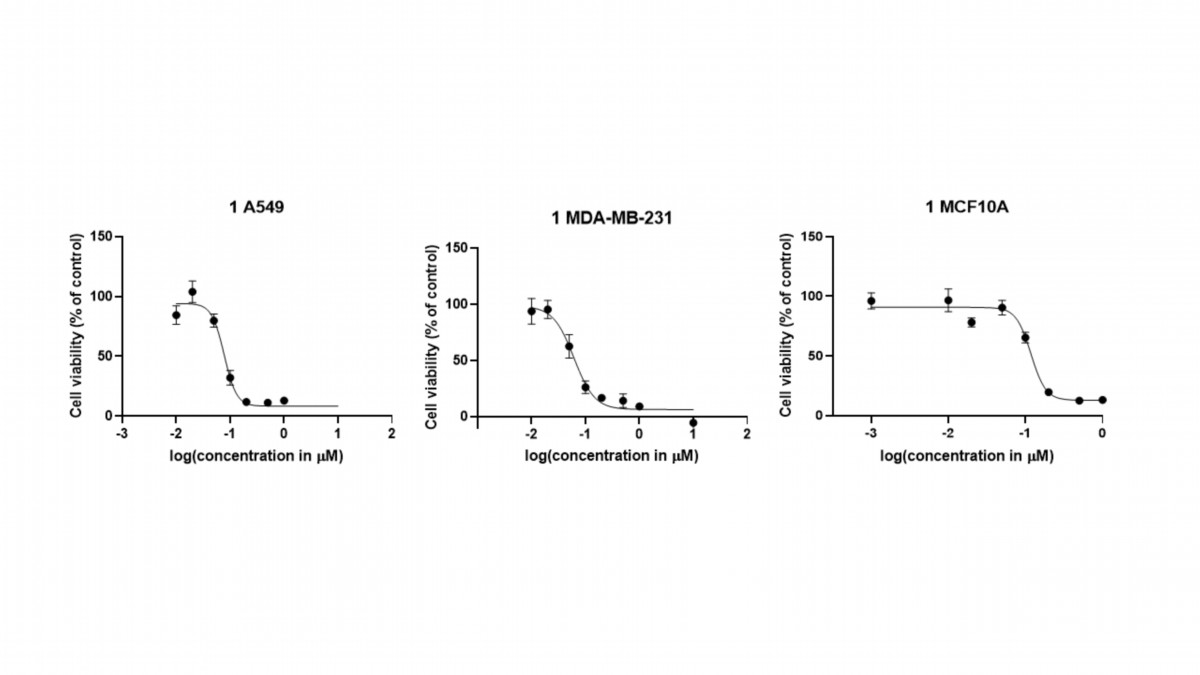


**Figure S2**: Representative curves of cell viability inhibition by complex **1** on A549, MDA-MB-231 and MCF-10A cells after 72 h of incubation at 37 °C.


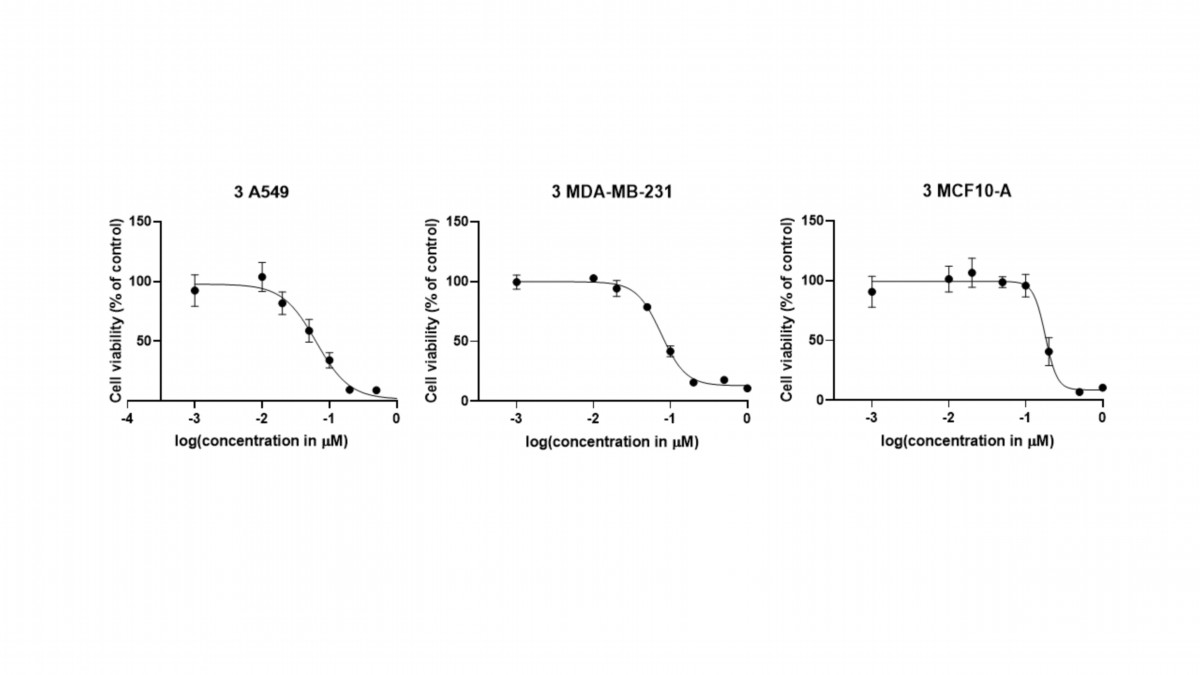


**Figure S3**: Representative curves of cell viability inhibition by complex **3** on A549, MDA-MB-231 and MCF-10A cells after 72 h of incubation at 37 °C.


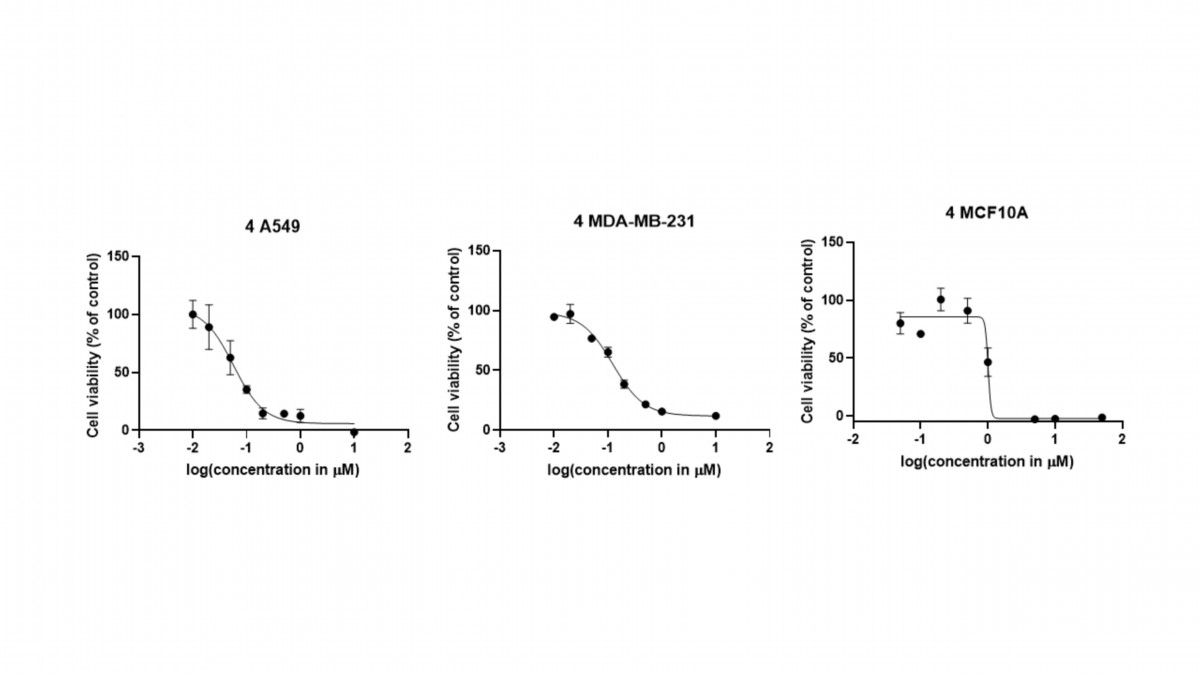


**Figure S4**: Representative curves of cell viability inhibition by complex **4** on A549, MDA-MB-231 and MCF-10A cells after 72 h of incubation at 37 °C.


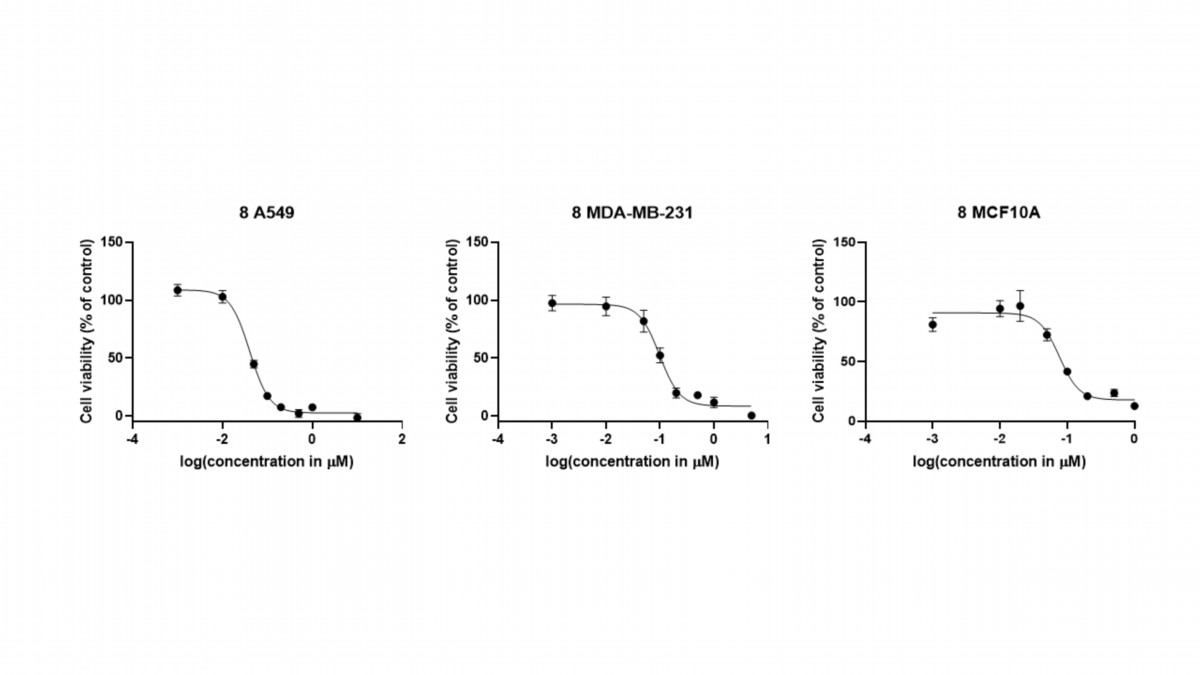


**Figure S5**: Representative curves of cell viability inhibition by complex **8** on A549, MDA-MB-231 and MCF-10A cells after 72 h of incubation at 37 °C.

**Figure S6**: Representative curves of cell viability inhibition by complex **3** and 1,2-diphenylphosphinoethane (dppe) on A549 cells after 4 h of incubation at 37 °C.


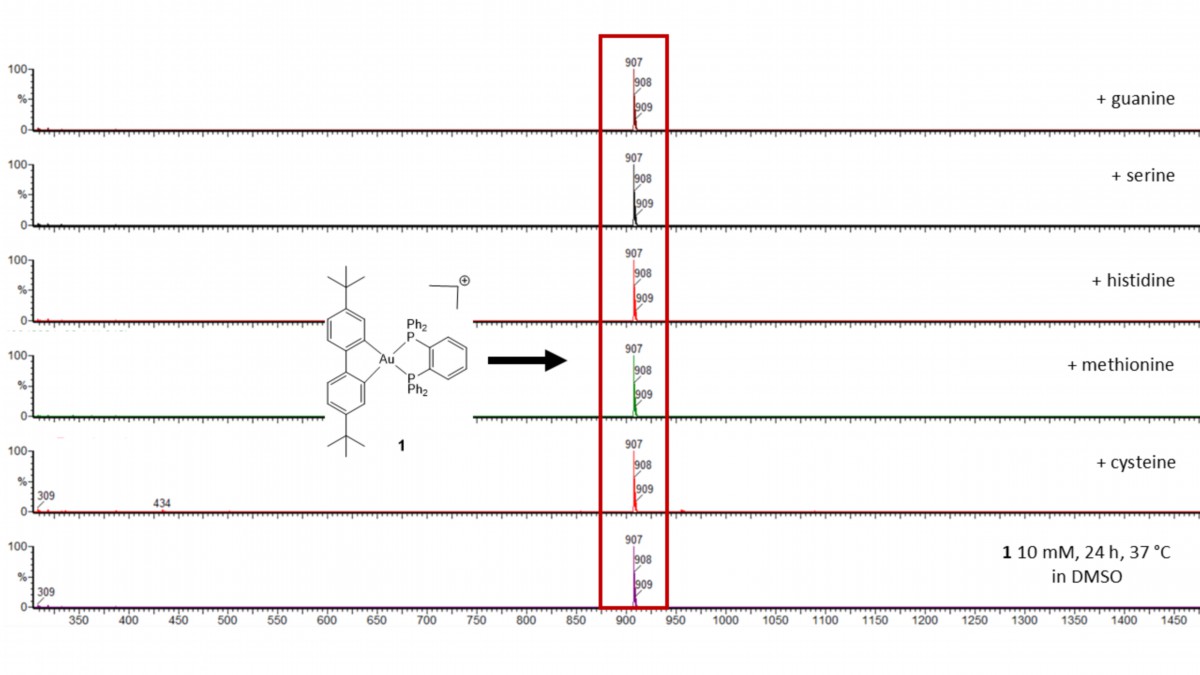


**Figure S7:** Electrospray ionization-mass spectrometry (ESI-MS) in positive mode of complex **1** alone or in mixture with 1 eq. amino acid or guanine recorded after 24 h of incubation at 37 °C in DMSO.


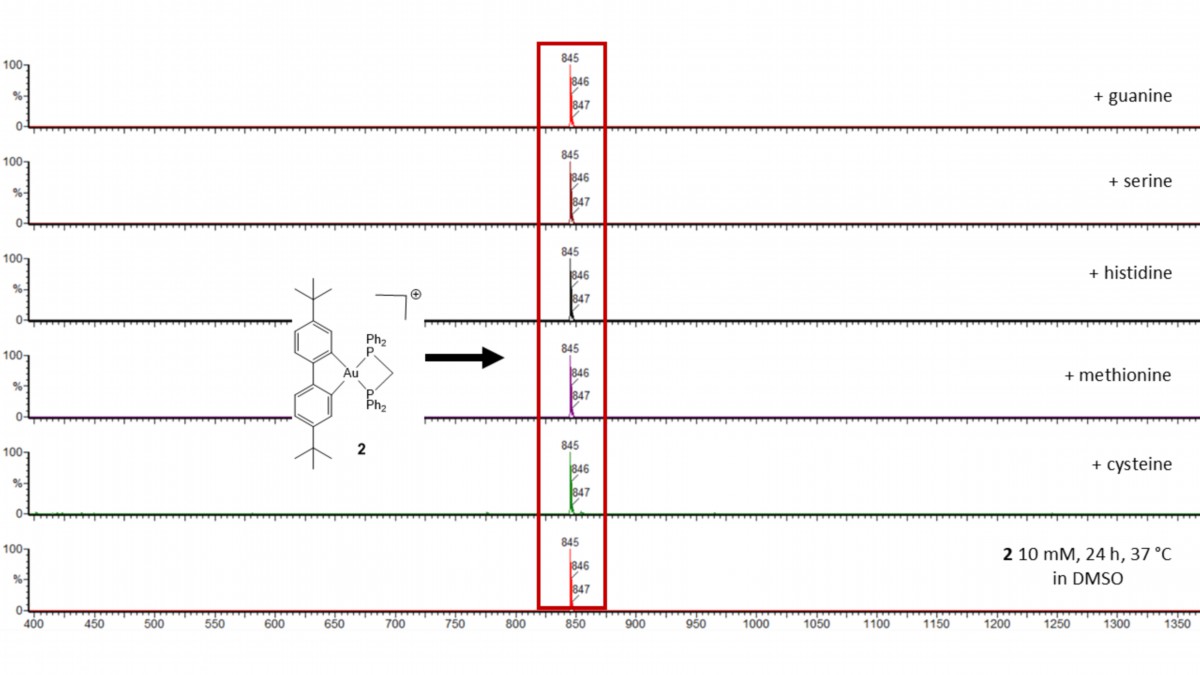


**Figure S8:** ESI-MS in positive mode of complex **2** alone or in mixture with 1 eq. amino acid or guanine recorded after 24 h of incubation at 37 °C in DMSO.


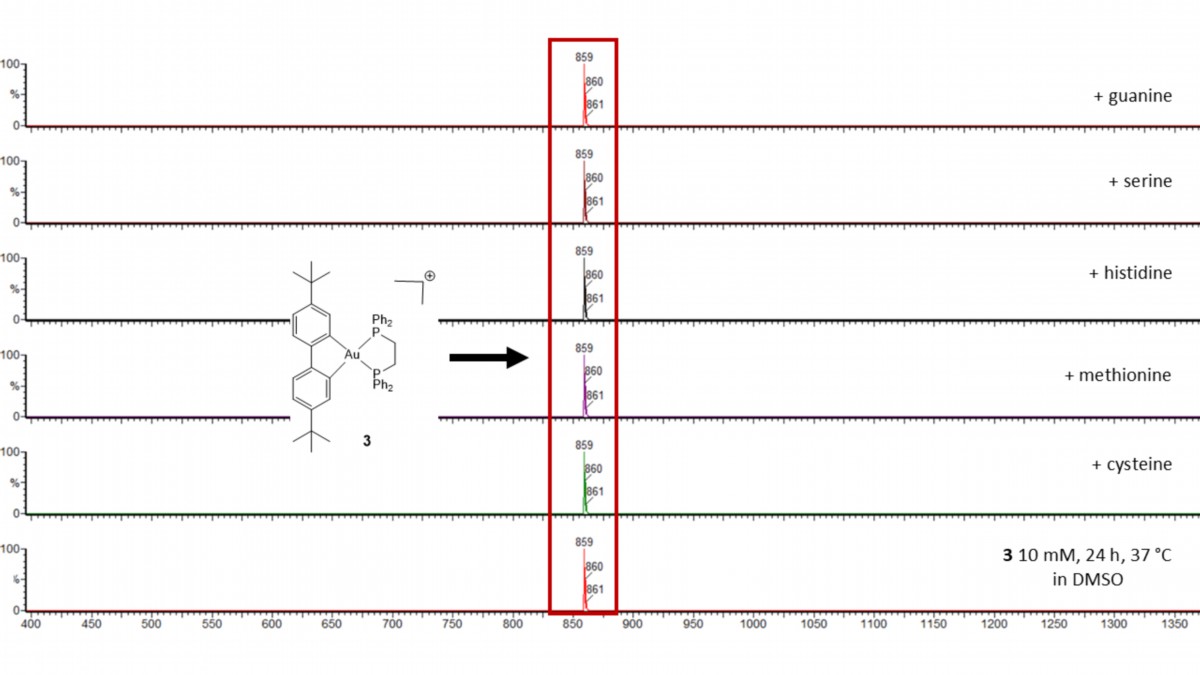


**Figure S9:** ESI-MS in positive mode of complex **3** alone or in mixture with 1 eq. amino acid or guanine recorded after 24 h of incubation at 37 °C in DMSO.


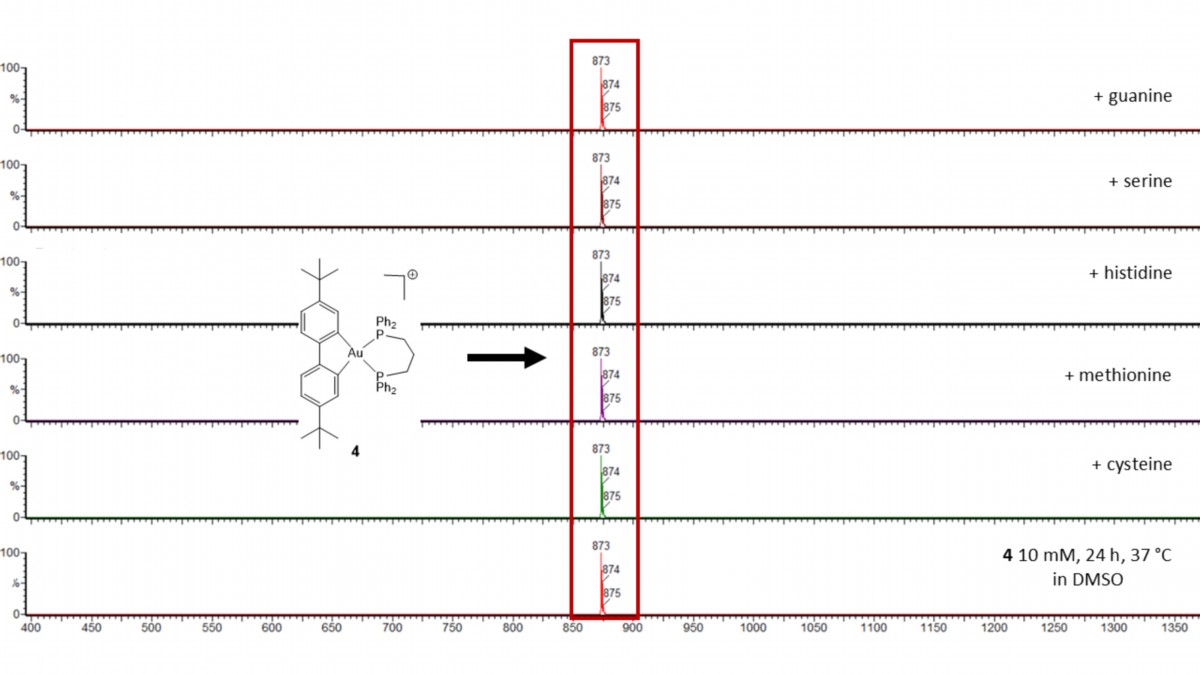


**Figure S10:** ESI-MS in positive mode of complex **4** alone or in mixture with 1 eq. amino acid or guanine recorded after 24 h of incubation at 37 °C in DMSO.


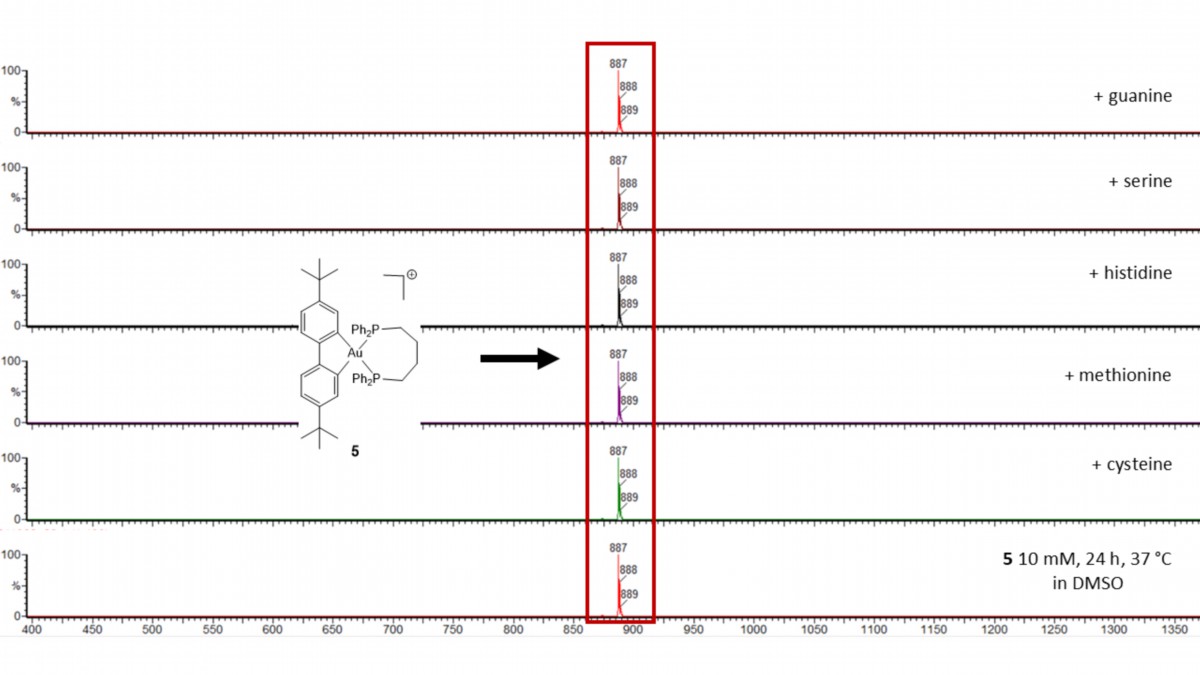


**Figure S11:** ESI-MS in positive mode of complex **5** alone or in mixture with 1 eq. amino acid or guanine recorded after 24 h of incubation at 37 °C in DMSO.


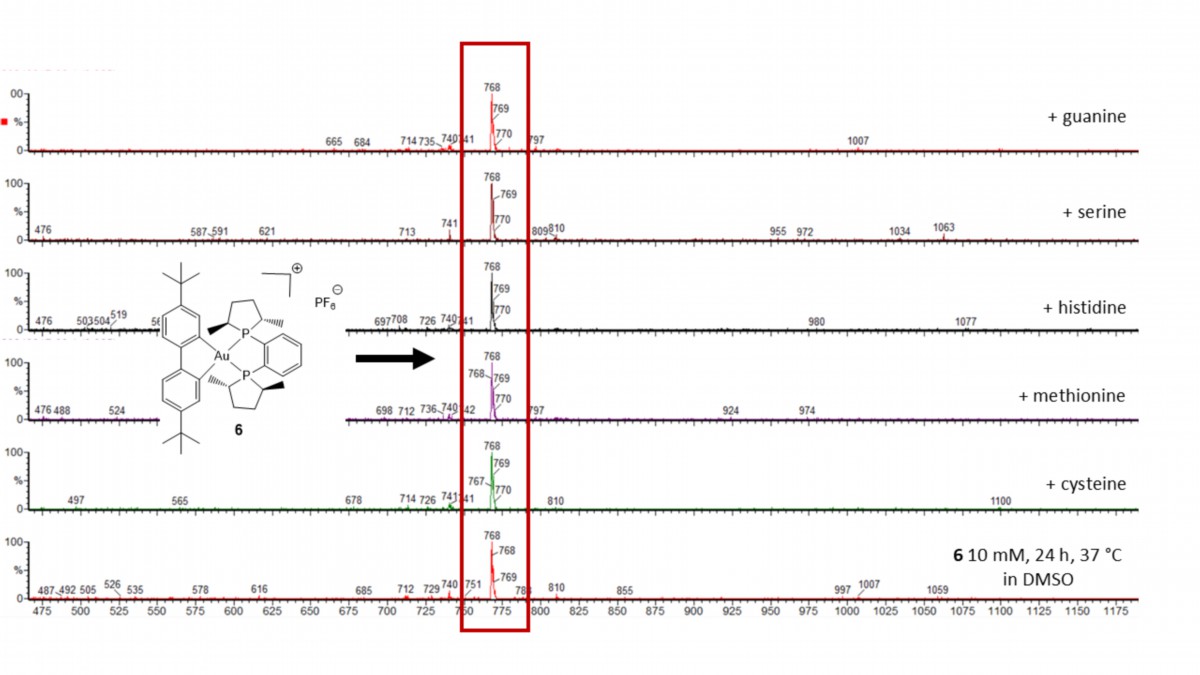


**Figure S12:** ESI-MS in positive mode of complex **6** alone or in mixture with 1 eq. amino acid or guanine recorded after 24 h of incubation at 37 °C in DMSO.


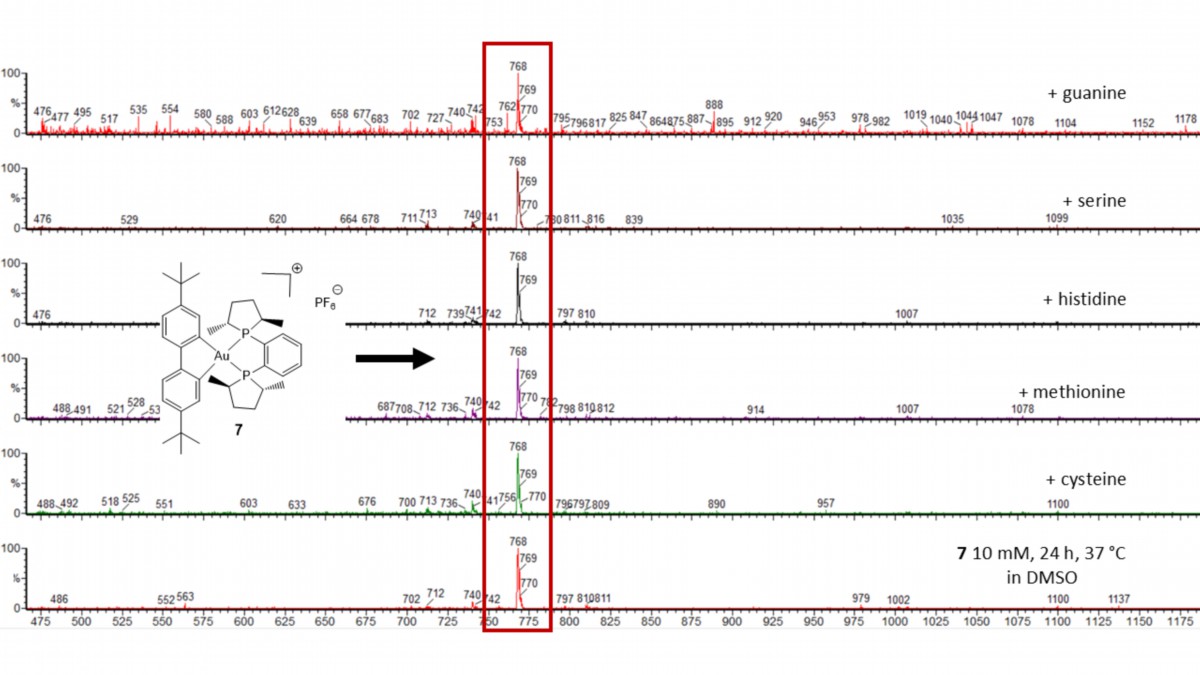


**Figure S13:** ESI-MS in positive mode of complex **7** alone or in mixture with 1 eq. amino acid or guanine recorded after 24 h of incubation at 37 °C in DMSO.


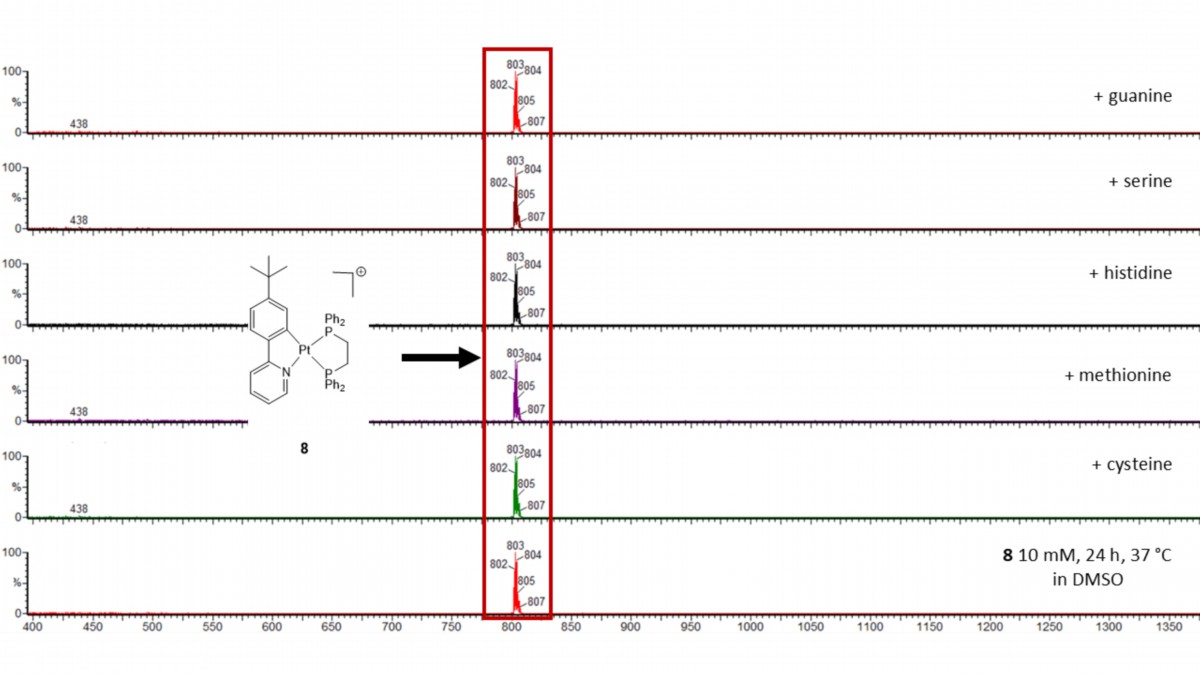


**Figure S14:** ESI-MS in positive mode of complex **8** alone or in mixture with 1 eq. amino acid or guanine recorded after 24 h of incubation at 37 °C in DMSO.


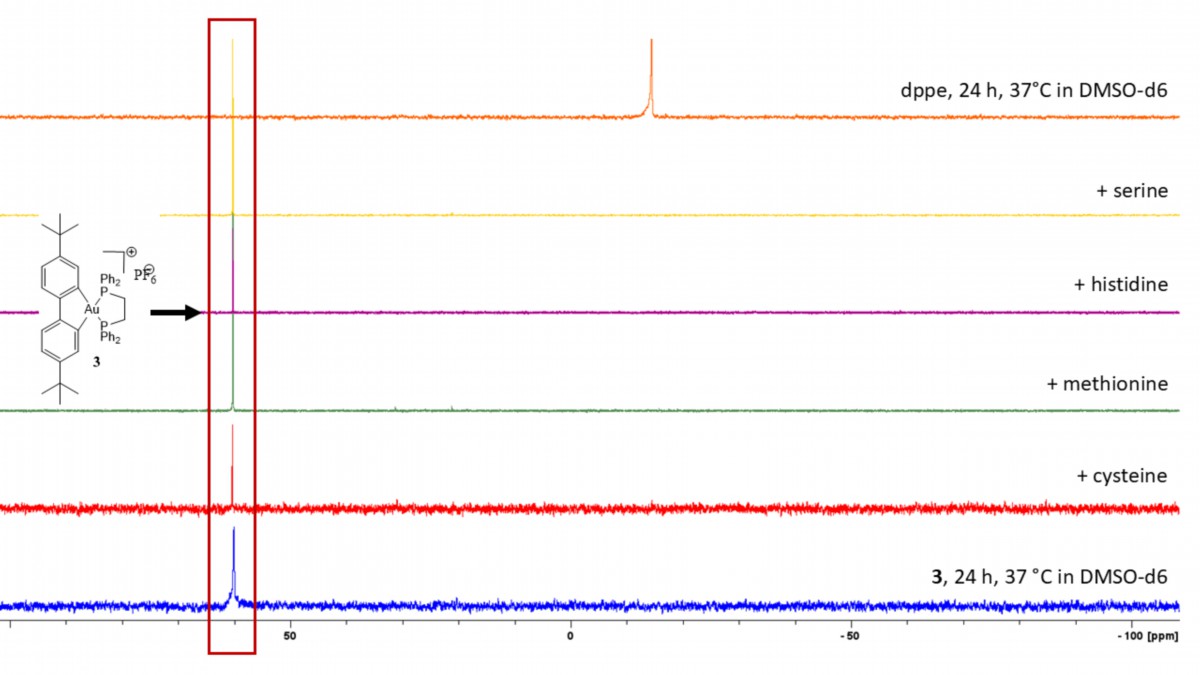


**Figure S15:** ^31^P{^1^H} NMR spectra (400 MHz, 300 K, DMSO-d_6_) of complex **3** alone or in mixture with 1 eq. of amino acid recorded after 24 h of incubation at 37 °C in DMSO-d_6_.


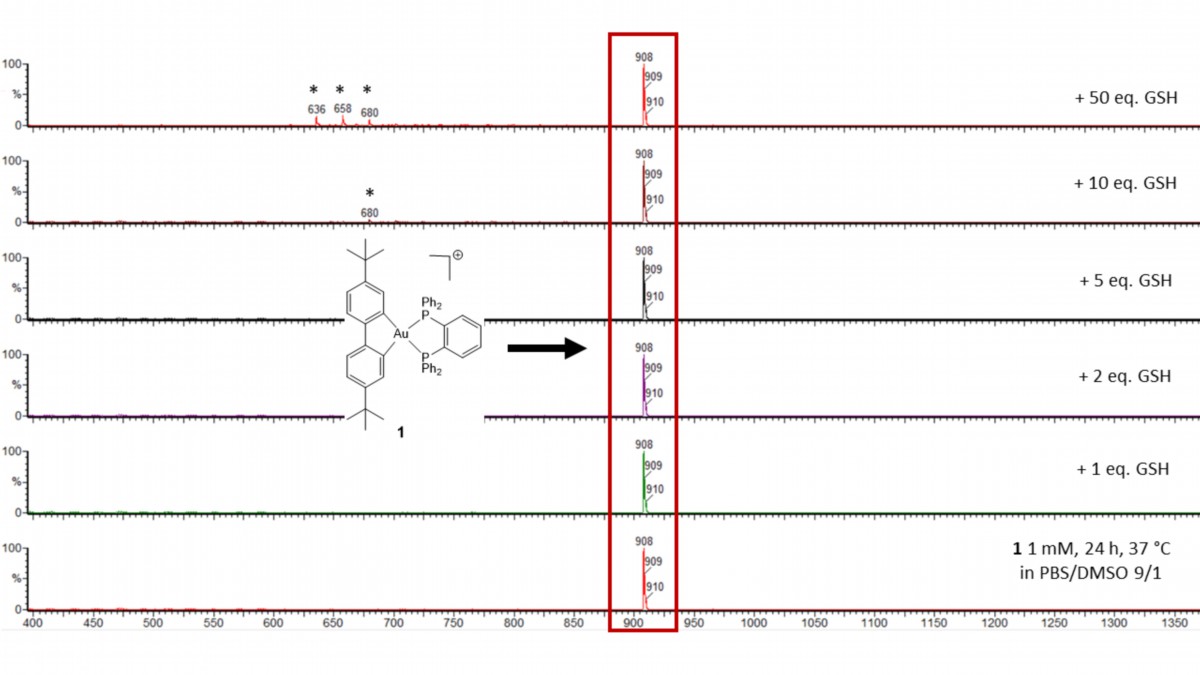


**Figure S16**: Reaction of the cation of **1** with GSH (1, 2, 5, 10 and 50 equiv.) for 24 h at 37 °C. Samples were analyzed by ESI-MS. The ions marked with a star on the spectra are attributed to [GSSG + Na]^+^ (m/z 635.1), [GSSG-H + 2 Na]^+^ (m/z 657.1) and [GSSG-2H + 3 Na]^+^ (m/z 679.1).


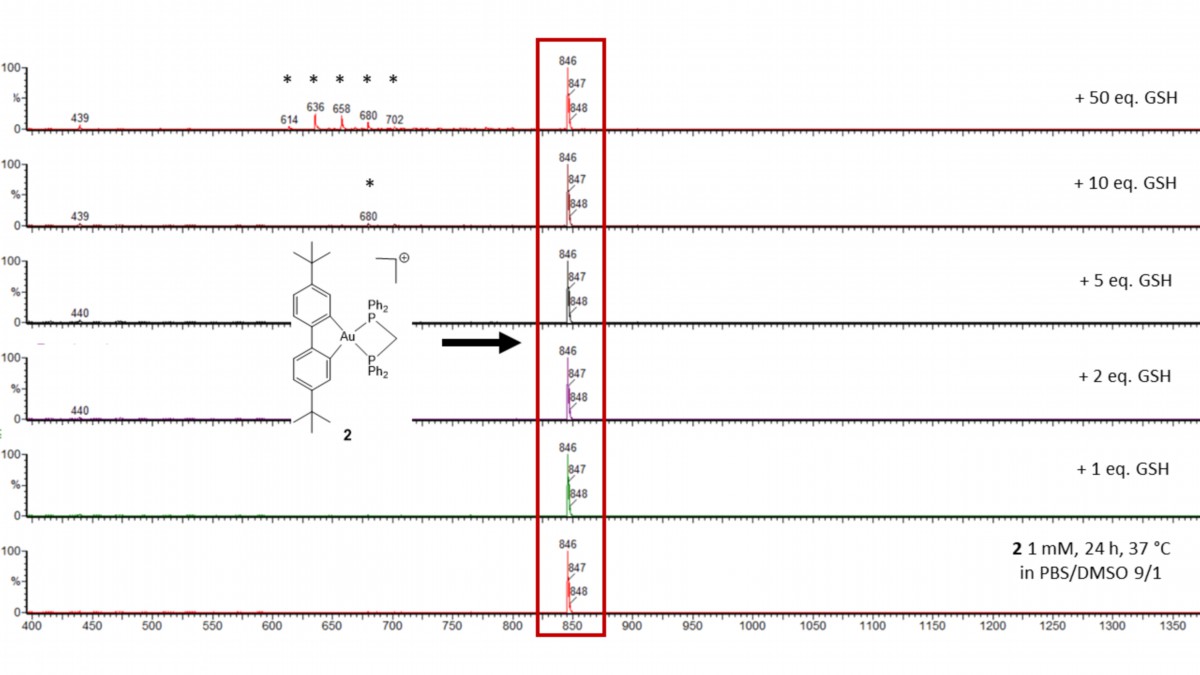


**Figure S17**: Reaction of the cation of **2** with GSH (1, 2, 5, 10 and 50 equiv.) for 24 h at 37 °C. Samples were analyzed by ESI-MS. The ions marked with a star on the spectra are attributed to [GSSG + H]^+^ (m/z 613.2), [GSSG + Na]^+^ (m/z 635.1), [GSSG-H + 2 Na]^+^ (m/z 657.1), [GSSG-2H + 3 Na]^+^ (m/z 679.1) and [GSSG-3H + 4Na]^+^ (m/z 701.1).


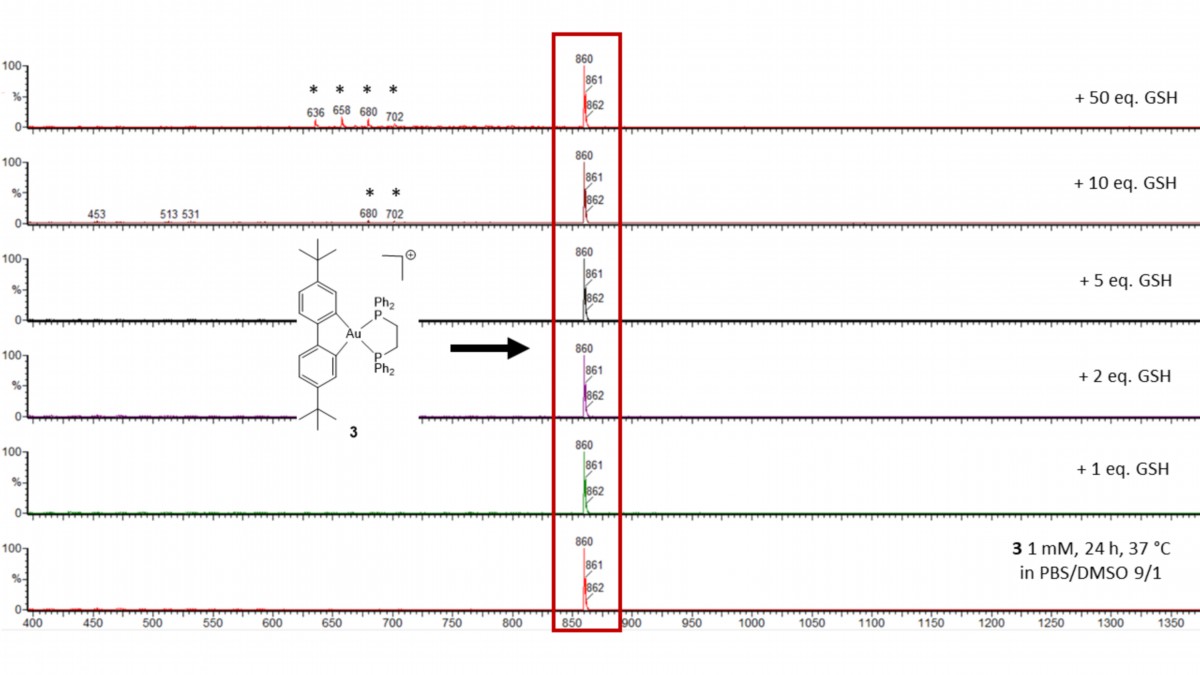


**Figure S18**: Reaction of the cation of **3** with GSH (1, 2, 5, 10 and 50 equiv.) for 24 h at 37 °C. Samples were analyzed by ESI-MS. The ions marked with a star on the spectra are attributed to [GSSG + Na]^+^ (m/z 635.1), [GSSG-H + 2 Na]^+^ (m/z 657.1), [GSSG-2H + 3 Na]^+^ (m/z 679.1) and [GSSG-3H + 4Na]^+^ (m/z 701.1).


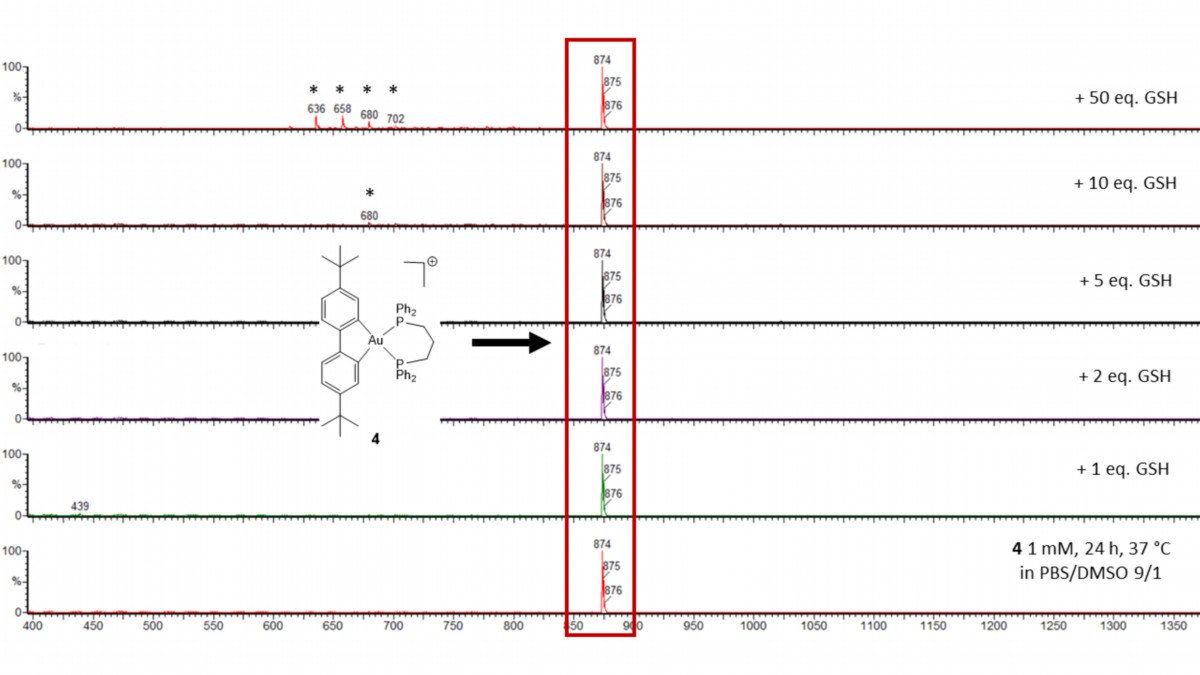


**Figure S19**: Reaction of the cation of **4** with GSH (1, 2, 5, 10 and 50 equiv.) for 24 h at 37 °C. Samples were analyzed by ESI-MS. The ions marked with a star on the spectra are attributed to [GSSG + Na]^+^ (m/z 635.1), [GSSG-H + 2 Na]^+^ (m/z 657.1), [GSSG-2H + 3 Na]^+^ (m/z 679.1) and [GSSG-3H + 4Na]^+^ (m/z 701.1).


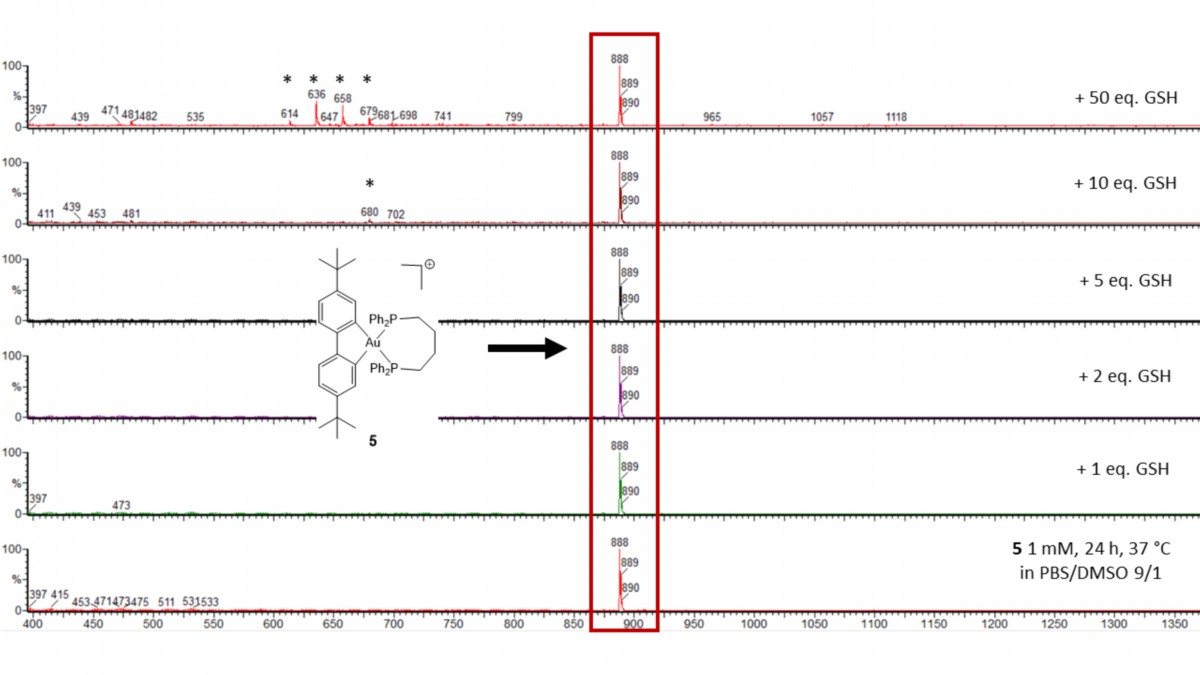


**Figure S20**: Reaction of the cation of **5** with GSH (1, 2, 5, 10 and 50 equiv.) for 24 h at 37 °C. Samples were analyzed by ESI-MS. The ions marked with a star on the spectra are attributed to [GSSG + H]^+^ (m/z 613.2), [GSSG + Na]^+^ (m/z 635.1), [GSSG-H + 2 Na]^+^ (m/z 657.1) and [GSSG-2H + 3 Na]^+^ (m/z 679.1).


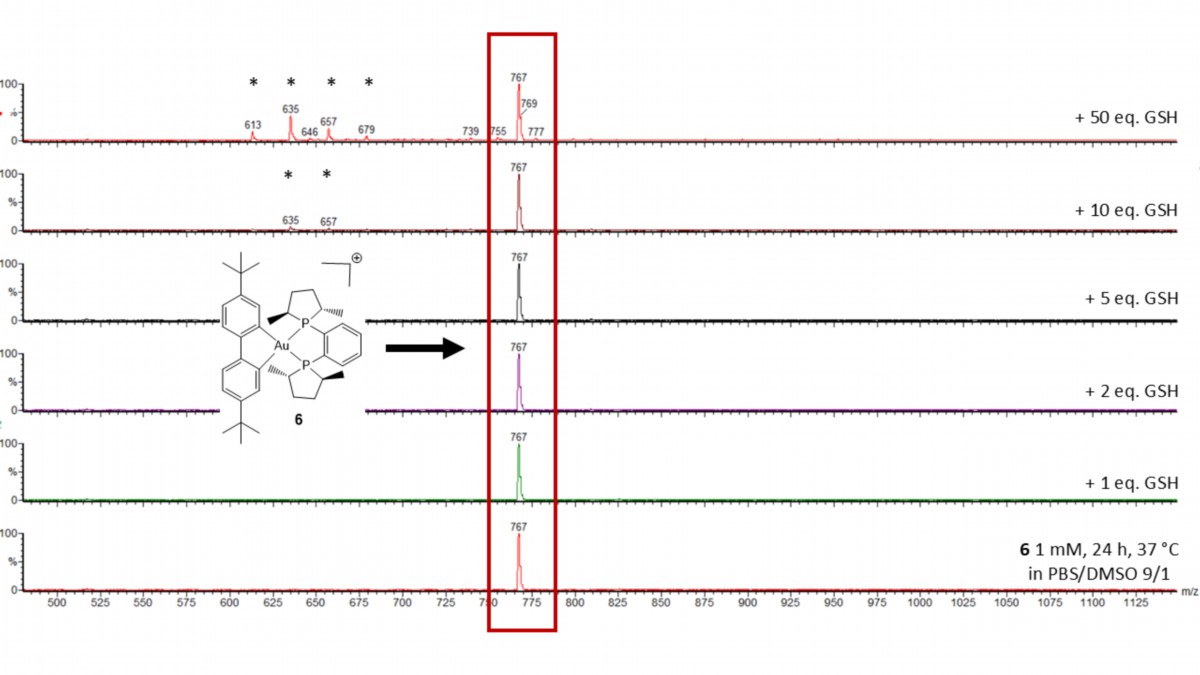


**Figure S21**: Reaction of the cation of **6** with GSH (1, 2, 5, 10 and 50 equiv.) for 24 h at 37 °C. Samples were analyzed by ESI-MS. The ions marked with a star on the spectra are attributed to [GSSG + H]^+^ (m/z 613.2), [GSSG + Na]^+^ (m/z 635.1), [GSSG-H + 2 Na]^+^ (m/z 657.1) and [GSSG-2H + 3 Na]^+^ (m/z 679.1).


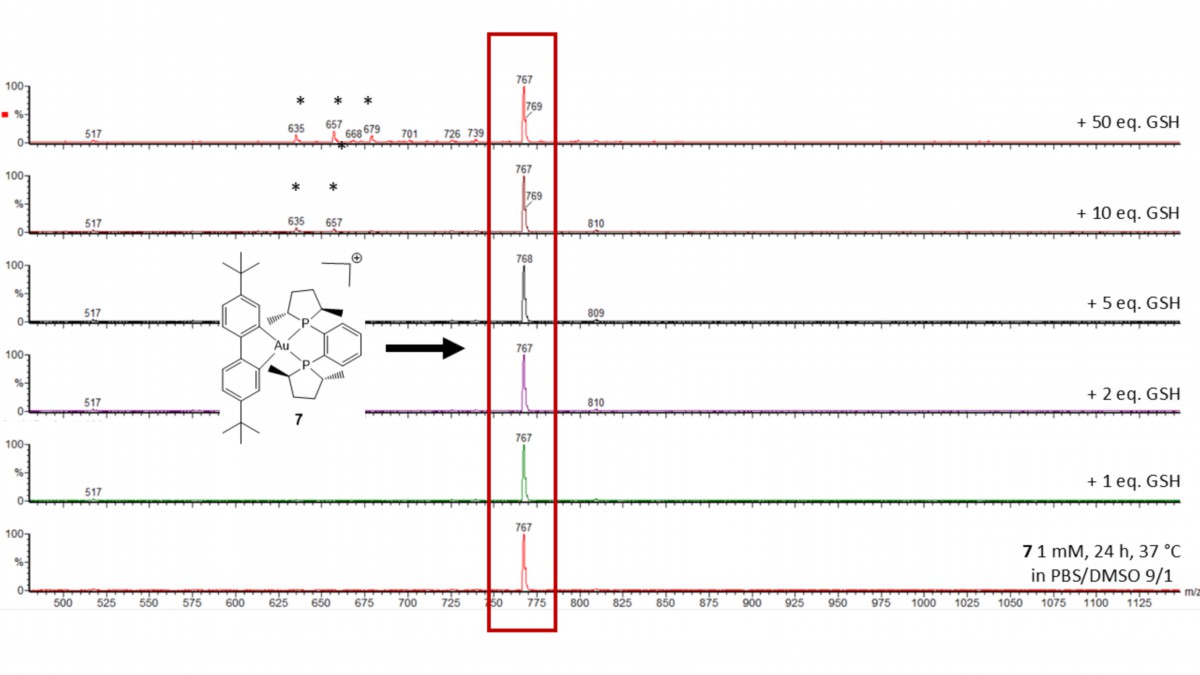


**Figure S22**: Reaction of the cation of **7** with GSH (1, 2, 5, 10 and 50 equiv.) for 24 h at 37 °C. Samples were analyzed by ESI-MS. The ions marked with a star on the spectra are attributed to [GSSG + Na]^+^ (m/z 635.1), [GSSG-H + 2 Na]^+^ (m/z 657.1) and [GSSG-2H + 3 Na]^+^ (m/z 679.1).


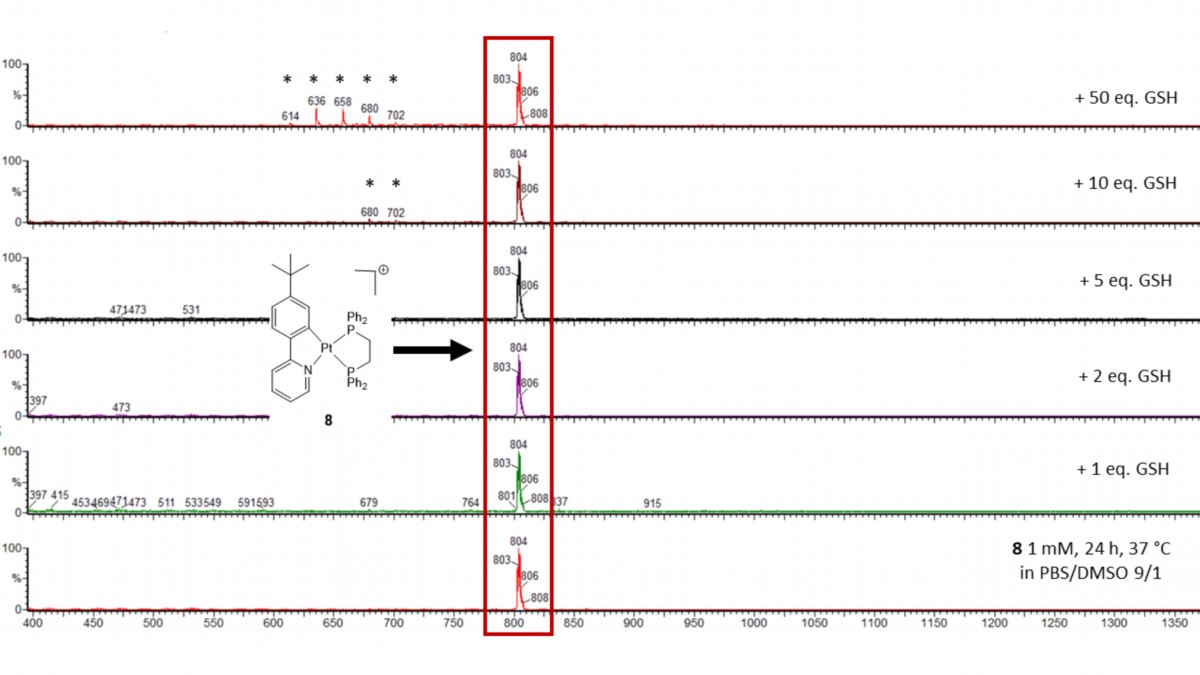


**Figure S23**: Reaction of the cation of **8** with GSH (1, 2, 5, 10 and 50 equiv.) for 24 h at 37 °C. Samples were analyzed by ESI-MS. The ions marked with a star on the spectra are attributed to [GSSG + H]^+^ (m/z 613.2), [GSSG + Na]^+^ (m/z 635.1), [GSSG-H + 2 Na]^+^ (m/z 657.1), [GSSG-2H + 3 Na]^+^ (m/z 679.1) and [GSSG-3H + 4Na]^+^ (m/z 701.1).


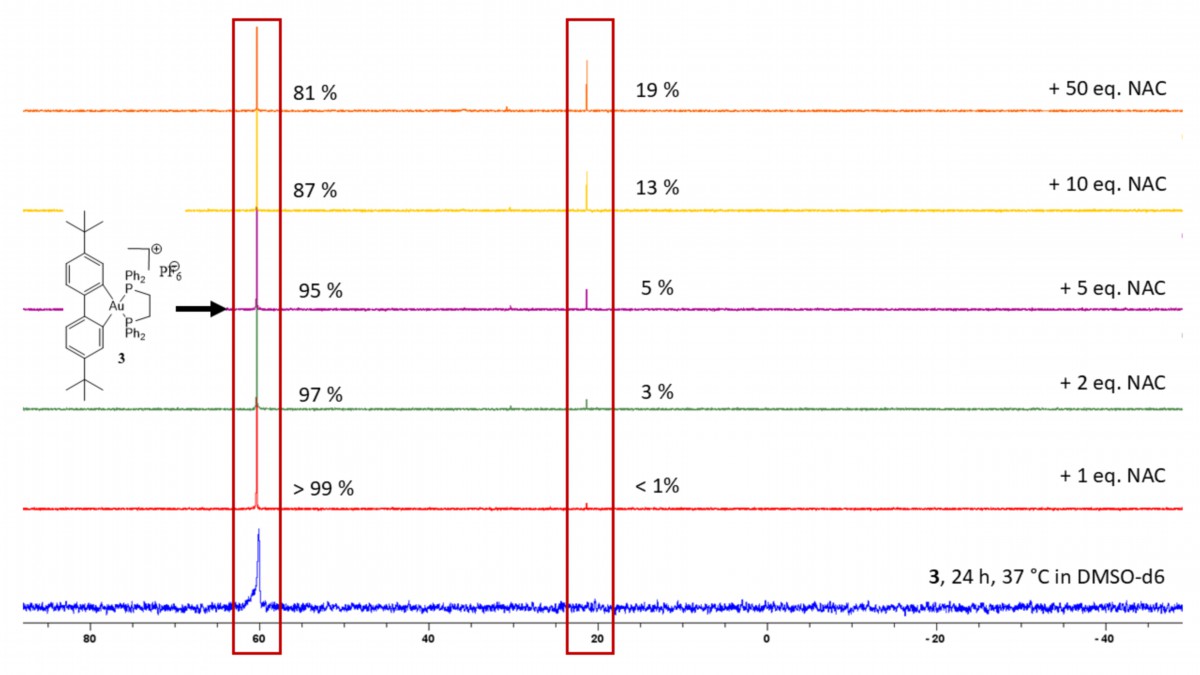


**Figure S24:** ^31^P{^1^H} NMR spectra (400 MHz, 300 K, DMSO-d_6_) of complex **3** alone or in mixture with 1; 2; 5; 10 and 50 equivalents of NAC recorded after 24 h of incubation at 37 °C in DMSO-d_6_.


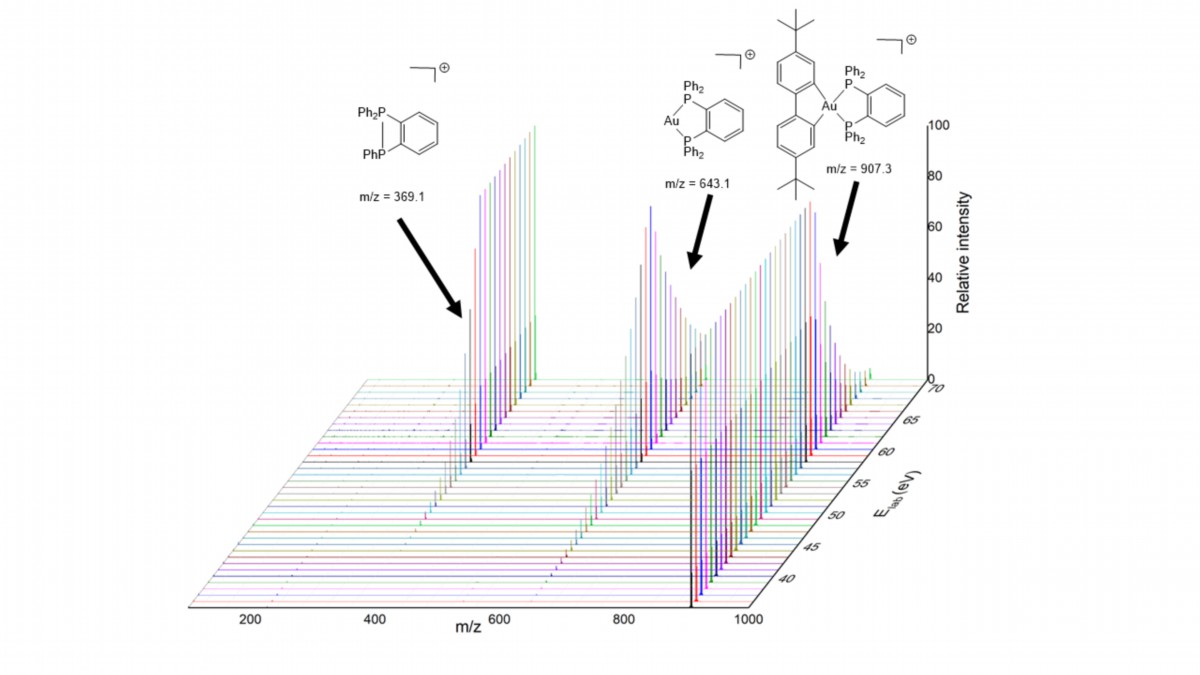


**Figure S25**: MS/MS HCD spectra of the [**1**-PF_6_]^+^ (m/z 907.3) cation for an HCD activation energy E_lab_ comprised between 35 and 71 eV.


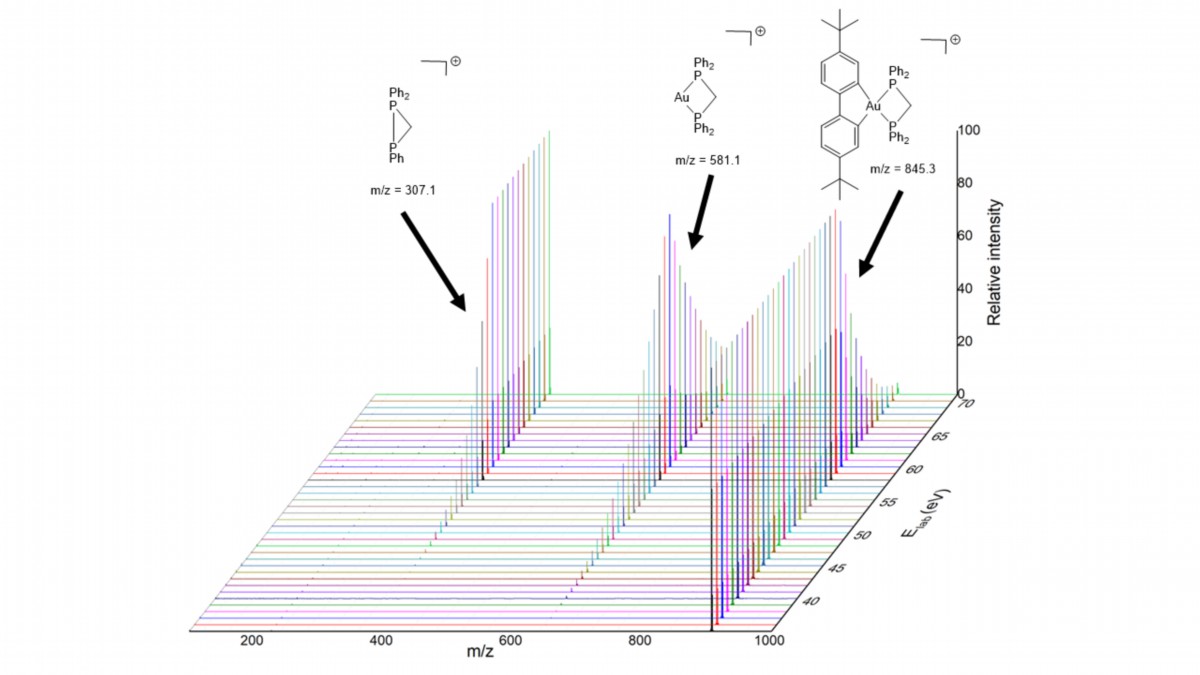


**Figure S26**: MS/MS HCD spectra of the [**2**-PF_6_]^+^ (m/z 845.3) cation for an HCD activation energy E_lab_ comprised between 25 and 60 eV.


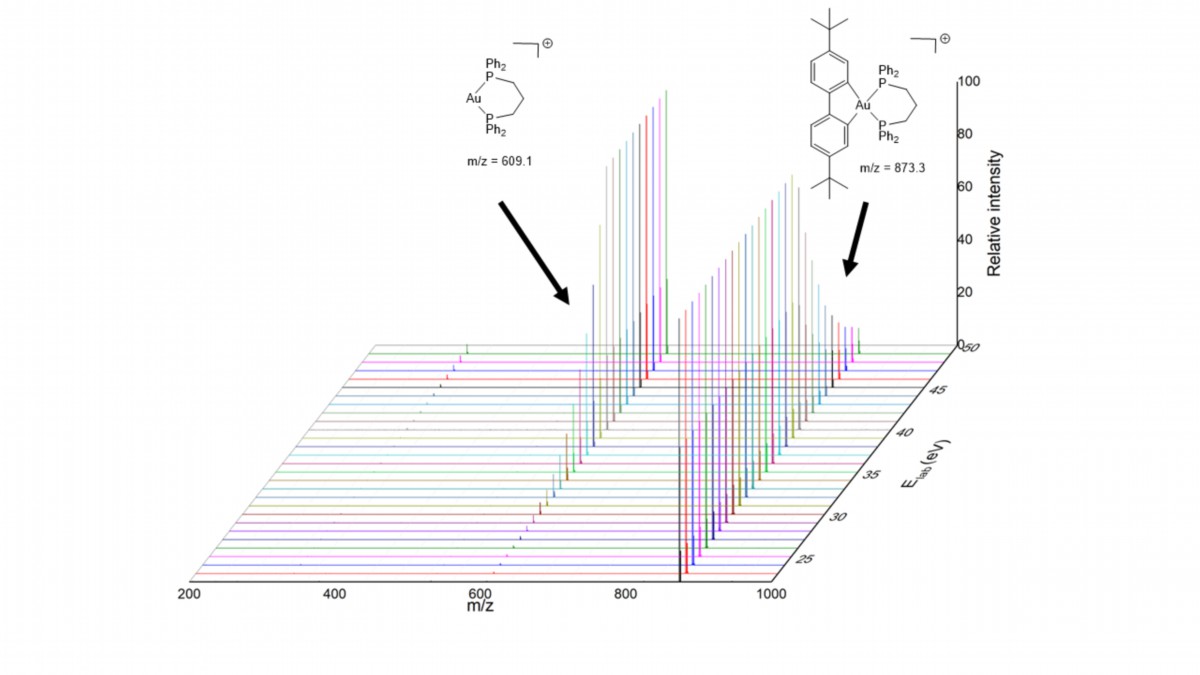


**Figure S27**: MS/MS HCD spectra of the [**4**-PF_6_]^+^ (m/z 873.3) cation for an HCD activation energy E_lab_ comprised between 22 and 50 eV.


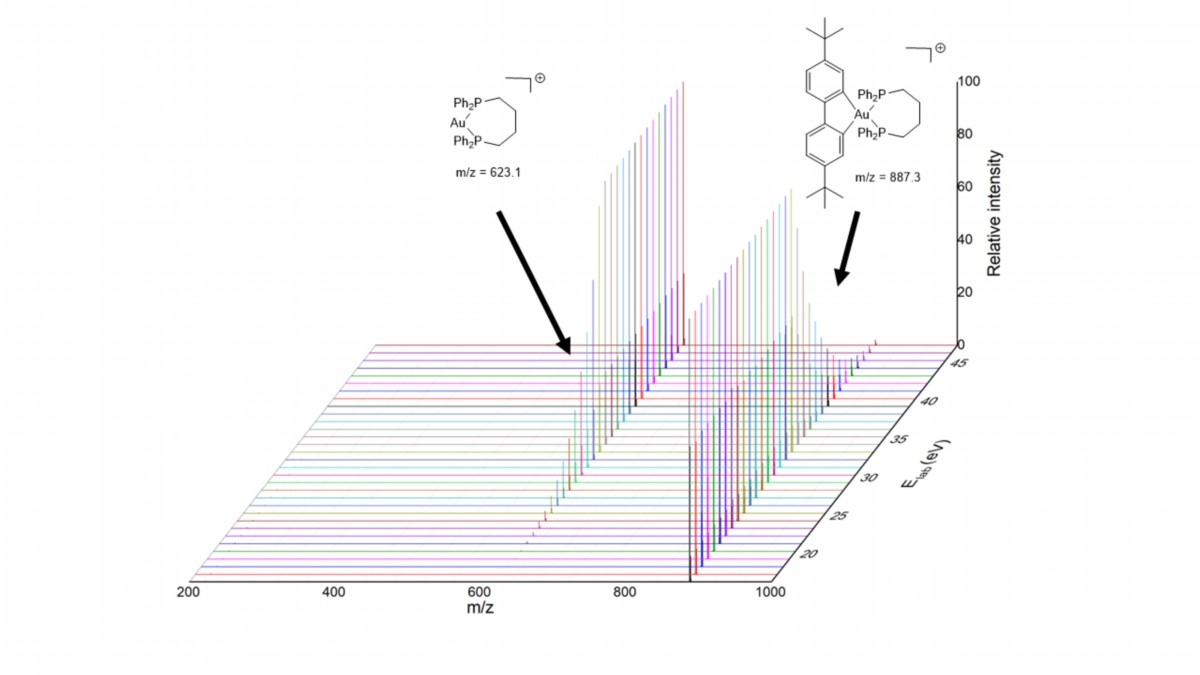


**Figure S28**: MS/MS HCD spectra of the [**5**-PF_6_]^+^ (m/z 887.3) cation for an HCD activation energy E_lab_ comprised between 16 and 47 eV.


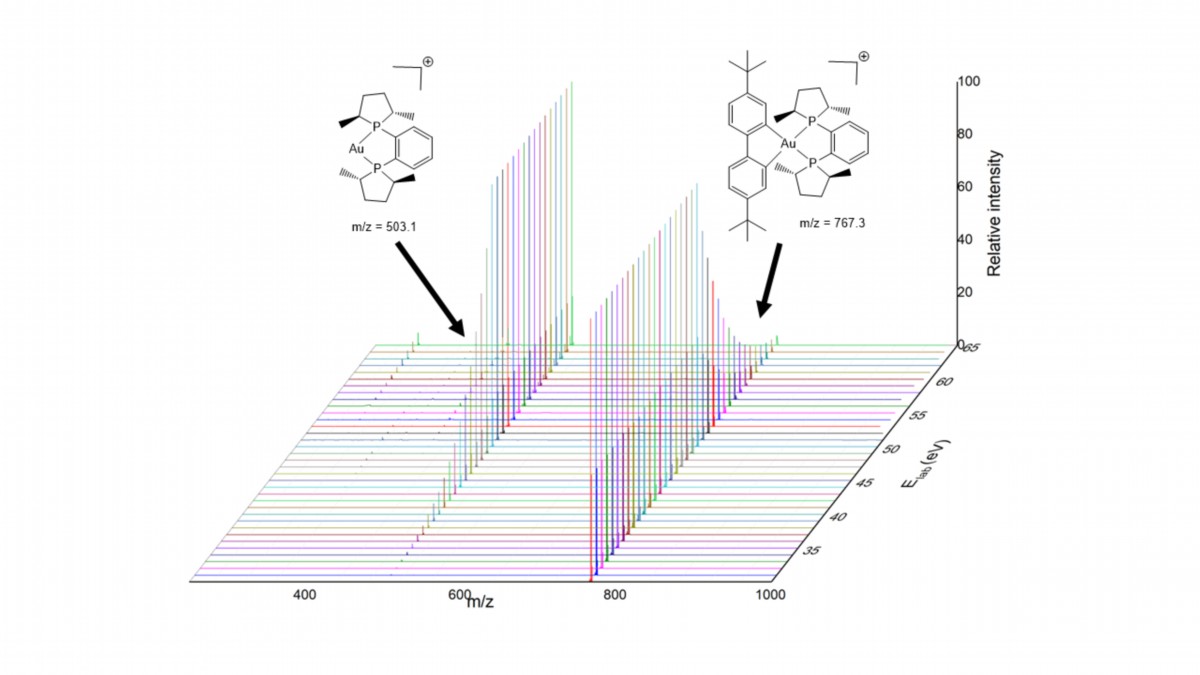


**Figure S29**: MS/MS HCD spectra of the [**6**-PF_6_]^+^ (m/z 767.3) cation for an HCD activation energy E_lab_ comprised between 30 and 65 eV.


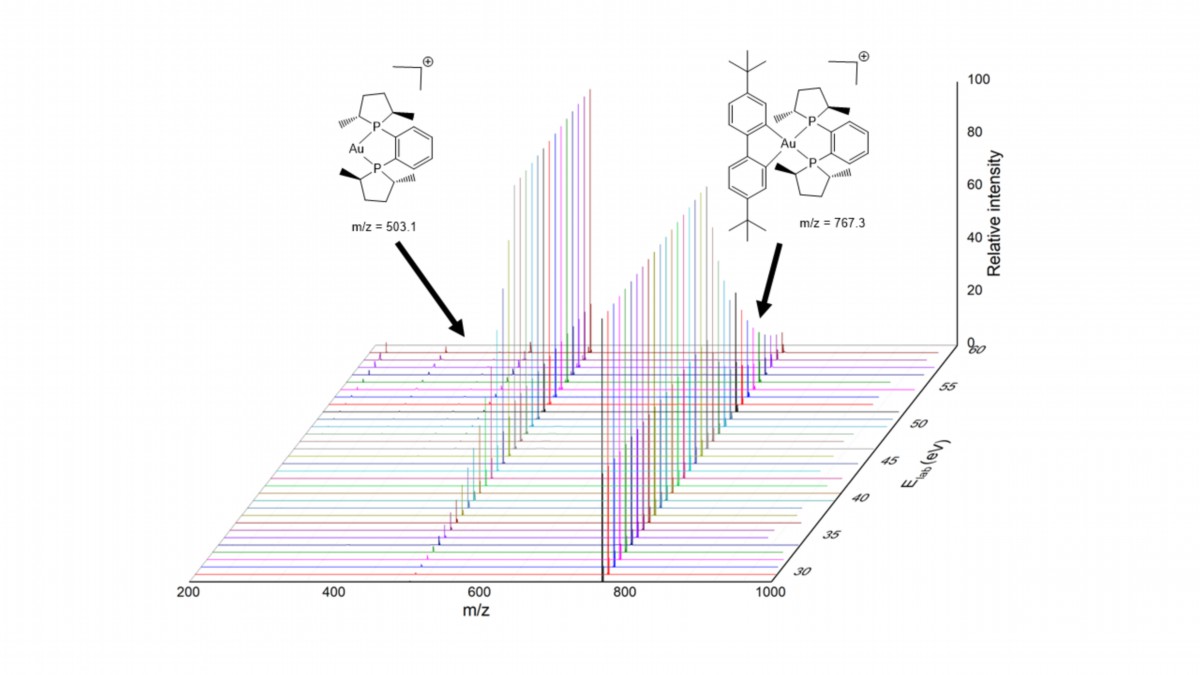


**Figure S30**: MS/MS HCD spectra of the [**7**-PF_6_]^+^ (m/z 767.3) cation for an HCD activation energy E_lab_ comprised between 28 and 60 eV.


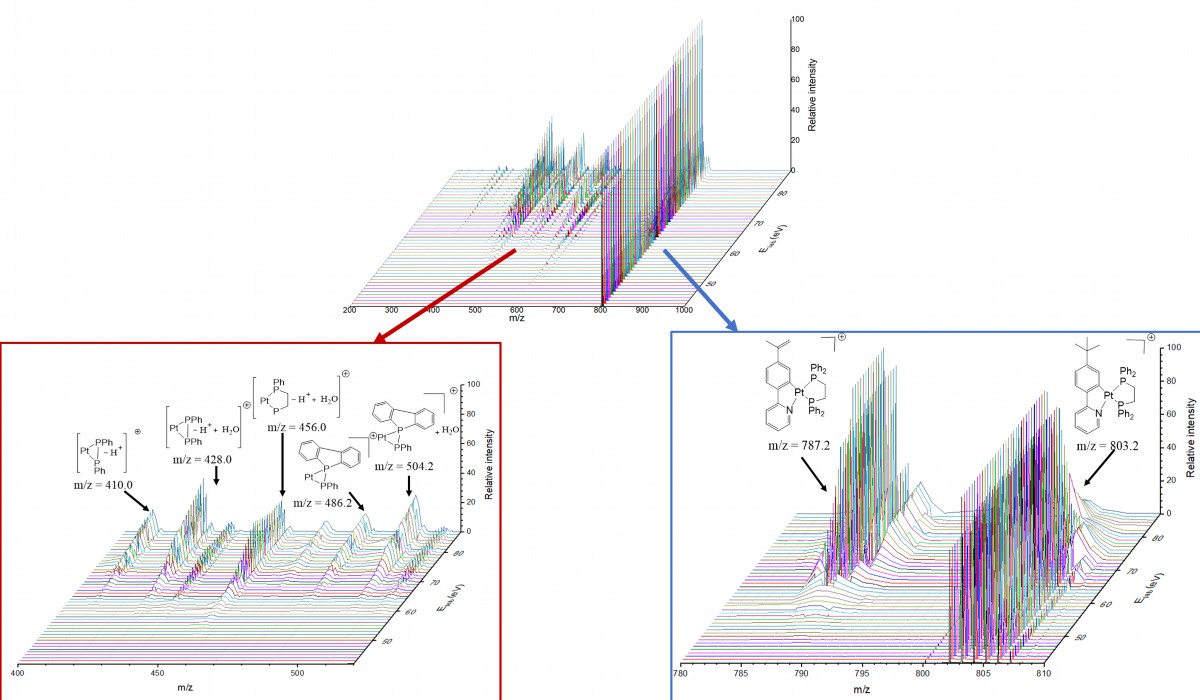


**Figure S31**: MS/MS HCD spectra of the [**8**-PF_6_]^+^ (m/z 803.2) cation for an HCD activation energy E_lab_ comprised between 42 and 87 eV. The red and blue boxes are zooms of the full range MS/MS spectrum shown above.

**Table S2**: Biphenyl reductive elimination energies (E_0_) for Au(III) complexes **1**-**7** ± standard error and formula of the reductive elimination product characterized by in the HCD MS/MS spectra with less than 4 ppm.

| Complexes | E_0_ (eV) | Formula of the detected [Au(P^P)]^+^ cation |
| --- | --- | --- |
| **1** | 2.75 ± 0.2 | C_30_H_24_AuP_2_ |
| **2** | 2.37 ±0.24 | C_25_H_22_AuP_2_ |
| **3** | 2.5 ± 0.25 | C_26_H_24_AuP_2_ |
| **4** | 2.18 ± 0.22 | C_27_H_26_AuP_2_ |
| **5** | 1.83 ± 0.18 | C_28_H_28_AuP_2_ |
| **6** | 2.48 ± 0.25 | C_18_H_28_AuP_2_ |
| **7** | 2.48 ± 0.25 | C_18_H_28_AuP_2_ |


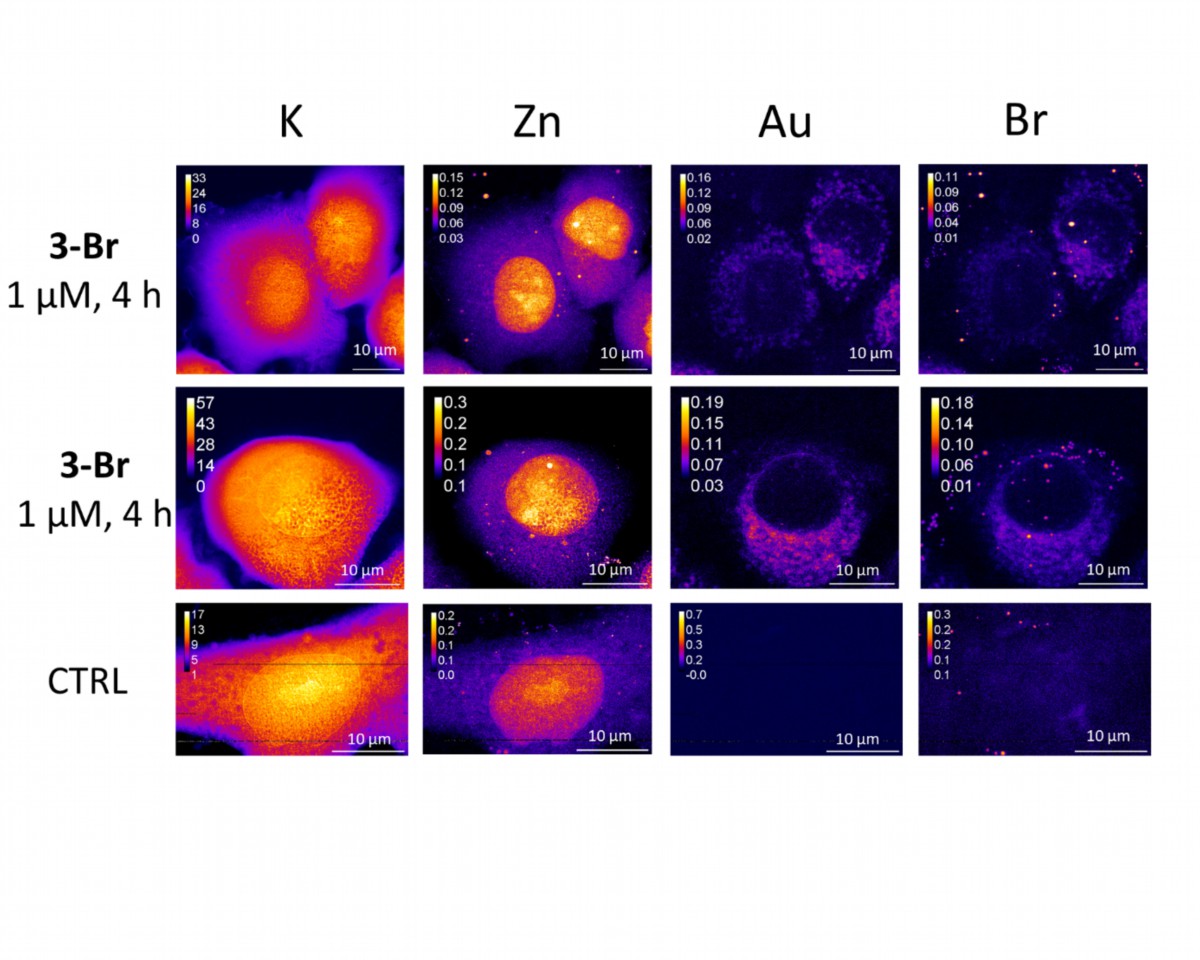


**Figure S32:** Cryo-SR-XRF elemental mapping of K, Zn, Au and Br in A549 cells treated with 1 µM of **3-Br** for 4 h and untreated controls recorded at a resolution of 50 nm/pixel [100 ms]. The elemental concentration in each case is indicated in ng/mm^2^ and is encoded according to the color bar.


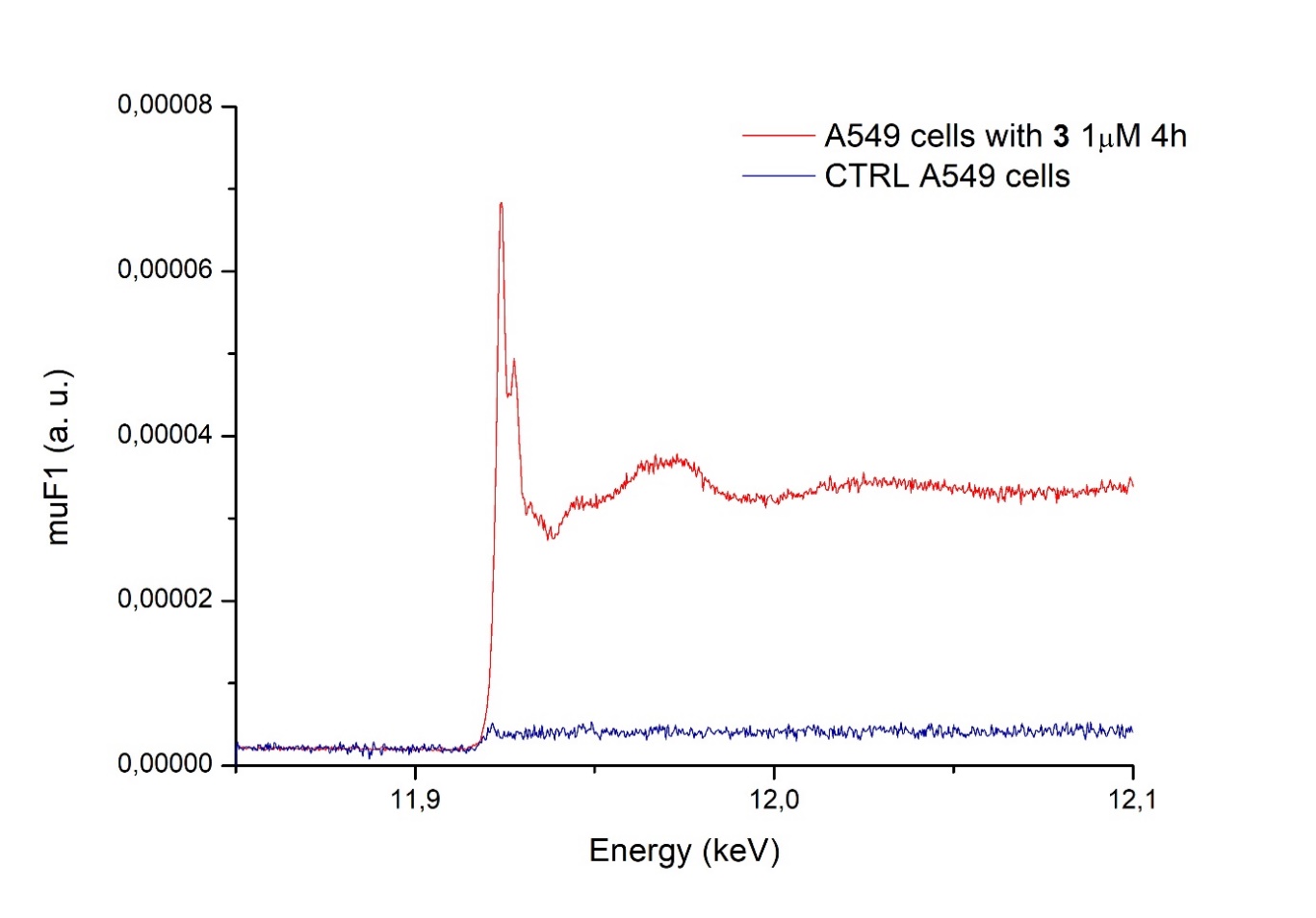


**Figure S33:** XANES spectrum of A549 cells treated with 1 µM of **3** for 4 h compared to spectrum of untreated A549 cells (control) at the L_III_ edge of Au. Both spectra were recorded at 4 K in fluorescence mode.


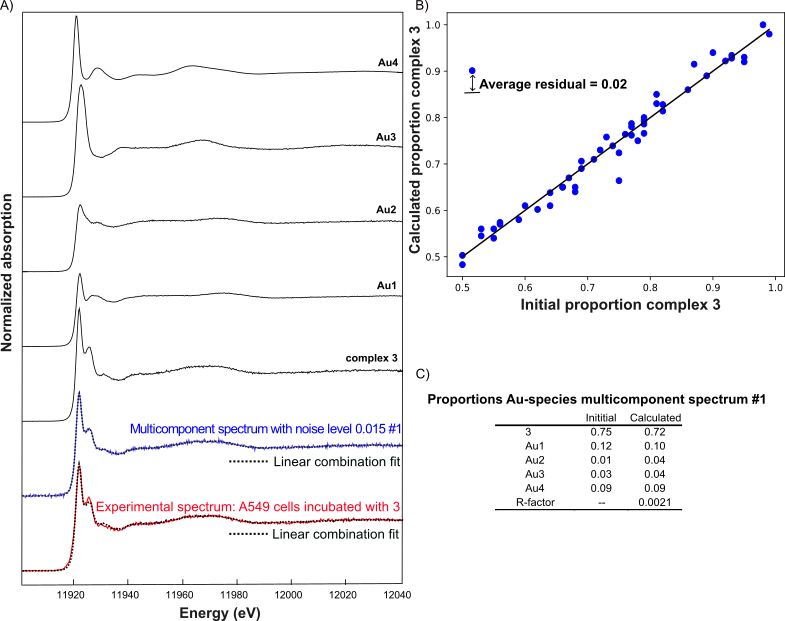


**Figure S34:** Estimation of the standard deviation of Linear Combination Fitting (LCF) results. A) Example of one of the 50 multicomponent spectra created with the references of complexes **3**, and **Au1-4**. A similar noise was added (0.015) as the one observed in experimental spectrum of A549 cells incubated with **3**. B) The proportion calculated by LCFs versus the initial ratio was compared by linear regression. The average residual value (calculated by the average absolute value of the distance of the datapoints to the linear that goes through the points (0, 0) and (1, 1) is the standard deviation of the linear combination fitting procedure (2 %). C) Example of the initial and calculated proportion (calculated by LCF) of all species for multicomponent spectrum #1 together with the R-factor of the fit ((=∑[µ_exp_-µ_fit_]^2^/∑[µ_exp_]^2^).

**Table S3:** Linear combination fitting (LCF) results of **3** in A549 cells and **3** in DMEM culture medium.

|  | **Proportion of species determined by LCF** | | | | | |
| --- | --- | --- | --- | --- | --- | --- |
|  | **3** | Au1 | Au2 | Au3 | Au4 | R-factor |
| A549 cells | 0.84 |  |  | 0.16 |  | 0.00418 |
|  | 0.80 |  |  | 0.15 | 0.05 | 0.00423 |
|  | 0.96 | 0.00 | 0.00 | 0.04 | 0.00 | 0.00594 |
|  | 1.00 | 0.00 | 0.00 | 0.00 | 0.00 | 0.00602 |
| DMEM | 0.91 | 0.02 | 0.00 | 0.07 | 0.00 | 0.00603 |


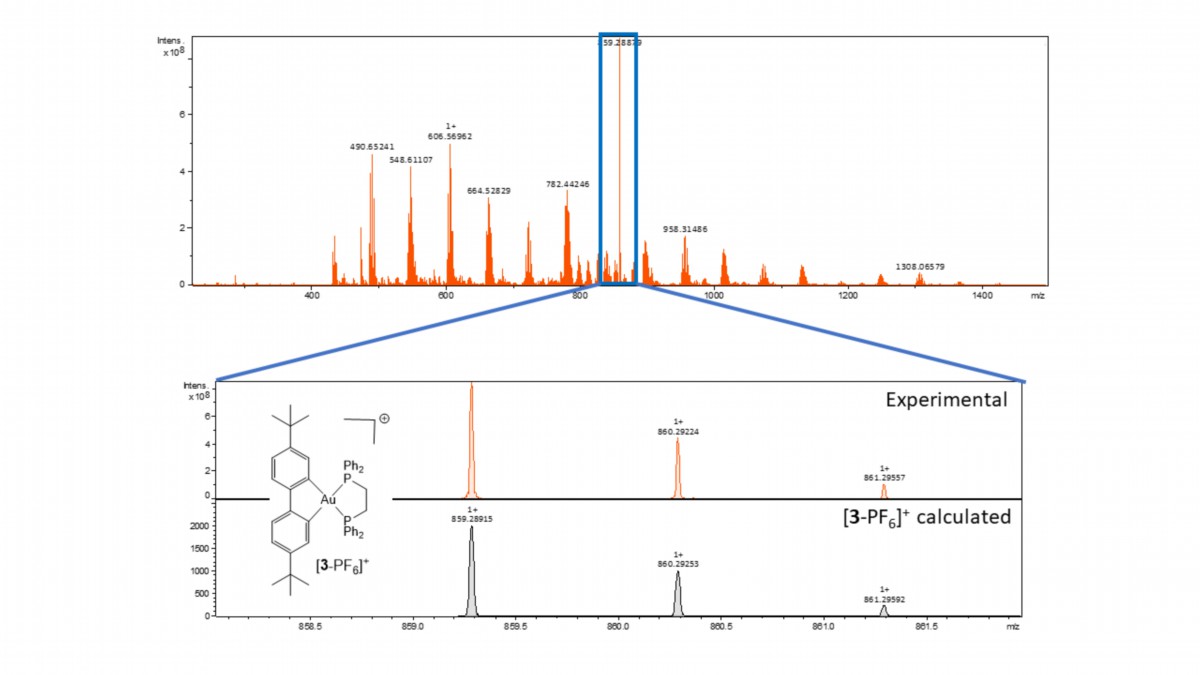


**Figure S35**: *Top:* HR-ESI-MS spectrum of cytosolic fraction of A549 cells lysate treated with 1 µM of complex **3** for 4 h. *Bottom*: Comparison between experimental isotopic pattern of signal at m/z = 859.28876 and calculated isotopic pattern of cation [**3**-PF_6_]^+^.


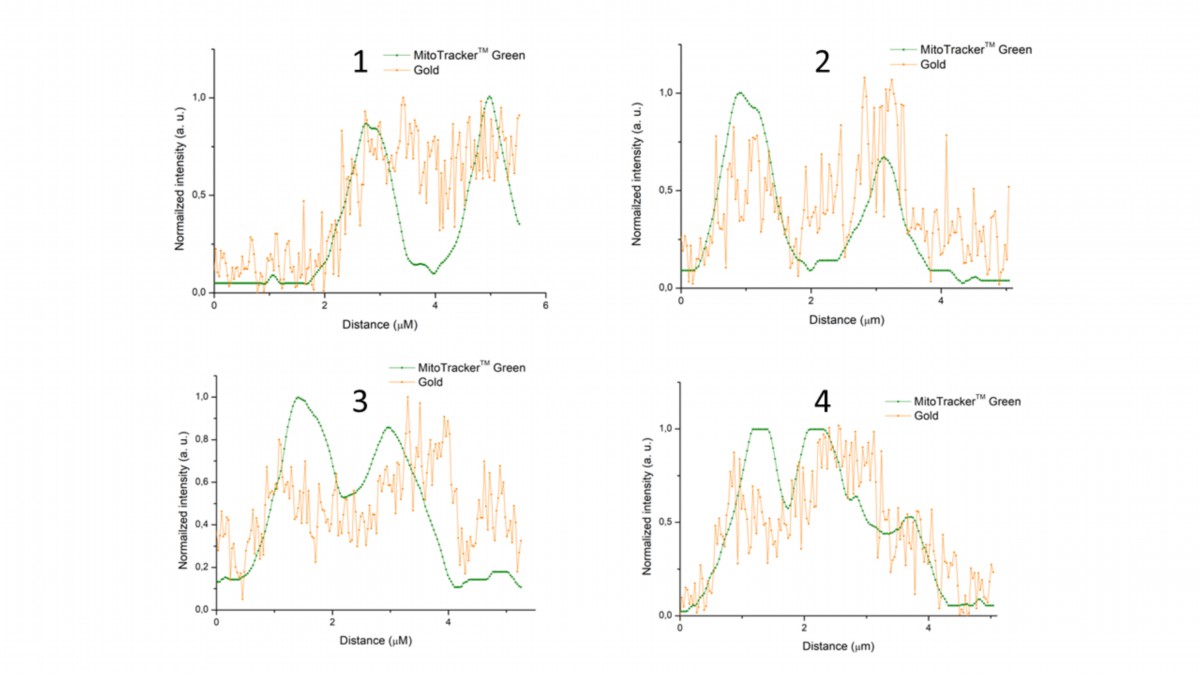


**Figure S36**: Comparison of the normalized intensities of Au XRF and MitoTracker^TM^ Green signals along lines across images of regions 1-4 defined in figure 5A. Image analysis has been carried out using Fiji software.


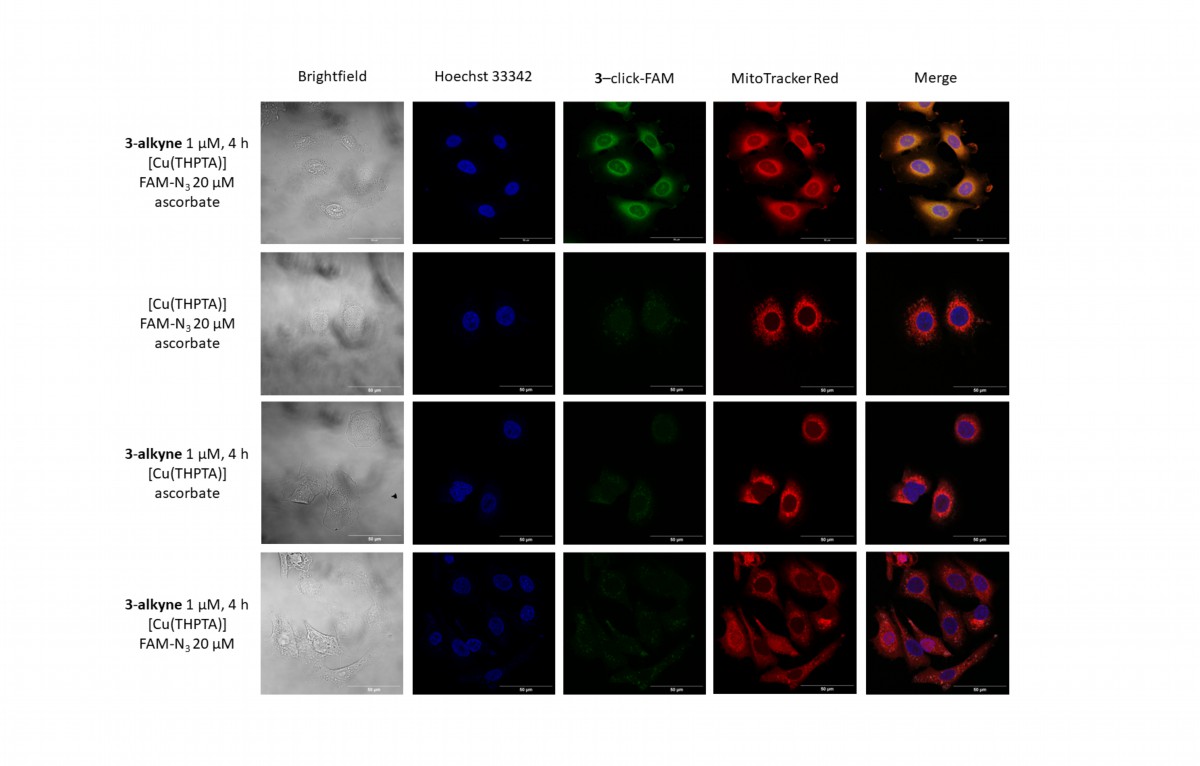


**Figure S37:** Confocal fluorescence microscopy images of A549 cells exposed to 1 µM of **3**-**alkyne** for 4 h, fixed and “clicked” *in cellulo* with FAM-N_3_ (green) and control experiments without **3**-**alkyne**, FAM-N_3_ or ascorbate. Staining of DNA (Hoechst 33342, blue) and mitochondria (MitoTracker^TM^ Red, red).

*

*

*


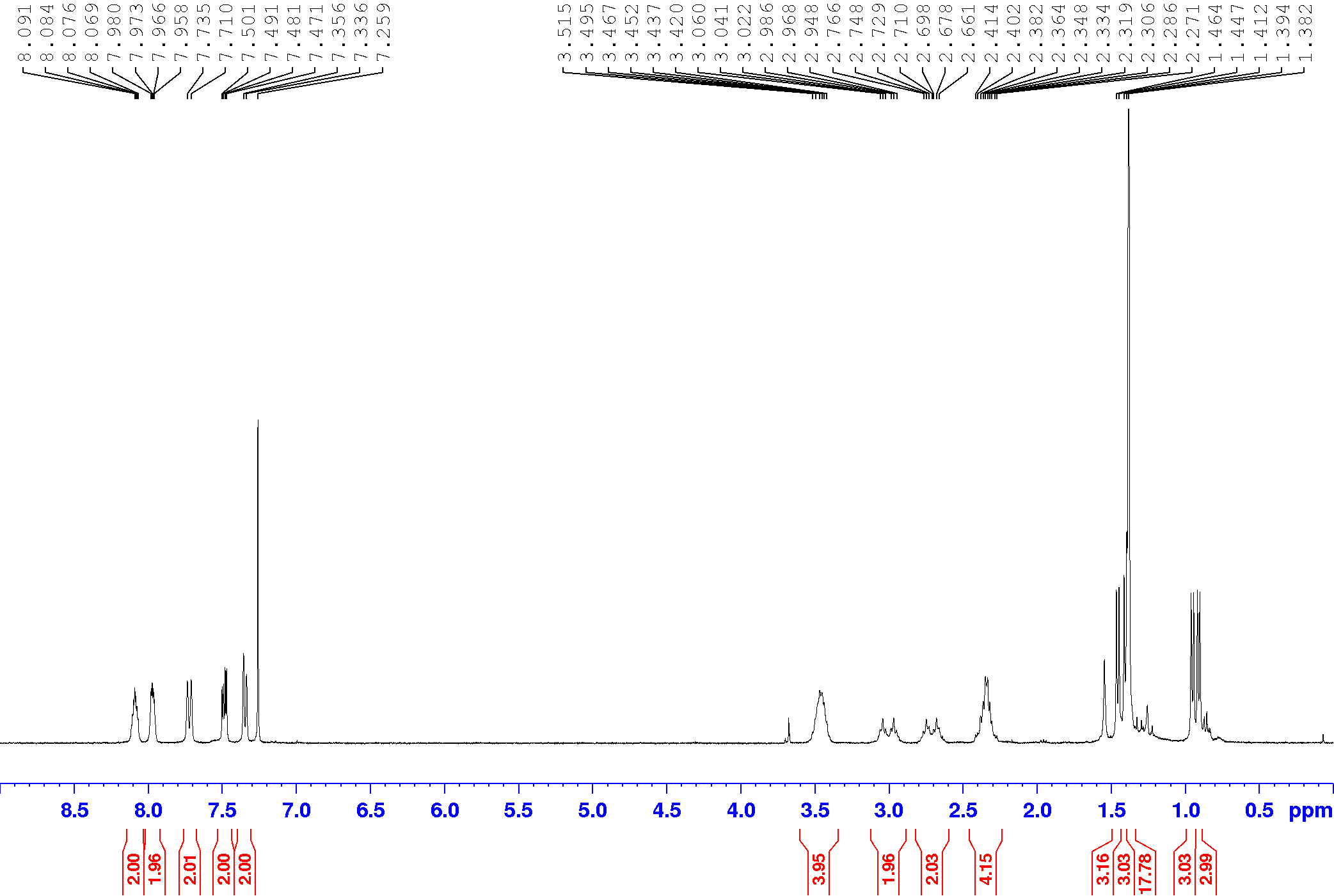


**Figure S38:** ^1^H NMR spectra (400 MHz, 300 K, CDCl_3_) of complex **6**. Orange stars show signals of solvent impurities.


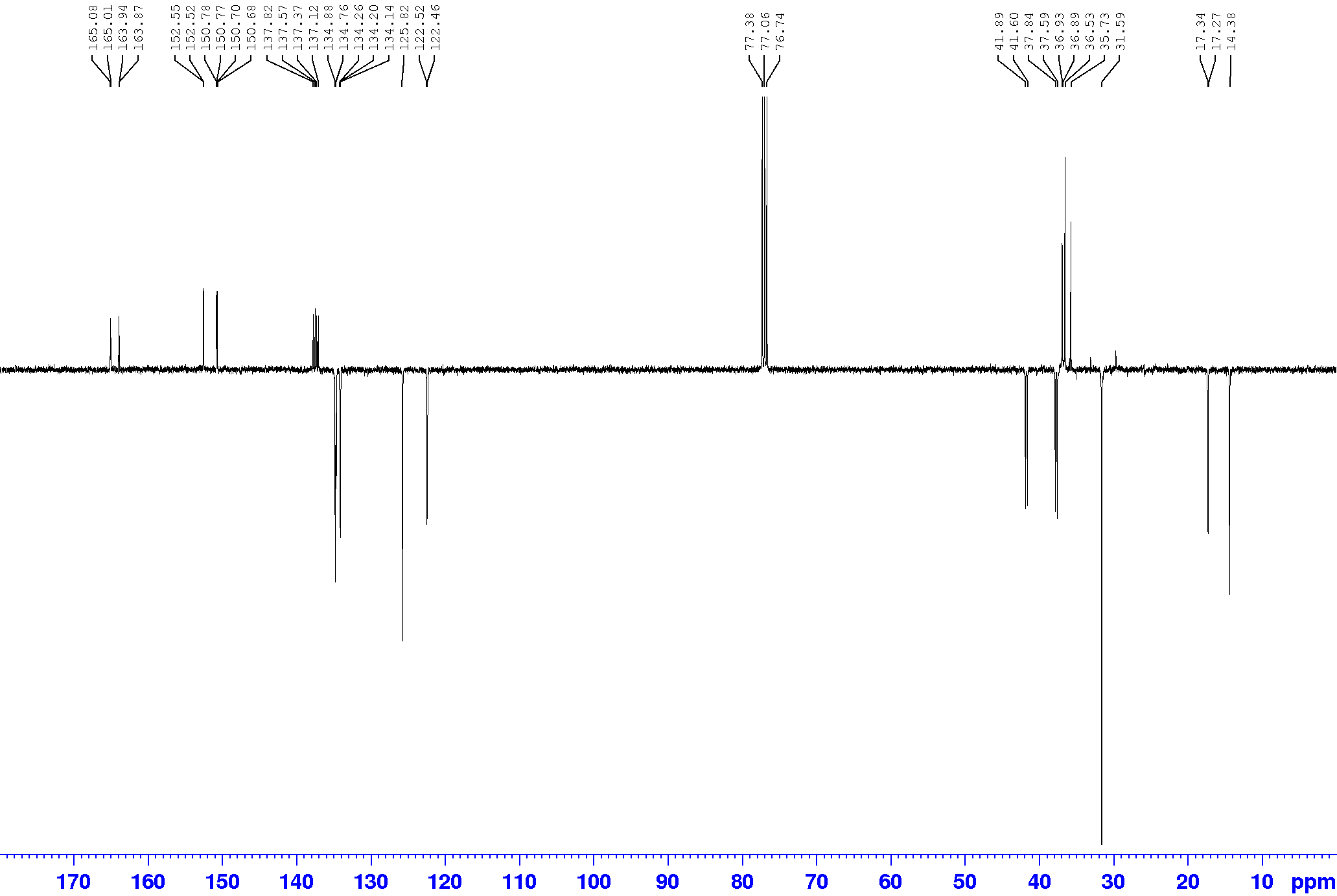


**Figure S39:** Jmod ^13^C{^1^H} NMR spectra (100 MHz, 300 K, CDCl_3_) of complex **6**.


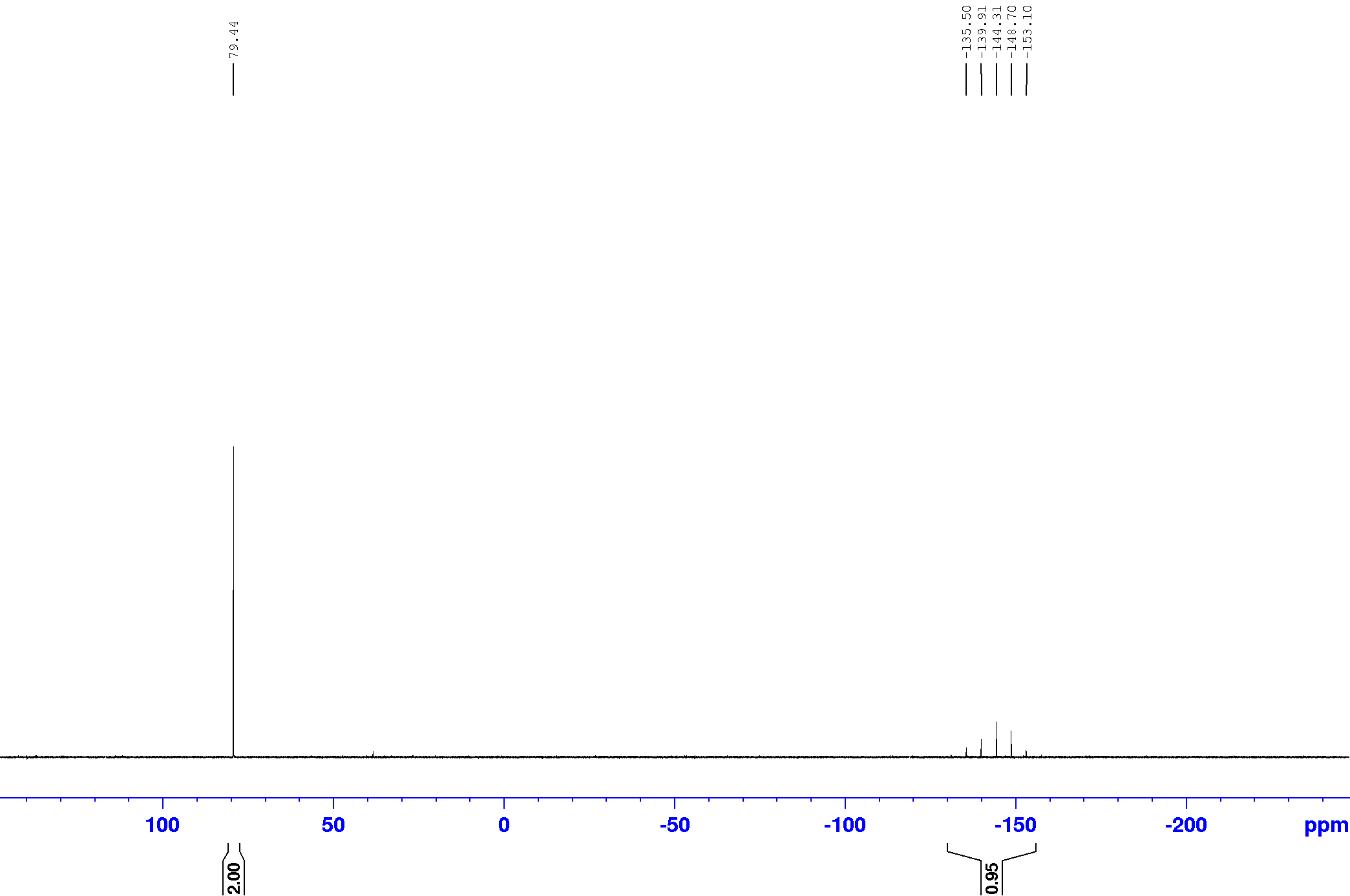


**Figure S40:** ^31^P{^1^H} NMR spectra (162 MHz, 300 K, CDCl_3_) of complex **6**.

**Figure S41**: HPLC chromatogram of complex **6** in acetonitrile eluted with H_2_O-0.1% TFA/acetonitrile-0.08% TFA 20/80 (peak labels: retention time, % area).

*


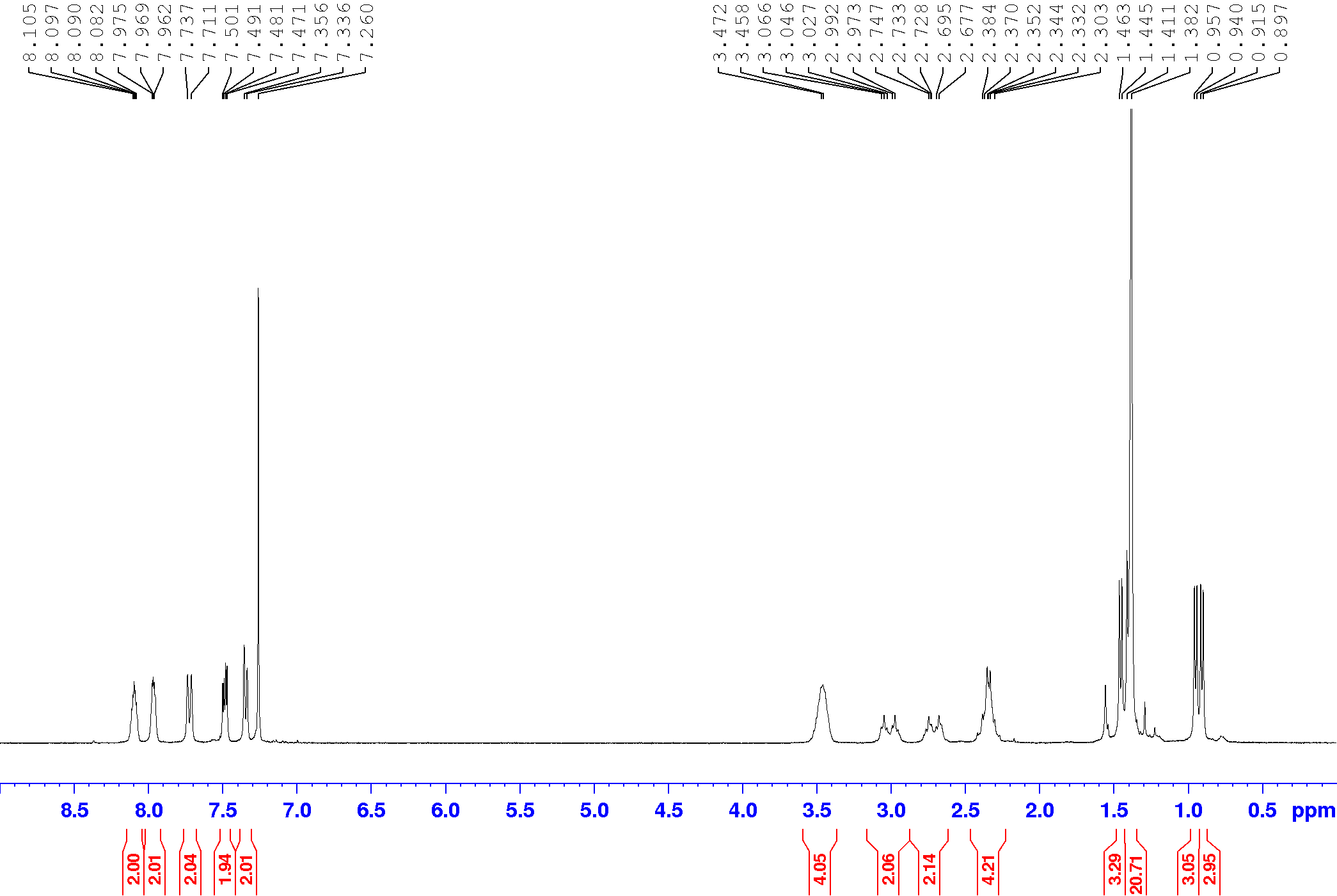


**Figure S42:** ^1^H NMR spectra (400 MHz, 300 K, CDCl_3_) of complex **7**. Orange stars show signals of solvent impurities.


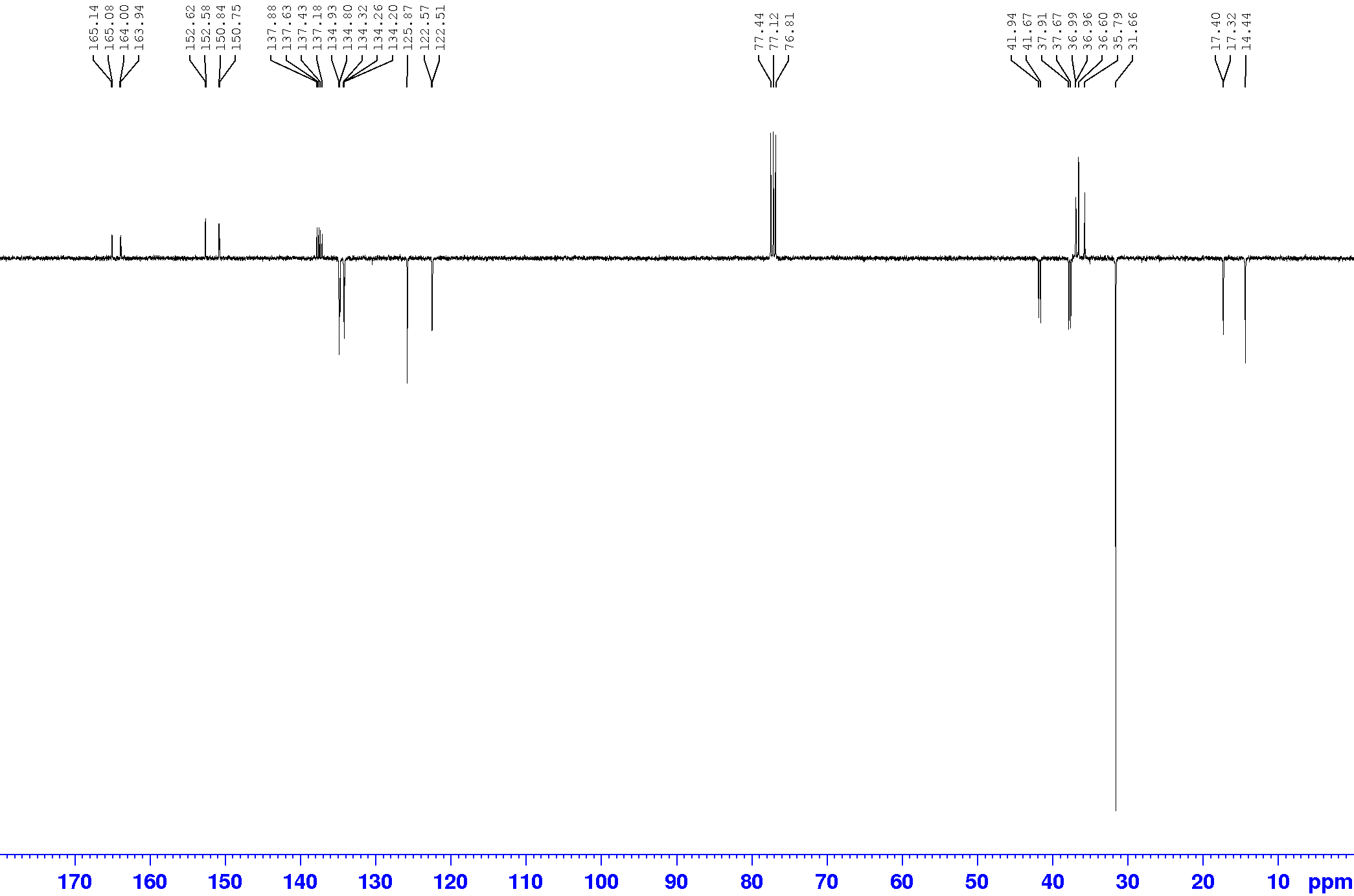


**Figure S43:** Jmod ^13^C{^1^H} NMR spectra (100 MHz, 300 K, CDCl_3_) of complex **7**.


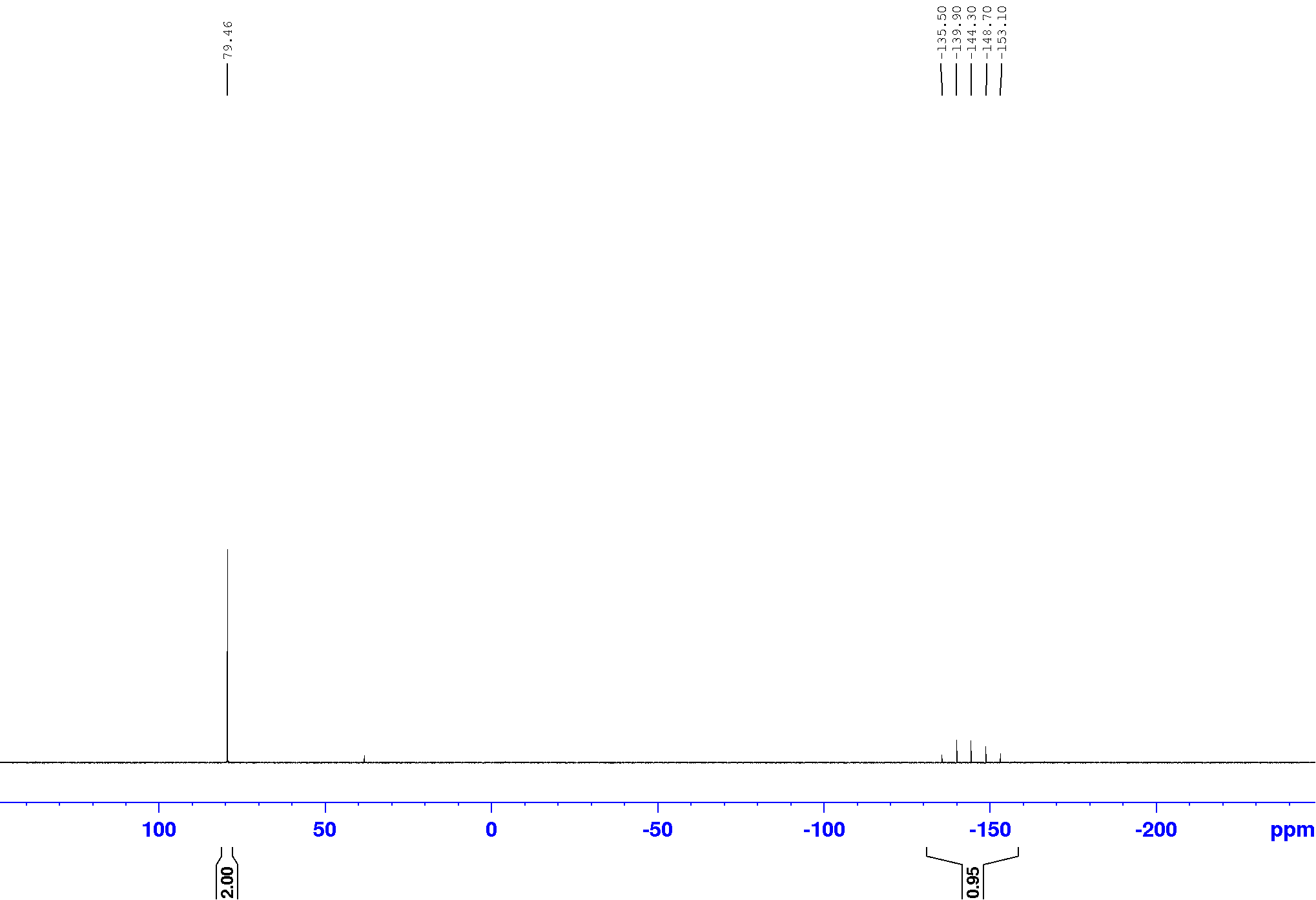


**Figure S44:** ^31^P{^1^H} NMR spectra (162 MHz, 300 K, CDCl_3_) of complex **7**.

**Figure S45**: HPLC chromatogram of complex **7** in acetonitrile eluted with H_2_O-0.1% TFA/acetonitrile-0.08% TFA 20/80 (peak labels: retention time, % area).

**Optimized geometries for complexes 1-7 and their fragment**

Coordinates of **1.**

Au 0.01863200 0.54518700 -0.26321300

C -1.29615800 2.14711200 -0.05657800

C -0.66159600 3.39125400 0.13169200

C -2.67694800 2.04280400 -0.03869800

C -1.46526400 4.51115300 0.32855900

C 0.79837600 3.36724900 0.11811100

C -3.49323500 3.17258500 0.17077100

H -3.16443400 1.07539100 -0.19049400

C -2.85607200 4.40150400 0.34923400

H -1.00465700 5.49324100 0.47718900

C 1.38846400 2.10369800 -0.08527700

C 1.64163700 4.45997400 0.30260000

H -3.44430500 5.30708800 0.51355900

C 2.76577500 1.95717800 -0.10048200

C 3.02845600 4.30617800 0.29750100

H 1.21575300 5.45578000 0.46292900

C 3.62240100 3.05865500 0.10013900

H 3.21896800 0.97718000 -0.27474100

H 3.64768200 5.19201500 0.45547400

C 5.13338100 2.83905800 0.08224600

C 5.50965100 1.76239100 1.10601100

C 5.55407800 2.38261300 -1.31801400

C 5.90042400 4.11197800 0.42577900

H 5.18996900 2.05160700 2.12193700

H 5.05789000 0.78318900 0.87167400

H 6.60354000 1.61905900 1.12434500

H 5.32184100 3.15485100 -2.07039700

H 6.64100300 2.19175900 -1.35042700

H 5.04204000 1.45404500 -1.62097500

H 6.98379800 3.90969300 0.41207500

H 5.71098300 4.91781000 -0.30225400

H 5.64673000 4.48762400 1.43119600

C -5.01072100 3.00391000 0.17607600

C -5.46553100 2.57356800 -1.22201000

C -5.40891700 1.93410900 1.19830500

C -5.72936800 4.29943300 0.53963900

H -5.22125600 3.34632200 -1.97009200

H -4.98470400 1.63429000 -1.54240200

H -6.55805600 2.41532700 -1.24038800

H -5.07213200 2.20911200 2.21226100

H -6.50629300 1.82230900 1.22511800

H -4.98649900 0.94378500 0.95744200

H -6.81882900 4.13237600 0.54472300

H -5.44525200 4.66097500 1.54209600

H -5.52772500 5.10289300 -0.18774100

P -1.60033100 -1.35503500 -0.32372200

P 1.55956500 -1.41337400 -0.27009800

C 0.67039300 -2.78467400 -1.09645700

C -0.73521000 -2.74598600 -1.14842100

C 1.76629800 -1.90284400 1.47072700

C 1.44868600 -3.18527900 1.92992500

C 2.20187600 -0.92331100 2.37609900

C 1.56660700 -3.48428300 3.28577500

H 1.09986600 -3.95429600 1.23227000

C 2.32809400 -1.23410000 3.72593100

H 2.43984400 0.08914700 2.02554800

C 2.00569600 -2.51238800 4.18160700

H 1.31312700 -4.48622600 3.64311400

H 2.67252400 -0.47018000 4.42789900

H 2.09771200 -2.75172100 5.24453100

C 3.20346200 -1.39477600 -1.04323500

C 4.34512000 -1.83929900 -0.36949300

C 3.29974400 -0.93799600 -2.36522800

C 5.57725400 -1.83464400 -1.02330600

H 4.27556400 -2.19177300 0.66518900

C 4.52959200 -0.94635500 -3.01279300

H 2.40721600 -0.57089900 -2.88679100

C 5.66841000 -1.39545100 -2.34146800

H 6.47038100 -2.18226000 -0.49731400

H 4.60394200 -0.59329900 -4.04467000

H 6.63580600 -1.39594800 -2.85087900

C -1.91111700 -1.92405100 1.37883200

C -2.14892700 -3.27413900 1.66752500

C -1.89119000 -0.98326000 2.41620200

C -2.36381900 -3.67621400 2.98203700

H -2.16006700 -4.01854100 0.86361900

C -2.10444900 -1.39336400 3.73035100

H -1.70498400 0.07561800 2.19914400

C -2.33858400 -2.73716000 4.01295500

H -2.55112900 -4.73035400 3.20407500

H -2.08529000 -0.65641300 4.53741000

H -2.50382800 -3.05677100 5.04552200

C -3.20211200 -1.23553900 -1.17287700

C -4.40204200 -1.60302500 -0.55609000

C -3.20719300 -0.75522100 -2.48987900

C -5.59992600 -1.50592000 -1.26441000

H -4.40616600 -1.96480900 0.47770000

C -4.40425800 -0.66830100 -3.19103900

H -2.26917600 -0.44523700 -2.96671500

C -5.60049100 -1.04662200 -2.57864400

H -6.53817100 -1.79406300 -0.78287000

H -4.40717100 -0.29705300 -4.21915400

H -6.54142200 -0.97355300 -3.13060600

C 1.35641700 -3.85399300 -1.67890400

C -1.43184300 -3.77061600 -1.79514400

C -0.73853400 -4.83059100 -2.37620600

H 2.45092200 -3.88386000 -1.64647100

C 0.65216700 -4.87445000 -2.31449500

H -2.52532900 -3.73539500 -1.85414700

H -1.29006000 -5.62464900 -2.88642400

H 1.19570000 -5.70417700 -2.77386500

Coordinates of **1** fragment loss of (C^C).

Au 0.00910500 -0.61733400 -1.78720800

P -1.72312800 -0.22696500 -0.12198700

P 1.70824100 -0.27367500 -0.08659100

C 0.68129900 -0.85128600 1.33375400

C -0.74114000 -0.85332100 1.31427800

C 1.88342400 1.52545100 0.11908700

C 1.54419000 2.17885900 1.30855500

C 2.37431600 2.26307200 -0.96709000

C 1.69840700 3.56065000 1.40672800

H 1.15743500 1.61239600 2.16255700

C 2.53590300 3.63991700 -0.85828700

H 2.63527600 1.75380300 -1.90279900

C 2.19439200 4.28926800 0.32829100

H 1.43053300 4.07000200 2.33666100

H 2.92599500 4.21079200 -1.70505600

H 2.31646000 5.37263600 0.41138000

C 3.34865100 -1.01350000 0.13513600

C 4.42973800 -0.27849500 0.63439000

C 3.51275000 -2.36214400 -0.20610900

C 5.66641700 -0.89972600 0.79813300

H 4.30851200 0.77778300 0.89702500

C 4.74750800 -2.97645600 -0.03102900

H 2.66861500 -2.93287300 -0.61135400

C 5.82446800 -2.24410600 0.46941200

H 6.51263000 -0.32701800 1.18677100

H 4.87477400 -4.02955200 -0.29496100

H 6.79740700 -2.72571200 0.59853400

C -1.86694000 1.56615000 0.16773100

C -2.07235200 2.06959200 1.45956500

C -1.76901300 2.44864000 -0.91312100

C -2.17930800 3.44183300 1.66119100

H -2.14767400 1.38706300 2.31351300

C -1.87405300 3.82212700 -0.70592200

H -1.60651000 2.06026000 -1.92551100

C -2.07653500 4.31741800 0.58007400

H -2.34337600 3.83163800 2.66952500

H -1.79626700 4.50752300 -1.55387300

H -2.15783600 5.39569500 0.74241500

C -3.37216300 -0.96471000 0.03900100

C -4.50856500 -0.19469300 0.30620500

C -3.49350400 -2.34665600 -0.16418200

C -5.75617100 -0.81183000 0.38608100

H -4.42532700 0.88717100 0.45082500

C -4.73928300 -2.95517100 -0.07315800

H -2.60599000 -2.94959100 -0.39192200

C -5.87172500 -2.18683800 0.20133500

H -6.64444200 -0.20973400 0.59461700

H -4.83100900 -4.03368800 -0.22587300

H -6.85258200 -2.66539000 0.26489900

C 1.34372400 -1.33289700 2.46990400

C -1.43091100 -1.34498300 2.42963300

C -0.75263200 -1.81176400 3.55193400

H 2.43848800 -1.34216300 2.49257800

C 0.63771200 -1.80352000 3.57311700

H -2.52598600 -1.36411500 2.42073200

H -1.31696300 -2.18465000 4.41062500

H 1.18020600 -2.16854600 4.44899700

Coordinates of **2.**

Au 0.01233700 0.45097800 0.05174000

C 1.35534300 1.96069500 -0.34491100

C 0.77144700 3.19065100 -0.70753100

C 2.72560000 1.79584700 -0.26661200

C 1.63192500 4.24609000 -0.99728400

C -0.69213800 3.20026300 -0.72521700

C 3.59757700 2.86334000 -0.55810700

H 3.15018400 0.83012200 0.03125800

C 3.01828400 4.08074100 -0.92398200

H 1.22353400 5.22066500 -1.28497500

C -1.30113300 1.97920700 -0.37369000

C -1.53051700 4.26592700 -1.04030300

H 3.65201400 4.93850900 -1.16053300

C -2.67489800 1.83142800 -0.33071100

C -2.92002200 4.11803400 -1.00305200

H -1.10228800 5.23401400 -1.32098400

C -3.52478600 2.90944900 -0.64875200

H -3.11733500 0.87051100 -0.04134900

H -3.53628300 4.98268400 -1.25957500

C -5.03648200 2.70817600 -0.57572000

C -5.44412400 1.53922400 -1.47879100

C -5.42979700 2.39777100 0.87278200

C -5.79998800 3.94931900 -1.02669700

H -5.15917600 1.73019000 -2.52712300

H -4.98026600 0.58648800 -1.17050600

H -6.53745400 1.39571700 -1.44620400

H -5.14976800 3.22724000 1.54364200

H -6.52095900 2.25203600 0.95015800

H -4.94398700 1.48173000 1.25038500

H -6.88451300 3.76103400 -0.97146500

H -5.58752900 4.82081500 -0.38563700

H -5.56502900 4.22122100 -2.06930800

C 5.10348600 2.63506400 -0.45334300

C 5.45589000 2.24863600 0.98736900

C 5.51445200 1.50149000 -1.39935700

C 5.89670200 3.88326000 -0.82604800

H 5.17106100 3.04845600 1.69152000

H 4.95030900 1.32114300 1.30823300

H 6.54274900 2.08339600 1.08330500

H 5.25304000 1.74164000 -2.44372800

H 6.60491000 1.34084800 -1.35171500

H 5.03159200 0.54346300 -1.13967300

H 6.97643400 3.67927200 -0.74105400

H 5.70265200 4.20030900 -1.86427400

H 5.67015400 4.73047600 -0.15770400

P 1.40196600 -1.54744500 0.59934100

P -1.42661400 -1.50667500 0.64931700

C -0.00520200 -2.32291800 1.53548000

C -1.87227300 -2.61364300 -0.71317600

C -2.10480200 -3.97846500 -0.49226300

C -1.96352900 -2.08933000 -2.00735800

C -2.41680800 -4.81001300 -1.56207200

H -2.04893400 -4.39376700 0.52099200

C -2.27968800 -2.92765900 -3.07490500

H -1.78539800 -1.02112600 -2.18135500

C -2.50155200 -4.28428200 -2.85230800

H -2.59931600 -5.87419400 -1.39027600

H -2.35106100 -2.51721800 -4.08537000

H -2.74701700 -4.94092900 -3.69140700

C -2.84594700 -1.34568100 1.76598900

C -4.09112300 -1.90996200 1.46764500

C -2.70328600 -0.51978400 2.89052300

C -5.17671000 -1.67025100 2.30826800

H -4.21839400 -2.53547800 0.57837100

C -3.78855500 -0.29350700 3.72882900

H -1.74285500 -0.03381200 3.10413700

C -5.02554800 -0.87044800 3.43809300

H -6.14841000 -2.11387900 2.07600600

H -3.67362900 0.34661500 4.60726200

H -5.88015200 -0.68591200 4.09437400

C 1.70598800 -2.58135600 -0.85669900

C 1.61235000 -3.97768400 -0.81409600

C 2.03982500 -1.93953600 -2.05645000

C 1.83837200 -4.72311600 -1.96709800

H 1.36448100 -4.49184300 0.12113500

C 2.27465200 -2.69203300 -3.20450300

H 2.10993400 -0.84546100 -2.09404800

C 2.16765900 -4.08038200 -3.16043000

H 1.76083200 -5.81314600 -1.93419300

H 2.53565300 -2.18971300 -4.13951600

H 2.34435600 -4.66945000 -4.06445800

C 2.89761000 -1.51664600 1.62207700

C 4.05483400 -2.21547000 1.26172100

C 2.90260600 -0.68244000 2.74940800

C 5.20217300 -2.09680600 2.04406200

H 4.06308900 -2.85298500 0.37157400

C 4.04792400 -0.57862800 3.53010000

H 2.01165300 -0.09610300 3.00828400

C 5.19736200 -1.28719100 3.17731600

H 6.10623400 -2.64424200 1.76489000

H 4.04978800 0.06816200 4.41123200

H 6.09993400 -1.19860700 3.78778900

H -0.02599200 -3.42335100 1.60291200

H 0.02737700 -1.91525200 2.56246300

Coordinates of **2** fragment loss of (C^C).

Au -0.87786100 -0.22311500 2.02595300

P 1.71177300 -0.75223400 0.43564000

P -1.19333800 -0.22205100 -0.30099800

C 0.35541600 -1.02747400 -0.84507800

C -1.27005100 1.43100700 -1.02995900

C -1.23576800 1.54977300 -2.42812400

C -1.33442900 2.57544800 -0.23130300

C -1.26048600 2.81007300 -3.01399500

H -1.19902100 0.65751300 -3.06393000

C -1.35577700 3.83495200 -0.82596300

H -1.35777700 2.48470400 0.86046100

C -1.31764900 3.95143200 -2.21289000

H -1.23813200 2.90302800 -4.10293900

H -1.40303700 4.72915600 -0.19922400

H -1.33753900 4.94106900 -2.67723300

C -2.60521100 -1.19298600 -0.87789200

C -3.71376500 -0.56773900 -1.46181000

C -2.61208200 -2.57952800 -0.66667700

C -4.80640600 -1.33416800 -1.85899000

H -3.72585200 0.51616300 -1.61212200

C -3.70606800 -3.33573000 -1.06892600

H -1.76656900 -3.07631100 -0.17676100

C -4.80202800 -2.71361500 -1.66784000

H -5.66832400 -0.84656900 -2.32150700

H -3.70713000 -4.41697800 -0.90977700

H -5.66219500 -3.31048600 -1.98204100

C 2.00688900 1.05132100 0.24801400

C 2.08617200 1.69483800 -0.99501600

C 2.13843500 1.81316300 1.41575700

C 2.27354500 3.07126200 -1.06504800

H 2.01145800 1.11731800 -1.92378100

C 2.33418500 3.19239400 1.34606700

H 2.09892100 1.31783700 2.39350600

C 2.39575900 3.82141800 0.10584500

H 2.32707800 3.56342400 -2.04044900

H 2.44390200 3.77493100 2.26478400

H 2.54839900 4.90276200 0.04818100

C 3.13502500 -1.54320200 -0.40870200

C 4.29728900 -0.84905900 -0.76232200

C 3.07749800 -2.93041300 -0.60951200

C 5.37019600 -1.52988600 -1.33512800

H 4.37227000 0.22933200 -0.59100700

C 4.14515500 -3.60337400 -1.19270200

H 2.19199900 -3.49773100 -0.29719600

C 5.29415300 -2.90205800 -1.55763800

H 6.27430800 -0.97951700 -1.60939600

H 4.08648700 -4.68334100 -1.35271500

H 6.13752900 -3.43121500 -2.00915100

H 0.63271600 -0.66820500 -1.85175400

H 0.17502700 -2.11359400 -0.91691000

Coordinates of **3.**

Au -0.00015600 0.16806200 0.00052100

C -1.34229200 1.75213900 0.05039800

C -0.73569400 3.02458200 0.02738700

C -2.71204100 1.61706400 0.20511100

C -1.56331200 4.14077400 0.12455600

C 0.72335400 3.02666900 -0.07691800

C -3.55119200 2.74211000 0.31204000

H -3.17109500 0.62381200 0.25393000

C -2.94595900 3.99981900 0.25875900

H -1.12821000 5.14549400 0.10856600

C 1.33530100 1.75651800 -0.07495100

C 1.54634800 4.14442300 -0.19307800

H -3.55322700 4.90487100 0.33297500

C 2.70613700 1.62467500 -0.22243000

C 2.93015400 4.00706400 -0.31884500

H 1.10682200 5.14733400 -0.19767100

C 3.54105400 2.75127700 -0.34503900

H 3.16936700 0.63251100 -0.25136300

H 3.53401800 4.91310900 -0.40732600

C 5.04448100 2.54130900 -0.50596100

C 5.58092700 1.74644600 0.68972200

C 5.30611500 1.76009000 -1.79839000

C 5.79996200 3.86417300 -0.57974800

H 5.37898400 2.27266000 1.63862400

H 5.13365100 0.73989600 0.76155400

H 6.67274100 1.61432200 0.60212800

H 4.93373400 2.31412000 -2.67630200

H 6.38896800 1.59672400 -1.93433200

H 4.81801100 0.77005100 -1.79424900

H 6.87943000 3.67261100 -0.69275900

H 5.48381900 4.47211100 -1.44353500

H 5.66648300 4.46659700 0.33431700

C -5.05182700 2.52896700 0.49418800

C -5.59685400 1.68414900 -0.66263600

C -5.29360100 1.79636100 1.81865200

C -5.81525600 3.84896500 0.52480200

H -5.40347800 2.16932800 -1.63481000

H -5.15109500 0.67487700 -0.69371800

H -6.68781600 1.55579000 -0.56019800

H -4.91740800 2.38700200 2.67068400

H -6.37351200 1.62858500 1.97208100

H -4.79555700 0.81160000 1.84664000

H -6.89271100 3.65503200 0.65201000

H -5.49805000 4.48995500 1.36388500

H -5.69096300 4.41851800 -0.41141900

P -1.62165900 -1.68363500 -0.28432500

P 1.62647800 -1.67674800 0.30861800

C 0.61156000 -3.22539800 0.48147200

C -0.60520800 -3.23410000 -0.42733300

C 2.61278000 -1.63508800 1.83533500

C 3.23559200 -2.79981700 2.31170200

C 2.73089700 -0.44291800 2.56066200

C 3.97520300 -2.76416300 3.48903200

H 3.14986300 -3.74391000 1.76217100

C 3.47435600 -0.41491200 3.73910200

H 2.24685500 0.47195400 2.20202400

C 4.09655900 -1.57102400 4.20142500

H 4.45971000 -3.67372500 3.85379200

H 3.56395300 0.51976400 4.29882800

H 4.67866700 -1.54604800 5.12655200

C 2.74239400 -1.91663900 -1.10935700

C 4.08699800 -2.27647800 -0.96121000

C 2.22523900 -1.68003600 -2.39172300

C 4.89477500 -2.42168500 -2.08724400

H 4.51379600 -2.43229800 0.03471000

C 3.03524900 -1.83561800 -3.51204100

H 1.18609800 -1.34930900 -2.51955400

C 4.37001500 -2.20847800 -3.35988800

H 5.94507700 -2.69981500 -1.96652000

H 2.62626800 -1.65010800 -4.50853600

H 5.00870900 -2.32118400 -4.23992000

C -2.74924400 -1.89880700 1.12800400

C -4.10051500 -2.23004300 0.97412700

C -2.23462100 -1.66431600 2.41182500

C -4.91781000 -2.34974800 2.09643700

H -4.52480500 -2.38421700 -0.02316500

C -3.05413300 -1.79501800 3.52831900

H -1.18978200 -1.35375300 2.54362000

C -4.39589200 -2.13971400 3.37071500

H -5.97322400 -2.60574200 1.97133200

H -2.64702100 -1.61169000 4.52598500

H -5.04193100 -2.23265500 4.24768300

C -2.59521500 -1.66389200 -1.81916300

C -3.22332100 -2.83277500 -2.27790900

C -2.69617400 -0.48547200 -2.56891500

C -3.95099700 -2.81462600 -3.46306800

H -3.15114800 -3.76592200 -1.70800900

C -3.42752800 -0.47502700 -3.75521200

H -2.20883800 0.43249800 -2.22275200

C -4.05480400 -1.63508000 -4.20049000

H -4.43989300 -3.72715100 -3.81432100

H -3.50380300 0.44882400 -4.33447800

H -4.62745600 -1.62388300 -5.13176900

H -0.30524800 -3.29331100 -1.48983500

H 1.24796000 -4.10701000 0.29204700

H 0.31251000 -3.26661000 1.54502900

H -1.24092100 -4.11298200 -0.22349700

Coordinates of **3** fragment loss of (C^C).

Au -0.00001000 0.00000900 -1.54910200

P -1.80980500 0.04794300 0.07706400

P 1.80978100 -0.04796800 0.07706300

C 0.65882200 0.39097300 1.47832800

C -0.65884200 -0.39108000 1.47830000

C 3.23619300 1.05986500 0.23846700

C 3.28898400 2.11268200 1.15901300

C 4.31985000 0.83427400 -0.62266400

C 4.41983600 2.92532300 1.21865500

H 2.45773900 2.31058800 1.84166300

C 5.44606900 1.64508200 -0.55296400

H 4.28210900 0.01380100 -1.34876800

C 5.49608200 2.69274800 0.36713000

H 4.45862100 3.74471700 1.94124200

H 6.28995700 1.46158500 -1.22306200

H 6.38115500 3.33226000 0.41871600

C 2.39987400 -1.71868000 0.49136200

C 3.28542900 -1.90536700 1.56220800

C 1.94421200 -2.82370700 -0.23522100

C 3.69902600 -3.18875900 1.90274300

H 3.66128200 -1.04333000 2.12571900

C 2.35903300 -4.10761800 0.11271100

H 1.26856500 -2.67990500 -1.08754400

C 3.23504500 -4.28842700 1.17996000

H 4.39256300 -3.33311500 2.73537500

H 2.00386600 -4.96866300 -0.45936500

H 3.56654000 -5.29511400 1.44831300

C -2.39981900 1.71867000 0.49140100

C -3.28503400 1.90540800 1.56252200

C -1.94446200 2.82365200 -0.23543700

C -3.69859000 3.18880700 1.90307600

H -3.66065800 1.04340200 2.12623600

C -2.35925100 4.10757200 0.11250500

H -1.26907800 2.67980600 -1.08796100

C -3.23491500 4.28843200 1.18003100

H -4.39185700 3.33320200 2.73592600

H -2.00432500 4.96858300 -0.45977100

H -3.56637700 5.29512600 1.44839800

C -3.23625800 -1.05983900 0.23848600

C -3.28885800 -2.11295300 1.15870300

C -4.32010500 -0.83395600 -0.62232700

C -4.41970500 -2.92560000 1.21833700

H -2.45746200 -2.31109400 1.84110000

C -5.44631800 -1.64477600 -0.55263500

H -4.28252000 -0.01325200 -1.34817700

C -5.49613900 -2.69273900 0.36712900

H -4.45833800 -3.74522600 1.94066800

H -6.29035200 -1.46104900 -1.22248600

H -6.38120600 -3.33226100 0.41870400

H -0.46128700 -1.47777900 1.41343200

H 1.19594300 0.21763200 2.42817400

H 0.46128100 1.47768000 1.41352800

H -1.19596800 -0.21782000 2.42815800

Coordinates of **4.**

Au -0.02258100 0.12043200 0.19243100

C 1.34609800 1.66589800 -0.18411700

C 0.74950500 2.93125500 -0.34638200

C 2.71627500 1.51700500 -0.32553200

C 1.57804300 4.02386600 -0.59571400

C -0.70696500 2.96400900 -0.28226800

C 3.55804500 2.61387300 -0.58793000

H 3.18822500 0.53563800 -0.23635100

C 2.95880300 3.86909500 -0.70622500

H 1.14111500 5.01989900 -0.71967200

C -1.34736100 1.72621600 -0.06854100

C -1.49956000 4.09683500 -0.45335400

H 3.56691900 4.75510500 -0.90200500

C -2.73150400 1.65343400 -0.03727100

C -2.89036900 4.01189300 -0.43040600

H -1.02687800 5.07000000 -0.62016700

C -3.53805500 2.79264300 -0.22792500

H -3.23776900 0.70122100 0.13541400

H -3.46935100 4.92578800 -0.58079900

C -5.05742700 2.64903300 -0.18164400

C -5.50616100 1.52853000 -1.12556300

C -5.47489600 2.31316700 1.25353800

C -5.76466500 3.93386300 -0.60261600

H -5.18847700 1.73206900 -2.16276300

H -5.10483400 0.54270900 -0.83537900

H -6.60615300 1.44486400 -1.12008800

H -5.20168000 3.12994300 1.94253200

H -6.56769700 2.16783900 1.31426400

H -4.99133900 1.39240200 1.61982300

H -6.85623900 3.78327000 -0.57932500

H -5.54005800 4.77283000 0.07618200

H -5.49248500 4.23638500 -1.62778900

C 5.05646800 2.37353900 -0.75425400

C 5.61097600 1.65481600 0.48043700

C 5.27888100 1.50734000 -1.99883700

C 5.82697200 3.67820200 -0.93053700

H 5.44868700 2.25319900 1.39331400

H 5.14645600 0.66635200 0.64285100

H 6.69666600 1.49252900 0.37089200

H 4.90489600 2.01570100 -2.90332600

H 6.35503300 1.30794800 -2.14149900

H 4.76351600 0.53451300 -1.92501200

H 6.90300200 3.46489300 -1.03741800

H 5.51246200 4.22527800 -1.83453300

H 5.70732600 4.34764500 -0.06223600

P 1.78403200 -1.58942100 0.59899100

P -1.77776300 -1.67609200 0.28549600

C -1.22837300 -3.30326400 0.95159200

C 1.30744800 -3.32045400 1.05480800

C -2.34097400 -2.01637200 -1.41953400

C -2.77822300 -3.29005600 -1.81193200

C -2.35375800 -0.97233300 -2.35630700

C -3.22532600 -3.51072400 -3.11140900

H -2.77974500 -4.12644400 -1.10603000

C -2.80720100 -1.19793600 -3.65379100

H -2.01646500 0.03071900 -2.07221400

C -3.24247300 -2.46517500 -4.03284200

H -3.56399400 -4.50778700 -3.40527100

H -2.81698300 -0.37440200 -4.37262000

H -3.59521900 -2.64140200 -5.05256700

C -3.25732800 -1.39535200 1.31686400

C -4.53585900 -1.78418800 0.90463500

C -3.07101700 -0.84222200 2.58994800

C -5.61822300 -1.62891100 1.76969000

H -4.69335200 -2.20380900 -0.09509200

C -4.15432200 -0.69664900 3.45055800

H -2.07358600 -0.51190800 2.90638800

C -5.42753600 -1.09278900 3.04090800

H -6.61780200 -1.93086700 1.44568200

H -4.00647700 -0.26472600 4.44376600

H -6.27896200 -0.97357300 3.71625500

C 2.72494300 -1.82969600 -0.95223200

C 4.08250600 -2.17155400 -0.97499200

C 2.02714800 -1.70228900 -2.16062600

C 4.72523400 -2.39444800 -2.19021800

H 4.65481300 -2.24751900 -0.04536200

C 2.67431400 -1.92918700 -3.37262200

H 0.96935400 -1.40911300 -2.16303000

C 4.02282000 -2.27739800 -3.38812200

H 5.78691900 -2.65518900 -2.20001100

H 2.12172900 -1.82463100 -4.30990300

H 4.53239300 -2.44997800 -4.33977600

C 2.91640000 -1.14424500 1.96254600

C 3.89296900 -2.04667200 2.41455600

C 2.77080900 0.08514500 2.61708200

C 4.72770700 -1.70551200 3.47416300

H 4.00655800 -3.03124400 1.94927200

C 3.60209900 0.41770400 3.68382500

H 2.00796700 0.79771400 2.28805400

C 4.58608000 -0.47122400 4.10689900

H 5.48930300 -2.41281000 3.81303900

H 3.47758600 1.38100400 4.18521700

H 5.24140200 -0.20716100 4.94122000

C 0.06267800 -3.87576100 0.38241300

H 2.18571000 -3.94518300 0.81653600

H 1.19170600 -3.33875800 2.15349200

H -2.07847900 -3.99442200 0.81951300

H -1.14611500 -3.15836400 2.04450100

H 0.11200100 -3.73906500 -0.71522900

H 0.05439200 -4.96827200 0.53358600

Coordinates of **4** fragment loss of (C^C).

Au -0.00174100 -0.60341600 -0.95516600

P -2.03732600 -0.22550900 0.22361800

P 2.04699500 -0.27006900 0.21081500

C 1.31535000 -0.45464200 1.91145400

C -1.31970900 -0.44013600 1.92707500

C 2.61741200 1.44969700 0.07378600

C 2.97829800 2.21200900 1.19211800

C 2.72127000 2.00959600 -1.20659500

C 3.43685200 3.51653400 1.02731600

H 2.90962700 1.79413700 2.20178700

C 3.18707700 3.31137500 -1.36653800

H 2.43867700 1.41864300 -2.08588400

C 3.54269900 4.06517600 -0.24945000

H 3.71579000 4.10791700 1.90342500

H 3.27091000 3.74026600 -2.36851200

H 3.90444900 5.08930100 -0.37427400

C 3.50605500 -1.34957900 0.23626800

C 4.79795300 -0.84562200 0.42057800

C 3.30631500 -2.72775300 0.08387000

C 5.87948900 -1.72292500 0.46427600

H 4.96480600 0.23151700 0.52511800

C 4.38986700 -3.59754200 0.13690600

H 2.29681800 -3.12263500 -0.08580200

C 5.67660100 -3.09403200 0.32652200

H 6.88969200 -1.32971400 0.60541500

H 4.23227500 -4.67276000 0.01887400

H 6.52942300 -3.77723600 0.35964600

C -2.56703400 1.51651400 0.17906400

C -3.75001900 1.90388100 0.82270100

C -1.77014700 2.48056000 -0.44914900

C -4.12428400 3.24370500 0.83905500

H -4.38685700 1.15381000 1.30587700

C -2.14742300 3.82112300 -0.42468800

H -0.84927900 2.18391200 -0.96741600

C -3.32342700 4.20140100 0.21748600

H -5.05025700 3.54274900 1.33729300

H -1.52297100 4.57057600 -0.91794900

H -3.62313700 5.25276100 0.22914800

C -3.54974000 -1.22588600 0.17459800

C -3.99901000 -1.98710900 1.25921300

C -4.28219700 -1.21961500 -1.02093500

C -5.17201400 -2.73149500 1.14563000

H -3.44709800 -2.00858600 2.20320900

C -5.45421400 -1.95892300 -1.12490300

H -3.93211400 -0.62774800 -1.87492800

C -5.89867200 -2.71747200 -0.04160000

H -5.51999000 -3.32496800 1.99517700

H -6.02315500 -1.94785400 -2.05818500

H -6.81802100 -3.30293700 -0.12577200

C 0.00357800 0.28693900 2.22008400

H -2.09561900 -0.08897900 2.63195900

H -1.20754900 -1.52733000 2.09510500

H 2.10209500 -0.16155900 2.62975300

H 1.19587500 -1.54519700 2.05079800

H 0.00291900 1.29336000 1.75840900

H 0.00966200 0.48742900 3.30446600

Coordinates of **5.**

Au -0.00350700 0.11279400 0.04475900

C -1.30856500 1.75199000 0.02273500

C -0.65100100 2.99565600 -0.06878900

C -2.69085200 1.68392300 -0.04024300

C -1.42740100 4.14049400 -0.23444400

C 0.80354900 2.95752800 0.04126900

C -3.48092000 2.83728100 -0.20872400

H -3.20809200 0.72525600 0.04612000

C -2.81731400 4.06133300 -0.30743000

H -0.94295700 5.11903800 -0.31406400

C 1.37852600 1.68122600 0.15033900

C 1.64950400 4.06755500 0.10011200

H -3.38354000 4.98560100 -0.44272200

C 2.74164100 1.53275900 0.37874600

C 3.01496900 3.91046300 0.30442000

H 1.23301700 5.07557200 0.00580400

C 3.59185000 2.64555300 0.47505000

H 3.17945300 0.53964900 0.49224600

H 3.64279600 4.80561300 0.34746300

C 5.09365600 2.50352100 0.71638800

C 5.47809400 1.08748700 1.14196600

C 5.82691300 2.83815000 -0.58666900

C 5.54390200 3.46284200 1.82161300

H 4.93936200 0.76936300 2.05190500

H 5.28254800 0.34650900 0.34853500

H 6.55727300 1.04565700 1.36204200

H 5.61921300 3.86924100 -0.91661100

H 6.91832700 2.74036000 -0.45426400

H 5.51418100 2.15741300 -1.39757600

H 6.62437200 3.34521300 2.00904200

H 5.37549100 4.51904800 1.55974700

H 5.01141200 3.26026800 2.76608000

C -5.00206500 2.70657700 -0.22465700

C -5.43500600 1.61203400 -1.20572600

C -5.46793300 2.34520300 1.18976300

C -5.68114100 4.00818700 -0.64128000

H -5.08114000 1.83328800 -2.22753300

H -5.05904200 0.61167400 -0.92860100

H -6.53556800 1.54550400 -1.23721800

H -5.19291600 3.13654000 1.90707600

H -6.56510900 2.22666400 1.21756800

H -5.01454900 1.40353100 1.54292800

H -6.77395500 3.86743100 -0.66809600

H -5.47944500 4.82722900 0.06815700

H -5.36222800 4.33490000 -1.64541800

P -1.79725600 -1.61906800 0.28637200

P 1.73979500 -1.60396900 -0.48617100

C 2.77173600 -2.14733200 0.91113700

C 3.74543000 -3.14651600 0.76216100

C 2.57333200 -1.56566300 2.16970400

C 4.52235100 -3.53363200 1.84941000

H 3.90104400 -3.63266100 -0.20746900

C 3.35086000 -1.95886600 3.25693800

H 1.81241000 -0.78612000 2.29937600

C 4.32858600 -2.93691200 3.09519300

H 5.28258200 -4.30933600 1.72507000

H 3.19200100 -1.49636100 4.23452700

H 4.94115900 -3.24338000 3.94729400

C 2.81394000 -0.87767200 -1.77695100

C 4.20634700 -0.99697900 -1.78297800

C 2.17688600 -0.18542600 -2.81433100

C 4.95116800 -0.41854300 -2.80949900

H 4.72464400 -1.52535500 -0.97665200

C 2.92341800 0.38716400 -3.83868800

H 1.08498600 -0.07158000 -2.81544400

C 4.31291900 0.27518500 -3.83439500

H 6.04120800 -0.50741400 -2.80179400

H 2.41819600 0.93263500 -4.63990400

H 4.90076300 0.73303000 -4.63438800

C -2.87653800 -1.14866700 1.68929100

C -4.25367200 -1.39391800 1.70992100

C -2.25918600 -0.58517900 2.81247400

C -4.99839200 -1.08650500 2.84682900

H -4.76023500 -1.81072900 0.83376800

C -3.00803600 -0.27743800 3.94451100

H -1.18315900 -0.36919400 2.80111600

C -4.37823800 -0.53103100 3.96352200

H -6.07515600 -1.27606600 2.85471100

H -2.51892900 0.16775700 4.81478800

H -4.96710200 -0.28697500 4.85163000

C -2.82949200 -1.86233100 -1.19430900

C -3.77783500 -2.89437100 -1.26994400

C -2.66421400 -1.00363900 -2.28865600

C -4.56204400 -3.04319600 -2.40934300

H -3.90845200 -3.59455800 -0.43745900

C -3.44832600 -1.15965300 -3.42989100

H -1.92773100 -0.19241500 -2.24414000

C -4.40071900 -2.17332800 -3.48770800

H -5.30218000 -3.84629300 -2.45807200

H -3.31561800 -0.48070600 -4.27623500

H -5.01868600 -2.29287900 -4.38162300

C 1.20720000 -3.15768300 -1.34941000

H 0.47667400 -2.84609800 -2.12113600

C -1.30223300 -3.32079900 0.84065500

H -0.56738700 -3.17245300 1.65571600

H -2.20731700 -3.71338300 1.33708700

C 0.68083100 -4.31839700 -0.51390700

H 0.89431300 -5.24007900 -1.08167100

H 1.26749900 -4.41662900 0.41884100

C -0.80277600 -4.32532200 -0.19167700

H -1.38693000 -4.23642800 -1.12667000

H -1.04564300 -5.32770600 0.20044400

H 2.10481900 -3.47302000 -1.91030800

Coordinates of **5** fragment loss of (C^C).

Au -0.01033100 -0.09135500 -0.52432600

P -2.24843000 0.07785900 0.28386600

P 2.22573800 -0.13149900 0.28989200

C 3.06215900 1.47661600 0.13958100

C 3.40020100 2.27090800 1.24013100

C 3.37447700 1.91713100 -1.15440400

C 4.04851400 3.48992900 1.04571200

H 3.16398100 1.95178900 2.26000100

C 4.02877400 3.12906900 -1.34152600

H 3.10847700 1.30092000 -2.02174200

C 4.36485700 3.91738800 -0.24063400

H 4.31054400 4.10698200 1.90926100

H 4.27573200 3.46347100 -2.35251800

H 4.87561300 4.87258200 -0.38846700

C 3.44197800 -1.39902400 -0.16218900

C 4.79513900 -1.22862000 0.15687400

C 3.01158500 -2.57602300 -0.78403700

C 5.70669300 -2.23626700 -0.14222300

H 5.13908400 -0.30351300 0.63321200

C 3.92887700 -3.58128800 -1.07836500

H 1.95451200 -2.70333900 -1.04559600

C 5.27403900 -3.41040600 -0.75780200

H 6.76349800 -2.10349900 0.10386600

H 3.59327900 -4.49936900 -1.56758500

H 5.99459700 -4.19773700 -0.99493100

C -3.31877900 1.46057900 -0.21112700

C -4.59692500 1.26602800 -0.74357000

C -2.80100000 2.75899100 -0.10163800

C -5.35663300 2.36528200 -1.13988500

H -5.00445300 0.25617400 -0.85292400

C -3.56644600 3.85107000 -0.49339600

H -1.78678300 2.92064100 0.28532200

C -4.84666200 3.65419500 -1.01124900

H -6.35649700 2.20983300 -1.55358000

H -3.16035300 4.86176300 -0.40109300

H -5.44733600 4.51296600 -1.32233300

C -3.21387500 -1.45736600 0.17839800

C -4.32886500 -1.66274000 1.00435400

C -2.84679100 -2.44531700 -0.74188200

C -5.05867300 -2.84248300 0.91024400

H -4.64259700 -0.89309900 1.71867500

C -3.58075300 -3.62631200 -0.83294100

H -1.98094000 -2.28794400 -1.39543100

C -4.68333100 -3.82487000 -0.00657600

H -5.92754000 -2.99707200 1.55535600

H -3.28869400 -4.39432100 -1.55377400

H -5.25817800 -4.75218200 -0.07696200

C 1.87643000 -0.38530500 2.08947800

H 1.75918900 -1.47632200 2.21603200

C -1.91019800 0.32848800 2.09687100

H -1.81418800 1.41553500 2.26338400

H -2.82427000 0.02475800 2.63412700

C 0.64084300 0.37504100 2.59827800

H 0.84952600 0.73168200 3.62131000

H 0.51219900 1.30584400 2.01119900

C -0.67854300 -0.42711900 2.61402000

H -0.56523300 -1.37527500 2.05270100

H -0.88225800 -0.75178500 3.64782100

H 2.79303600 -0.12329300 2.64709700

Coordinates of **6.**

Au 0.00970100 -0.19488100 -0.12713200

C -1.36105400 -1.76998200 0.00391800

C -0.75556400 -3.03668800 0.13870600

C -2.73498700 -1.63298300 0.13623700

C -1.57349400 -4.13737700 0.37813300

C 0.69698900 -3.06653700 0.00916700

C -3.56768900 -2.74353600 0.38724000

H -3.20195500 -0.64785800 0.04815900

C -2.95418200 -3.99241100 0.49749700

H -1.12920500 -5.13193300 0.48710700

C 1.31884700 -1.81502400 -0.15553100

C 1.49823600 -4.20536800 -0.01812000

H -3.55304300 -4.88508200 0.68899900

C 2.67807500 -1.72811500 -0.41143300

C 2.87284700 -4.10976200 -0.23241700

H 1.04538700 -5.19384300 0.10984600

C 3.49213400 -2.87807700 -0.45260700

H 3.14090400 -0.75937500 -0.61482000

H 3.45986800 -5.03056400 -0.24499100

C 4.98574400 -2.73200800 -0.73814300

C 5.17794000 -2.11734800 -2.12805300

C 5.62505500 -1.82387300 0.31759600

C 5.70665100 -4.07622700 -0.70500600

H 4.73134600 -2.75620800 -2.90787800

H 4.71808300 -1.11820800 -2.21078300

H 6.25216200 -2.00701000 -2.35393000

H 5.46797300 -2.22306200 1.33406500

H 6.71232000 -1.74492500 0.14892000

H 5.21861900 -0.79805500 0.28734900

H 6.77828300 -3.92988600 -0.91617100

H 5.62909200 -4.56210300 0.28183700

H 5.31594500 -4.77233500 -1.46526200

C -5.07609200 -2.54495800 0.52778900

C -5.80230500 -3.85617000 0.81393800

C -5.35800800 -1.58417200 1.68729200

C -5.63871200 -1.96820400 -0.77494500

H -5.67146400 -4.58725800 -0.00082000

H -5.46198400 -4.32025800 1.75428800

H -6.88350900 -3.66787300 0.91389900

H -4.89548700 -0.59424400 1.53625300

H -6.44403300 -1.42708700 1.80102100

H -4.97313100 -1.98997700 2.63769400

H -6.73143000 -1.83761600 -0.69867100

H -5.20782700 -0.98240900 -1.01819200

H -5.43563200 -2.63998300 -1.62565600

C 0.90730400 3.22065700 -0.09170300

C -0.43631200 3.28216800 -0.51207900

C 1.69630400 4.37806300 -0.14157000

C -0.94687700 4.50033200 -0.98008800

C -0.15187400 5.64008000 -1.02945500

H 2.74108300 4.34615200 0.17806000

C 1.17249200 5.57980300 -0.60577800

H -1.98250000 4.56901700 -1.32494500

H -0.56917400 6.57770000 -1.40526300

H 1.80488100 6.47045600 -0.64256600

C -2.50770000 1.87852000 -2.05128000

C -2.92704600 2.06036100 0.70630500

C -3.83012800 2.50451000 -1.58450100

H -1.95700500 2.56096500 -2.72200500

C -4.16684400 1.96499500 -0.19948500

H -2.89961200 1.20726900 1.40861800

H -3.74689200 3.60530200 -1.54770500

H -4.63250200 2.28624600 -2.30987900

H -5.01330100 2.50508100 0.25722300

H -4.49179600 0.91406600 -0.28802000

C 1.56929800 1.76272600 2.36321300

C 3.42659700 1.64636600 0.45038700

C 2.71476800 2.74647200 2.63070200

H 0.59793600 2.19138300 2.67138900

C 3.88554000 2.48112900 1.66461600

H 3.63134600 0.58586300 0.68748600

H 2.34510500 3.77729400 2.49844400

H 3.03200400 2.67489800 3.68462900

H 4.32841300 3.43320200 1.32341700

H 4.70595300 1.94434900 2.16973100

C -2.68456100 0.54108700 -2.75243900

H -1.71965500 0.07605800 -3.00941700

H -3.24447500 -0.18569500 -2.14267300

H -3.24518800 0.68757300 -3.68935000

C -2.85223400 3.35193400 1.50293400

H -2.95610800 4.24452000 0.86539200

H -3.67285000 3.37768600 2.23763900

H -1.90655000 3.45141600 2.05963700

C 4.08195400 1.95785300 -0.88033000

H 3.90290600 2.99411000 -1.20838100

H 5.17356600 1.82166500 -0.80543800

H 3.72164600 1.29013200 -1.68089400

C 1.77001300 0.40566200 3.02305100

H 0.95042200 -0.29669800 2.80000600

H 2.71128900 -0.07819100 2.70908300

H 1.81812100 0.52731400 4.11699600

P 1.57500500 1.62226900 0.50544800

P -1.49377000 1.77374600 -0.47762000

Coordinates of **6** fragment loss of (C^C).

Au -0.14008400 -1.64638100 -0.35169300

C 0.83265100 1.40603500 -0.28951600

C -0.58843100 1.51636100 -0.31943700

C 1.59324700 2.56463800 -0.51266100

C -1.15965800 2.77104800 -0.57493600

C -0.38288200 3.90287500 -0.79349900

H 2.68446300 2.50785700 -0.50007700

C 1.00252700 3.79902800 -0.75946500

H -2.24871300 2.87729000 -0.59989300

H -0.86385100 4.86462200 -0.98874200

H 1.62982800 4.67800900 -0.92766800

C -3.37404900 0.45950400 -0.88919200

C -2.41316400 0.25716900 1.72967800

C -4.29966400 0.90870400 0.24959300

H -3.20682000 1.26257300 -1.62680000

C -3.92677400 0.13154500 1.50839300

H -2.02967600 -0.61487600 2.28692400

H -4.18947300 1.99284300 0.43096100

H -5.35493300 0.74937100 -0.02924000

H -4.47281900 0.49419500 2.39533400

H -4.20338400 -0.93096400 1.38775100

C 2.40319300 -0.09299100 1.73148700

C 3.44011300 -0.10846200 -0.75466200

C 3.58811000 0.87112800 1.59534600

H 1.61530100 0.33238700 2.37893800

C 4.33406500 0.62587600 0.26963500

H 3.69508300 -1.18536500 -0.72532500

H 3.21566600 1.90848400 1.63292500

H 4.25958300 0.76400900 2.46390600

H 4.68532900 1.58233400 -0.15568300

H 5.24675500 0.03014000 0.43561100

C -3.87473600 -0.78362600 -1.61050900

H -3.16854000 -1.12189100 -2.38507000

H -4.03489300 -1.62618100 -0.91672300

H -4.83746000 -0.57238200 -2.10259000

C -2.01071700 1.52893400 2.45515300

H -2.40625000 2.43607500 1.97038100

H -2.40830000 1.50614600 3.48277700

H -0.91731700 1.64576200 2.52430900

C 3.54328300 0.34156400 -2.19900100

H 3.27592700 1.40131200 -2.33348200

H 4.57743200 0.21771500 -2.55757200

H 2.89441600 -0.25586000 -2.85907100

C 2.80214600 -1.47189300 2.23367500

H 1.94469300 -2.16243400 2.27829800

H 3.57564700 -1.93739100 1.59927100

H 3.22290700 -1.39610900 3.24910400

P 1.74236300 -0.18383700 -0.00440400

P -1.75366600 0.10728800 -0.02528300

Coordinates of **7.**

Au 0.00970100 -0.19488100 -0.12713200

C -1.36105400 -1.76998200 0.00391800

C -0.75556400 -3.03668800 0.13870600

C -2.73498700 -1.63298300 0.13623700

C -1.57349400 -4.13737700 0.37813300

C 0.69698900 -3.06653700 0.00916700

C -3.56768900 -2.74353600 0.38724000

H -3.20195500 -0.64785800 0.04815900

C -2.95418200 -3.99241100 0.49749700

H -1.12920500 -5.13193300 0.48710700

C 1.31884700 -1.81502400 -0.15553100

C 1.49823600 -4.20536800 -0.01812000

H -3.55304300 -4.88508200 0.68899900

C 2.67807500 -1.72811500 -0.41143300

C 2.87284700 -4.10976200 -0.23241700

H 1.04538700 -5.19384300 0.10984600

C 3.49213400 -2.87807700 -0.45260700

H 3.14090400 -0.75937500 -0.61482000

H 3.45986800 -5.03056400 -0.24499100

C 4.98574400 -2.73200800 -0.73814300

C 5.17794000 -2.11734800 -2.12805300

C 5.62505500 -1.82387300 0.31759600

C 5.70665100 -4.07622700 -0.70500600

H 4.73134600 -2.75620800 -2.90787800

H 4.71808300 -1.11820800 -2.21078300

H 6.25216200 -2.00701000 -2.35393000

H 5.46797300 -2.22306200 1.33406500

H 6.71232000 -1.74492500 0.14892000

H 5.21861900 -0.79805500 0.28734900

H 6.77828300 -3.92988600 -0.91617100

H 5.62909200 -4.56210300 0.28183700

H 5.31594500 -4.77233500 -1.46526200

C -5.07609200 -2.54495800 0.52778900

C -5.80230500 -3.85617000 0.81393800

C -5.35800800 -1.58417200 1.68729200

C -5.63871200 -1.96820400 -0.77494500

H -5.67146400 -4.58725800 -0.00082000

H -5.46198400 -4.32025800 1.75428800

H -6.88350900 -3.66787300 0.91389900

H -4.89548700 -0.59424400 1.53625300

H -6.44403300 -1.42708700 1.80102100

H -4.97313100 -1.98997700 2.63769400

H -6.73143000 -1.83761600 -0.69867100

H -5.20782700 -0.98240900 -1.01819200

H -5.43563200 -2.63998300 -1.62565600

C 0.90730400 3.22065700 -0.09170300

C -0.43631200 3.28216800 -0.51207900

C 1.69630400 4.37806300 -0.14157000

C -0.94687700 4.50033200 -0.98008800

C -0.15187400 5.64008000 -1.02945500

H 2.74108300 4.34615200 0.17806000

C 1.17249200 5.57980300 -0.60577800

H -1.98250000 4.56901700 -1.32494500

H -0.56917400 6.57770000 -1.40526300

H 1.80488100 6.47045600 -0.64256600

C -2.50770000 1.87852000 -2.05128000

C -2.92704600 2.06036100 0.70630500

C -3.83012800 2.50451000 -1.58450100

H -1.95700500 2.56096500 -2.72200500

C -4.16684400 1.96499500 -0.19948500

H -2.89961200 1.20726900 1.40861800

H -3.74689200 3.60530200 -1.54770500

H -4.63250200 2.28624600 -2.30987900

H -5.01330100 2.50508100 0.25722300

H -4.49179600 0.91406600 -0.28802000

C 1.56929800 1.76272600 2.36321300

C 3.42659700 1.64636600 0.45038700

C 2.71476800 2.74647200 2.63070200

H 0.59793600 2.19138300 2.67138900

C 3.88554000 2.48112900 1.66461600

H 3.63134600 0.58586300 0.68748600

H 2.34510500 3.77729400 2.49844400

H 3.03200400 2.67489800 3.68462900

H 4.32841300 3.43320200 1.32341700

H 4.70595300 1.94434900 2.16973100

C -2.68456100 0.54108700 -2.75243900

H -1.71965500 0.07605800 -3.00941700

H -3.24447500 -0.18569500 -2.14267300

H -3.24518800 0.68757300 -3.68935000

C -2.85223400 3.35193400 1.50293400

H -2.95610800 4.24452000 0.86539200

H -3.67285000 3.37768600 2.23763900

H -1.90655000 3.45141600 2.05963700

C 4.08195400 1.95785300 -0.88033000

H 3.90290600 2.99411000 -1.20838100

H 5.17356600 1.82166500 -0.80543800

H 3.72164600 1.29013200 -1.68089400

C 1.77001300 0.40566200 3.02305100

H 0.95042200 -0.29669800 2.80000600

H 2.71128900 -0.07819100 2.70908300

H 1.81812100 0.52731400 4.11699600

P 1.57500500 1.62226900 0.50544800

P -1.49377000 1.77374600 -0.47762000

Coordinates of **7** fragment loss of (C^C).

Au -0.14008400 -1.64638100 -0.35169300

C 0.83265100 1.40603500 -0.28951600

C -0.58843100 1.51636100 -0.31943700

C 1.59324700 2.56463800 -0.51266100

C -1.15965800 2.77104800 -0.57493600

C -0.38288200 3.90287500 -0.79349900

H 2.68446300 2.50785700 -0.50007700

C 1.00252700 3.79902800 -0.75946500

H -2.24871300 2.87729000 -0.59989300

H -0.86385100 4.86462200 -0.98874200

H 1.62982800 4.67800900 -0.92766800

C -3.37404900 0.45950400 -0.88919200

C -2.41316400 0.25716900 1.72967800

C -4.29966400 0.90870400 0.24959300

H -3.20682000 1.26257300 -1.62680000

C -3.92677400 0.13154500 1.50839300

H -2.02967600 -0.61487600 2.28692400

H -4.18947300 1.99284300 0.43096100

H -5.35493300 0.74937100 -0.02924000

H -4.47281900 0.49419500 2.39533400

H -4.20338400 -0.93096400 1.38775100

C 2.40319300 -0.09299100 1.73148700

C 3.44011300 -0.10846200 -0.75466200

C 3.58811000 0.87112800 1.59534600

H 1.61530100 0.33238700 2.37893800

C 4.33406500 0.62587600 0.26963500

H 3.69508300 -1.18536500 -0.72532500

H 3.21566600 1.90848400 1.63292500

H 4.25958300 0.76400900 2.46390600

H 4.68532900 1.58233400 -0.15568300

H 5.24675500 0.03014000 0.43561100

C -3.87473600 -0.78362600 -1.61050900

H -3.16854000 -1.12189100 -2.38507000

H -4.03489300 -1.62618100 -0.91672300

H -4.83746000 -0.57238200 -2.10259000

C -2.01071700 1.52893400 2.45515300

H -2.40625000 2.43607500 1.97038100

H -2.40830000 1.50614600 3.48277700

H -0.91731700 1.64576200 2.52430900

C 3.54328300 0.34156400 -2.19900100

H 3.27592700 1.40131200 -2.33348200

H 4.57743200 0.21771500 -2.55757200

H 2.89441600 -0.25586000 -2.85907100

C 2.80214600 -1.47189300 2.23367500

H 1.94469300 -2.16243400 2.27829800

H 3.57564700 -1.93739100 1.59927100

H 3.22290700 -1.39610900 3.24910400

P 1.74236300 -0.18383700 -0.00440400

P -1.75366600 0.10728800 -0.02528300

**References**

[1] C. K. Mirabelli, D. T. Hill, L. F. Faucette, F. L. McCabe, G. R. Girard, D. B. Bryan, B. M. Sutton, J. O. L. Barus, S. T. Crooke, R. K. Johnson, *J. Med. Chem.* **1987**, *30*, 2181–2190.

[2] S. J. Berners-Price, C. K. Mirabelli, R. K. Johnson, M. R. Mattern, F. L. McCabe, L. F. Faucette, S.-M. Mong, P. J. Sadler, S. T. Crooke, *Cancer Res.* **1986**, *46*, 5486–5493.

[3] J. Yang, V. Giuso, M.-C. Hou, E. Remadna, J. Forté, H.-C. Su, C. Gourlaouen, M. Mauro, B. Bertrand, *Inorg. Chem.* **2023**, *62*, 4903–4921.

[4] B. David, U. Monkowius, J. Rust, C. W. Lehmann, L. Hyzak, F. Mohr, *Dalton Trans.* **2014**, *43*, 11059–11066.

[5] P. Bayat, D. Gatineau, D. Lesage, A. Martinez, R. B. Cole, *J Mass Spectrom* **2022**, *57*, DOI 10.1002/jms.4879.

[6] F. Ichou, A. Schwarzenberg, D. Lesage, S. Alves, C. Junot, X. Machuron-Mandard, J.-C. Tabet, *J. Mass Spectrom.* **2014**, *49*, 498–508.

[7] L. Bourehil, C. Soep, S. Seng, S. Dutrannoy, S. Igoudjil, J. Forté, G. Gontard, D. Lesage, B. Bertrand, H. Dossmann, *Inorg. Chem.* **2023**, *62*, 13304–13314.

[8] L. Drahos, K. Vékey, *J. Mass Spectrom.* **2001**, *36*, 237–263.

[9] O. K. Rice, H. C. Ramsperger, *J. Am. Chem. Soc.* **1927**, *49*, 1617–1629.

[10] R. A. Marcus, O. K. Rice, *J. Phys. Chem.* **1951**, *55*, 894–908.

[11] R. A. Marcus, *J. Chem. Phys.* **1952**, *20*, 359–364.

[12] L. S. Kassel, *J. Phys. Chem.* **1928**, *32*, 225–242.

[13] M. J. Frisch, G. W. Trucks, H. B. Schlegel, G. E. Scuseria, M. A. Robb, J. R. Cheeseman, G. Scalmani, V. Baronne, B. Mennucci, G. A. Petersson, H. Nakatsuji, M. Caricato, X. Li, H. P. Hratchian, A. F. Izmaylov, J. Bloino, G. Zheng, J. L. Sonnenberg, M. Hada, M. Ehara, K. Toyota, R. Fukuda, J. Hasegawa, M. Ishida, T. Nakajima, Y. Honda, O. Kitao, H. Nakai, T. Vreven, J. A. Montgomery, Jr., J. E. Peralta, F. Ogliaro, M. Bearpark, J. J. Heyd, E. Brothers, K. N. Kudin, V. N. Staroverov, R. Kobayashi, J. Normand, K. Raghavachari, A. Rendell, J. C. Burant, S. S. Iyengar, J. Tomasi, M. Cossi, N. Rega, J. M. Millam, M. Klene, J. E. Knox, J. B. Cross, V. Bakken, C. Adamo, J. Jaramillo, R. Gomperts, R. E. Stratmann, O. Yazyev, A. J. Austin, R. Cammi, C. Pomelli, J. W. Ochterski, R. L. Martin, K. Morokuma, V. G. Zakrzewski, G. H. Voth, P. Salvador, J. J. Dannenberg, S. Dapprich, A. D. Daniels, Ö. Farkas, J. B. Foresman, J. V. Ortiz, J. Cioslowski, D. J. Fox, **2016**.

[14] D. Gatineau, H. Dossmann, H. Clavier, A. Memboeuf, L. Drahos, Y. Gimbert, D. Lesage, *International Journal of Mass Spectrometry* **2021**, *463*, 116545.

[15] C. Bissardon, S. Reymond, M. Salomé, L. André, S. Bayat, P. Cloetens, S. Bohic, *JoVE* **2019**, 60461.

[16] F. Villar, L. Andre, R. Baker, S. Bohic, J. C. da Silva, C. Guilloud, O. Hignette, J. Meyer, A. Pacureanu, M. Perez, M. Salome, P. van der Linden, Y. Yang, P. Cloetens, *Synchrotron Radiation News* **2018**, *31*, 9–14.

[17] J. C. da Silva, A. Pacureanu, Y. Yang, S. Bohic, C. Morawe, R. Barrett, P. Cloetens, *Optica, OPTICA* **2017**, *4*, 492–495.

[18] G. Utica, M. Carminati, E. Fabbrica, G. Ticchi, G. Deda, G. Borghi, N. Zorzi, P. Cloetens, C. Cohen, M. Salomé, G. Falkenberg, C. Fiorini, in *2021 IEEE Nuclear Science Symposium and Medical Imaging Conference (NSS/MIC)*, **2021**, pp. 1–3.

[19] O. Proux, E. Lahera, W. Del Net, I. Kieffer, M. Rovezzi, D. Testemale, M. Irar, S. Thomas, A. Aguilar-Tapia, E. F. Bazarkina, A. Prat, M. Tella, M. Auffan, J. Rose, J.-L. Hazemann, *Journal of Environmental Quality* **2017**, *46*, 1146–1157.

[20] F. Porcaro, S. Roudeau, A. Carmona, R. Ortega, *TrAC Trends Anal. Chem.* **2018**, *104*, 22–41.

[21] B. Ravel, M. Newville, *J Synchrotron Rad* **2005**, *12*, 537–541.

[22] O. Bunău, Y. Joly, *J. Phys.: Condens. Matter* **2009**, *21*, 345501.

[23] B. Bertrand, C. Botuha, J. Forté, H. Dossmann, M. Salmain, *Chem. Eur. J.* **2020**, *26*, 12846–12861.

[24] V. Giuso, J. Yang, J. Forté, H. Dossmann, C. Daniel, C. Gourlaouen, M. Mauro, B. Bertrand, *ChemPlusChem* **2023**, *88*, e202300303.
